# Multinational Attitudes Toward AI in Health Care and Diagnostics Among Hospital Patients

**DOI:** 10.1001/jamanetworkopen.2025.14452

**Published:** 2025-06-10

**Authors:** Felix Busch, Lena Hoffmann, Lina Xu, Long Jiang Zhang, Bin Hu, Ignacio García-Juárez, Liz N. Toapanta-Yanchapaxi, Natalia Gorelik, Valérie Gorelik, Gaston A. Rodriguez-Granillo, Carlos Ferrarotti, Nguyen N. Cuong, Chau A. P. Thi, Murat Tuncel, Gürsan Kaya, Sergio M. Solis-Barquero, Maria C. Mendez Avila, Nevena G. Ivanova, Felipe C. Kitamura, Karina Y. I. Hayama, Monserrat L. Puntunet Bates, Pedro Iturralde Torres, Esteban Ortiz-Prado, Juan S. Izquierdo-Condoy, Gilbert M. Schwarz, Jochen G. Hofstaetter, Michihiro Hide, Konagi Takeda, Barbara Perić, Gašper Pilko, Hans O. Thulesius, Thomas Lindow, Israel K. Kolawole, Samuel Adegboyega Olatoke, Andrzej Grzybowski, Alexandru Corlateanu, Oana-Simina Iaconi, Ting Li, Izabela Domitrz, Katarzyna Kępczyńska, Matúš Mihalčin, Lenka Fašaneková, Tomasz Zatoński, Katarzyna Fułek, András Molnár, Stefani Maihoub, Zenewton A. da Silva Gama, Luca Saba, Petros Sountoulides, Marcus R. Makowski, Hugo J. W. L. Aerts, Lisa C. Adams, Keno K. Bressem, and the COMFORT consortium, Álvaro Aceña Navarro, Catarina Águas, Martina Aineseder, Muaed Alomar, Rashid Al Sliman, Gautam Anand, Salita Angkurawaranon, Shuhei Aoki, Samuel Arkoh, Gizem Ashraf, Yesi Astri, Sameer Bakhshi, Nuru Y. Bayramov, Antonis Billis, Almir G. V. Bitencourt, Anetta Bolejko, Antonio J. Bollas Becerra, Joe Bwambale, Andreia Capela, Riccardo Cau, Kelly R. Chacon-Acevedo, Tafadzwa L. Chaunzwa, Rubens Chojniak, Warren Clements, Renato Cuocolo, Victor Dahlblom, Kelienny de Meneses Sousa, Jorge Esteban Villarrubia, Vijay B. Desai, Ajaya K. Dhakal, Virginia Dignum, Rubens G. Feijo Andrade, Giovanna Ferraioli, Shuvadeep Ganguly, Harshit Garg, Cvetanka Gjerakaroska Savevska, Marija Gjerakaroska Radovikj, Anastasia Gkartzoni, Luis Gorospe, Ian Griffin, Martin Hadamitzky, Martin Hakorimana Ndahiro, Alessa Hering, Bruno Hochhegger, Mehriban R. Huseynova, Fujimaro Ishida, Nisha Jha, Lili Jiang, Rawen Kader, Helen Kavnoudias, Clément Klein, George Kolostoumpis, Abraham Koshy, Nicholas A. Kruger, Alexander Löser, Marko Lucijanic, Despoina Mantziari, Gaelle Margue, Sonyia McFadden, Masahiro Miyake, Wipawee Morakote, Issa Ngabonziza, Thao T. Nguyen, Stefan M. Niehues, Marc Nortje, Subish Palaian, Natalia V. Pentara, Rui P. Pereira de Almeida, Gianluigi Poma, Mitayani Purwoko, Nikolaos Pyrgidis, Vasileios Rafailidis, Clare Rainey, João C. Ribeiro, Nicolás Rozo Agudelo, Keina Sado, Julia M. Saidman, Pedro J. Saturno-Hernandez, Vidyani Suryadevara, Gerald B. Schulz, Ena Soric, Javier Soto-Pérez-Olivares, Arnaldo Stanzione, Julian Peter Struck, Hiroyuki Takaoka, Satoru Tanioka, Tran T. M. Huyen, Daniel Truhn, Elon H. C. van Dijk, Peter van Wijngaarden, Yuan-Cheng Wang, Matthias Weidlich, Shuhang Zhang

**Affiliations:** 1 Department of Diagnostic and Interventional Radiology, School of Medicine and Health, Klinikum rechts der Isar, TUM University Hospital, Technical University of Munich, Munich, Germany; 2 Department of Radiology, Charité–Universitätsmedizin Berlin, Corporate Member of Freie Universität Berlin and Humboldt Universität zu Berlin, Berlin, Germany; 3 Department of Radiology, Jinling Hospital, Affiliated Hospital of Medical School, Nanjing University, Nanjing, China; 4 Department of Gastroenterology, National Institute of Medical Sciences and Nutrition Salvador Zubirán, Mexico City, Mexico; 5 Unit of Liver Transplantation, National Institute of Medical Sciences and Nutrition Salvador Zubirán, Mexico City, Mexico; 6 Department of Neurology, National Institute of Medical Sciences and Nutrition Salvador Zubirán, Mexico City, Mexico; 7 Department of Radiology, McGill University Health Center, Montreal, Quebec, Canada; 8 Dawson College, Montreal, Quebec, Canada; 9 Center for Medical Education and Clinical Research-National Council for Scientific and Technical Research (CEMIC-CONICET), Autonomous City of Buenos Aires, Argentina; 10 Department of Diagnostic Imaging, Center for Medical Education and Clinical Research “Norberto Quirno” (CEMIC), Autonomous City of Buenos Aires, Argentina; 11 Radiology Center Hanoi, Medical University Hospital Hanoi, Hanoi, Vietnam; 12 Hanoi Medical University, Hanoi, Vietnam; 13 Department of Nuclear Medicine, Faculty of Medicine, Hacettepe University, Ankara, Turkey; 14 Department of Diagnostic and Therapeutic Imaging, Escuela de Tecnologias en Salud, Universidad de Costa Rica, San José, Costa Rica; 15 Department of Urology, Medical University of Plovdiv, Plovdiv, Bulgaria; 16 St Karidad MHAT, Karidad Medical Health Center, Cardiology, Plovdiv, Bulgaria; 17 Department of General Medicine, Medical University of Plovdiv, Plovdiv, Bulgaria; 18 Department of Radiology, Universidade Federal de São Paulo, São Paulo, Brazil; 19 Diagnósticos da América SA (DASA), São Paulo, Brazil; 20 Unidad de Calidad, National Institute of Cardiology Ignacio Chávez, Mexico City, Mexico; 21 Subdirection of Diagnosis and Treatment, National Institute of Cardiology Ignacio Chávez, Mexico City, Mexico; 22 One Health Research Group, Faculty of Health Science, Universidad de Las Américas, Quito, Ecuador; 23 Department of Orthopaedics and Trauma Surgery, Medical University of Vienna, Vienna, Austria; 24 Michael Ogon Laboratory for Orthopaedic Research, Hospital Vienna-Speising, Vienna, Austria; 25 2nd Department, Orthopaedic Hospital Vienna-Speising, Vienna, Austria; 26 Department of Dermatology, Hiroshima Citizens Hospital, Hiroshima, Japan; 27 Department of Radiology, Hiroshima Citizens Hospital, Hiroshima, Japan; 28 Department of Surgical Oncology, Institute of Oncology Ljubljana, Ljubljana, Slovenia; 29 Faculty of Medicine, University of Ljubljana, Ljubljana, Slovenia; 30 Research and Development Department Region Kronoberg, Växjö, Sweden; 31 Department of Medicine and Optometry, Linnaeus University, Kalmar, Sweden; 32 Department of Clinical Physiology, Research and Development, Växjö Central Hospital, Växjö, Sweden; 33 Department of Clinical Physiology, Clinical Sciences, Lund University, Lund, Sweden; 34 Department of Anesthesia, University of Ilorin/Teaching Hospital, Ilorin, Nigeria; 35 Department of Surgery, University of Ilorin/Teaching Hospital, Ilorin, Nigeria; 36 Institute for Research in Ophthalmology, Foundation for Ophthalmology Development, Poznań, Poland; 37 Department of Respiratory Medicine and Allergology, Nicolae Testemițanu State University of Medicine and Pharmacy, Chișinău, Republic of Moldova; 38 Research Cooperation Unit, Research Department, National Institute of Research in Medicine and Health, Nicolae Testemițanu State University of Medicine and Pharmacy, Chișinău, Republic of Moldova; 39 Department of Rheumatology, Shanghai Jiao Tong University School of Medicine, Renji Hospital, Shanghai, China; 40 Department of Neurology, Faculty of Medicine and Dentistry, Medical University of Warsaw, Warsaw, Poland; 41 Department of Neurology, Bielanski Hospital, Warsaw, Poland; 42 Department of Infectious Diseases, Faculty of Medicine, Masaryk University, Brno, Czech Republic; 43 Department of Infectious Diseases, University Hospital Brno, Brno, Czech Republic; 44 Department of Otolaryngology, Head and Neck Surgery, Wroclaw Medical University, Wroclaw, Poland; 45 Department of Otorhinolaryngology, Head and Neck Surgery, Semmelweis University, Budapest, Hungary; 46 Department of Collective Health, Federal University of Rio Grande do Norte, Natal, Brazil; 47 Department of Radiology, Azienda Ospedaliero Universitaria (A.O.U.), di Cagliari–Polo di Monserrato s.s. 554 Monserrato, Cagliari, Italy; 48 Department of Urology, Aristotle University of Thessaloniki, Thessaloniki, Greece; 49 Artificial Intelligence in Medicine (AIM) Program, Mass General Brigham, Harvard Medical School, Boston, Massachusetts; 50 Department of Radiation Oncology, Dana-Farber Cancer Institute and Brigham and Women’s Hospital, Boston, Massachusetts; 51 Radiology and Nuclear Medicine, CARIM & GROW, Maastricht University, Maastricht, the Netherlands; 52 Department of Radiology, Dana-Farber Cancer Institute and Brigham and Women’s Hospital, Boston, Massachusetts; 53 School of Medicine and Health, Institute for Cardiovascular Radiology and Nuclear Medicine, German Heart Center Munich, TUM University Hospital, Technical University of Munich, Munich, Germany; 54 Department of Cardiology, Hospital Universitario Fundación Jiménez Díaz, Madrid, Spain; 55 Department of Medicine, Universidad Autónoma de Madrid, Madrid, Spain; 56 Department of Radiology, Algarve University Hospital Center, Faro, Portugal; 57 Department of Radiology, Hospital Italiano de Buenos Aires, Autonomous City of Buenos Aires, Argentina; 58 Department of Clinical Sciences, College of Pharmacy and Health Sciences, Ajman University, Ajman, United Arab Emirates; 59 Department of Urology, Faculty of Health Sciences Brandenburg, Brandenburg Medical School Theodor Fontane, Brandenburg an der Havel, Germany; 60 Department of Oncosurgery, Max Institute of Cancer Care, Vaishali, Delhi, India; 61 Department of Radiology, Faculty of Medicine, Chiang Mai University, Chiang Mai, Thailand; 62 Department of Cardiovascular Medicine, Chiba University, Graduate School of Medicine, Chiba, Japan; 63 Department of Radiology, Wenchi Methodist Hospital, Wenchi, Ghana; 64 Centre for Eye Research Australia, Royal Victorian Eye and Ear Hospital, Melbourne, Victoria, Australia; 65 Department of Neurology, Faculty of Medicine, Universitas Muhammadiyah Palembang, Palembang, Indonesia; 66 Department of Medical Oncology, Dr B.R.A. Institute Rotary Cancer Hospital, All India Institute of Medical Sciences, New Delhi, India; 67 Department of I Surgical Diseases, Azerbaijan Medical University, Baku, Azerbaijan; 68 Lab of Medical Physics & Digital Innovation, School of Medicine, Aristotle University of Thessaloniki, Thessaloniki, Greece; 69 Department of Imaging, A.C. Camargo Cancer Center, São Paulo, Brazil; 70 Diagnostic Radiology, Department of Translational Medicine, Lund University, Malmö, Sweden; 71 Department of Medical Imaging and Physiology, Skåne University Hospital, Malmö, Sweden; 72 Department of Cardiology, Hospital Universitario Fundación Jiménez Díaz, Madrid, Spain; 73 Society of Radiography of Uganda, Mulago National Referral Hospital, Mulago, Kampala, Uganda; 74 Department of Medical Oncology, Centro Hospitalar Vila Nova de Gaia-Espinho, Vila Nova de Gaia, Portugal; 75 Associação de Investigação de Cuidados de Suporte em Oncologia (AICSO), Vila Nova de Gaia, Portugal; 76 Department of Radiology, Azienda Ospedaliero Universitaria (A.O.U.), di Cagliari–Polo di Monserrato s.s. 554 Monserrato, Cagliari, Italy; 77 Instituto Global de Excelencia Clínica, Grupo de investigación Traslacional, Keralty, Bogotá D.C., Colombia; 78 Department of Radiation Oncology, Dana-Farber Cancer Institute and Brigham and Women’s Hospital, Boston, Massachusetts; 79 Department of Radiology, Dana-Farber Cancer Institute and Brigham and Women’s Hospital, Boston, Massachusetts; 80 Department of Imaging, A.C. Camargo Cancer Center, São Paulo, Brazil; 81 Department of Radiology, Alfred Health, Melbourne, Victoria, Australia; 82 Department of Surgery, Monash University, Central Clinical School, Melbourne, Victoria, Australia; 83 National Trauma Research Institute, Melbourne, Victoria, Australia; 84 Department of Medicine, Surgery and Dentistry, University of Salerno, Baronissi, Italy; 85 Diagnostic Radiology, Department of Translational Medicine, Lund University, Malmö, Sweden; 86 Department of Collective Health, Federal University of Rio Grande do Norte, Natal, Brazil; 87 Department of Medical Oncology, Hospital Universitario 12 de Octubre, Madrid, Spain; 88 Department of Clinical Sciences, College of Dentistry, Ajman University, Ajman, United Arab Emirates; 89 Department of Pediatrics, KIST Medical College and Teaching Hospital, Kathmandu, Nepal; 90 Department of Computing Science, Umeå University, Umeå, Sweden; 91 Department of Radiology, Pontifical Catholic University of Rio Grande do Sul, Porto Alegre, Brazil; 92 Department of Clinical, Surgical Diagnostic and Pediatric Sciences, University of Pavia, Pavia, Italy; 93 Department of Medical Oncology, Dr B.R.A. Institute Rotary Cancer Hospital, All India Institute of Medical Sciences, New Delhi, India; 94 Department of Urology, Oncology and Robotic Surgery, Max Institute of Cancer Care, Vaishali, Delhi, India; 95 University Clinic for Physical Medicine and Rehabilitation, Ss Cyril and Methodius University, Skopje, Republic of North Macedonia; 96 University Clinic for State Cardiac Surgery, Ss Cyril and Methodius University, Skopje, Republic of North Macedonia; 97 Lab of Medical Physics & Digital Innovation, School of Medicine, Aristotle University of Thessaloniki, Thessaloniki, Greece; 98 Department of Radiology, Ramón y Cajal University Hospital, IRYCIS, Madrid, Spain; 99 Department of Radiology, University of Florida, Gainesville; 100 Institute for Radiology and Nuclear Medicine, German Heart Center Munich, Technical University of Munich, Munich, Germany; 101 Ministry of Health, Byumba Hospital, Byumba, Rwanda; 102 Department of Radiology and Nuclear Medicine, Radboud University Medical Center, Nijmegen, the Netherlands; 103 Fraunhofer MEVIS, Institute for Digital Medicine, Bremen, Germany; 104 Department of Radiology, University of Florida, Gainesville; 105 Department of I Surgical Diseases, Azerbaijan Medical University, Baku, Azerbaijan; 106 Department of Neurosurgery, Mie Chuo Medical Center, Tsu, Japan; 107 Clinical Pharmacology and Therapeutics, KIST Medical College and Teaching Hospital, Kathmandu, Nepal; 108 Department of Computing Science, Umeå University, Umeå, Sweden; 109 Division of Surgery and Interventional Sciences, University College London, London, United Kingdom; 110 Department of Radiology, Alfred Health, Melbourne, Victoria, Australia; 111 Department of Surgery, Monash University, Central Clinical School, Melbourne, Victoria, Australia; 112 National Trauma Research Institute, Melbourne, Victoria, Australia; 113 Department of Neuroscience, Monash University, Central Clinical School, Melbourne, Victoria, Australia; 114 Department of Urology, Bordeaux Pellegrin University Hospital, Bordeaux, France; 115 European Cancer Patient Coalition (ECPC), Brussels, Belgium; 116 Department of Gastroenterology, Lakeshore Hospital, Kochi, India; 117 Orthopaedic Department, University of Cape Town, Cape Town, South Africa; 118 Berlin University of Applied Sciences and Technology (BHT), Berlin, Germany; 119 Department of Hematology, Clinical Hospital Dubrava, Zagreb, Croatia; 120 Department of Internal Medicine, School of Medicine, University of Zagreb, Zagreb, Croatia; 121 Lab of Medical Physics & Digital Innovation, School of Medicine, Aristotle University of Thessaloniki, Thessaloniki, Greece; 122 Department of Urology, Bordeaux Pellegrin University Hospital, Bordeaux, France; 123 School of Health Sciences, Londonderry, Northern Ireland, United Kingdom; 124 Department of Ophthalmology and Visual Sciences, Kyoto University Graduate School of Medicine, Kyoto, Japan; 125 Department of Radiology, Faculty of Medicine, Chiang Mai University, Chiang Mai, Thailand; 126 Ministry of Health, Byumba Hospital, Byumba, Rwanda; 127 Department of Radiology, University of Medicine and Pharmacy, Hue University, Hue, Vietnam; 128 Department of Radiology, Charité–Universitätsmedizin Berlin, Corporate Member of Freie Universität Berlin and Humboldt Universität zu Berlin, Berlin, Germany; 129 Orthopaedic Department, University of Cape Town, Cape Town, South Africa; 130 Department of Clinical Sciences, College of Pharmacy and Health Sciences, Ajman University, Ajman, United Arab Emirates; 131 Department of Clinical Radiology, AHEPA General University Hospital, Aristotle University of Thessaloniki, Thessaloniki, Greece; 132 Department of Radiology, University of Algarve, Faro, Portugal; 133 Comprehensive Health Research Center, University of Évora, Évora, Portugal; 134 Department of Diagnostic and Imaging Services, Fondazione IRCCS Policlinico S. Matteo, Pavia, Italy; 135 Medical Biology, Faculty of Medicine, Universitas Muhammadiyah Palembang, Palembang, Indonesia; 136 Department of Urology, University Hospital, Ludwig-Maximilians-University of Munich, Munich, Germany; 137 Department of Clinical Radiology, AHEPA General University Hospital, Aristotle University of Thessaloniki, Thessaloniki, Greece; 138 School of Health Sciences, Londonderry, Northern Ireland, United Kingdom; 139 Department of Otolaryngology, Coimbra University and Medical School, Coimbra, Portugal; 140 Instituto Global de Excelencia Clínica, Grupo de investigación Traslacional, Keralty, Bogotá D.C., Colombia; 141 Department of Ophthalmology and Visual Sciences, Kyoto University Graduate School of Medicine, Kyoto, Japan; 142 Department of Radiology, Hospital Italiano de Buenos Aires, Ciudad Autónoma de Buenos Aires, Argentina; 143 AXA Chair in Healthcare Quality, CIEE, National Institute of Public Health, Cuernavaca, Mexico; 144 Department of Radiology, Molecular Imaging Program at Stanford (MIPS), Stanford University School of Medicine, Stanford, California; 145 Department of Urology, University Hospital, Ludwig-Maximilians-University of Munich, Munich, Germany; 146 Department of Hematology, Clinical Hospital Dubrava, Zagreb, Croatia; 147 Department of Radiology, Ramón y Cajal University Hospital, IRYCIS, Madrid, Spain; 148 Department of Advanced Biomedical Sciences, University of Naples “Federico II”, Naples, Italy; 149 Department of Urology, Faculty of Health Sciences Brandenburg, Brandenburg Medical School Theodor Fontane, Brandenburg an der Havel, Germany; 150 Department of Cardiology, Chiba University Hospital, Chiba, Japan; 151 Department of Neurosurgery, Mie University Graduate School of Medicine, Tsu, Japan; 152 Charité Lab for Artificial Intelligence in Medicine, Corporate Member of Freie Universität Berlin and Humboldt Universität zu Berlin, Berlin, Germany; 153 Department of Radiology, University of Medicine and Pharmacy, Hue University, Hue, Vietnam; 154 Department of Diagnostic and Interventional Radiology, University Hospital Aachen, Aachen, Germany; 155 Department of Ophthalmology, Leiden University Medical Center, Leiden, the Netherlands; 156 Department of Ophthalmology, Alrijne Hospital, Leiderdorp, the Netherlands; 157 Centre for Eye Research Australia, Royal Victorian Eye and Ear Hospital, Melbourne, Victoria, Australia; 158 Ophthalmology, Department of Surgery, University of Melbourne, Melbourne, Victoria, Australia; 159 Department of Radiology, Zhongda Hospital Southeast University, Nanjing, China; 160 Department of Radiology, Charité–Universitätsmedizin Berlin, Corporate Member of Freie Universität Berlin and Humboldt Universität zu Berlin, Berlin, Germany; 161 Department of Radiology, Zhongda Hospital Southeast University, Nanjing, China

## Abstract

**Importance:**

The successful implementation of artificial intelligence (AI) in health care depends on its acceptance by key stakeholders, particularly patients, who are the primary beneficiaries of AI-driven outcomes.

**Objectives:**

To survey hospital patients to investigate their trust, concerns, and preferences toward the use of AI in health care and diagnostics and to assess the sociodemographic factors associated with patient attitudes.

**Design, Setting, and Participants:**

This cross-sectional study developed and implemented an anonymous quantitative survey between February 1 and November 1, 2023, using a nonprobability sample at 74 hospitals in 43 countries. Participants included hospital patients 18 years of age or older who agreed with voluntary participation in the survey presented in 1 of 26 languages.

**Exposure:**

Information sheets and paper surveys handed out by hospital staff and posted in conspicuous hospital locations.

**Main Outcomes and Measures:**

The primary outcome was participant responses to a 26-item instrument containing a general data section (8 items) and 3 dimensions (trust in AI, AI and diagnosis, preferences and concerns toward AI) with 6 items each. Subgroup analyses used cumulative link mixed and binary mixed-effects models.

**Results:**

In total, 13 806 patients participated, including 8951 (64.8%) in the Global North and 4855 (35.2%) in the Global South. Their median (IQR) age was 48 (34-62) years, and 6973 (50.5%) were male. The survey results indicated a predominantly favorable general view of AI in health care, with 57.6% of respondents (7775 of 13 502) expressing a positive attitude. However, attitudes exhibited notable variation based on demographic characteristics, health status, and technological literacy. Female respondents (3511 of 6318 [55.6%]) exhibited fewer positive attitudes toward AI use in medicine than male respondents (4057 of 6864 [59.1%]), and participants with poorer health status exhibited fewer positive attitudes toward AI use in medicine (eg, 58 of 199 [29.2%] with rather negative views) than patients with very good health (eg, 134 of 2538 [5.3%] with rather negative views). Conversely, higher levels of AI knowledge and frequent use of technology devices were associated with more positive attitudes. Notably, fewer than half of the participants expressed positive attitudes regarding all items pertaining to trust in AI. The lowest level of trust was observed for the accuracy of AI in providing information regarding treatment responses (5637 of 13 480 respondents [41.8%] trusted AI). Patients preferred explainable AI (8816 of 12 563 [70.2%]) and physician-led decision-making (9222 of 12 652 [72.9%]), even if it meant slightly compromised accuracy.

**Conclusions and Relevance:**

In this cross-sectional study of patient attitudes toward AI use in health care across 6 continents, findings indicated that tailored AI implementation strategies should take patient demographics, health status, and preferences for explainable AI and physician oversight into account.

## Key Points

**Question:**

What are hospital patients’ attitudes toward the use of artificial intelligence (AI) in health care across diverse sociodemographic contexts?

**Findings:**

This cross-sectional study surveying 13 806 patients using a nonprobability sample from 74 hospitals in 43 countries found that while patients were generally supportive of AI-enabled health care facilities and recognized the potential of AI, they preferred explainable AI systems and physician-led decision-making. In addition, attitudes varied significantly by sociodemographic characteristics.

**Meaning:**

These findings highlight the global imperative for health care AI stakeholders to tailor AI implementation to the unique characteristics of individual patients and local populations and provide guidance on how to optimize patient-centered AI adoption.

## Teaser

This cross-sectional survey study conducted in 74 hospitals across 43 countries investigates the trust, concerns, and preferences toward the use of artificial intelligence in health care and diagnostics and the sociodemographic factors that may be associated with these preferences among hospital patients.

## Introduction

Artificial intelligence (AI) has become increasingly prevalent in various industries and public sectors, including health care.^1^ In particular, the development of large language models has intensified public discourse on the potential impact of AI, especially in health care.^2^

AI technologies offer promising solutions to pressing health care challenges, including staff shortages, high administrative costs, and economic constraints.^3^ In clinical practice, AI applications range from assisting with image-based diagnoses to personalizing treatment strategies and predicting risk factors and therapy responses.^4,5^ Beyond direct patient care, AI facilitates drug discovery and development while streamlining administrative tasks, such as data extraction, curation, and report structuring.^6,7^

The economic implications are substantial, with projections suggesting that AI technologies could reduce health care spending in the US by 5% to 10%, potentially yielding annual savings of $200 to $360 billion.^8^ The rapid integration of AI in health care is further evidenced by the US Food and Drug Administration’s approval of 692 AI-enabled and machine learning–enabled medical devices through July 2023, with 478 (69.1%) approved in just the past 3 years.^9^

Despite this rapid growth, the benefits of AI applications to patient care are not always clear.^10^ While patient acceptance is important for the sustainable adoption of AI, patients may not always have the opportunity to consent to its use.^11,12^ In addressing this challenge, adopting biopsychosocial perspectives that recognize patients’ unique experiences, beliefs, and values in health maintenance can help steer AI toward patient-centered care.^11,12,13,14^ Moreover, fostering patient trust in AI is vital, as it may positively influence adherence to AI-assisted care and related health outcomes, as demonstrated in conditions such as diabetes management.^15,16^

Exploring patient perspectives can, therefore, be highly beneficial in ensuring the successful integration of AI in health care. Patients whose health is directly affected by AI, either through improved treatment and diagnosis or by potential consequences of immature AI, may hold views that diverge substantially from those of clinicians.

However, a notable knowledge gap exists regarding patient attitudes, particularly on a large, international scale. To date, existing studies are limited to data from 1 or at most 2 countries, failing to capture the likely variations in patient attitudes across different sociodemographic contexts.^17,18,19,20,21,22,23,24^

To address these challenges, we conducted the first, to our knowledge, large-scale, international, multicenter survey of hospital patients to investigate patients’ trust, concerns, and preferences toward AI use in health care and diagnostics and to assess factors that may influence patient attitudes. By focusing on the input of patients from diverse global contexts, including from the Global North and Global South, this study aimed to provide a comprehensive, global perspective on patient attitudes toward AI use in health care, thereby contributing to work focused on the development of patient-centered AI applications.

## Methods

### Study Design

This multicenter, international, cross-sectional study was conducted in accordance with the Strengthening the Reporting of Observational Studies in Epidemiology (STROBE) reporting guideline (eTable 1 in Supplement 1) and the American Association for Public Opinion Research best practices for survey research. Ethical approval was obtained from Charité–Universitätsmedizin Berlin, which served as the lead institution, and from all other participating hospitals according to their institutional policies (eTable 2 in Supplement 1). Given the unsupervised and anonymous design of the instrument, informed consent was waived by the lead institution to preserve participant anonymity.

### Setting and Participants

The survey was administered to a nonprobability convenience sample at 74 COMFORT network hospitals across 43 countries. Local staff disseminated the surveys, which were also displayed in prominent areas, such as waiting rooms, from February 1 to November 1, 2023. We targeted radiology departments as the primary site for the survey because of the high turnover of patients with a wide range of conditions. Participants could submit their responses through drop boxes or directly to staff. Collected data from all participating sites were then centrally analyzed at Charité–Universitätsmedizin Berlin. The sample size was determined using the Cochran formula. Assuming a 50% response distribution (which was chosen because it allows for the most conservative estimate), a 95% CI, and 5% margin of error, we determined a minimum sample size of 385 respondents. Inclusion criteria were patients aged 18 years or older who attended a participating department during the study period, agreed to participate in the survey voluntarily, and were able to complete the questionnaire independently in 1 of 26 local languages (Azerbaijani, Bahasa Indonesia, Bulgarian, Croatian, Czech, Dutch, English, French, German, Greek, Hindi, Hungarian, Italian, Japanese, Macedonian, Malayalam, Mandarin, Polish, Portuguese, Romanian, Slovenian, Spanish, Swedish, Thai, Turkish, and Vietnamese). Patients who did not complete any items or only items capturing variables for sample stratification were excluded.

### Survey Development and Design

To inform the survey construct for our measures on patient attitudes toward the use of AI in health care and diagnostics, we followed the systematic review by Young et al^17^ synthesizing 23 qualitative, quantitative, or mixed-methods original articles on patient and public attitudes toward clinical AI. In addition, a sample of 10 voluntary patients who visited the Department of Radiology at Charité–Universitätsmedizin Berlin in January 2023 were interviewed to explore how patients understood and conceptualized the construct, starting with an unprompted discussion followed by focused questions on our measures. Based on the systematic review and semistructured interviews, a multidisciplinary expert panel from the COMFORT consortium, including patient representatives, radiologists, urologists, medical faculty members and educators, AI researchers and developers, and biomedical ethicists and statisticians (F.B., L.H., P.S., L.C.A., K.K.B., A.B., R.C., V.D., A.H., L.J., G.K., and A.L.) from 7 countries, developed a 26-item survey. The comprehensibility and overall length of our instrument were evaluated in cognitive interviews with 10 patients at the Department of Radiology, Charité–Universitätsmedizin Berlin, followed by a pilot study to test the internal reliability, consistency, and unidimensionality.

The pilot study group consisted of 100 patients visiting the Department of Radiology, Charité–Universitätsmedizin Berlin in January 2023. Psychometric validation of the questionnaire was performed using R version 4.2.2 (R Project for Statistical Computing), including the packages tidyverse (version 1.3.2), lavaan (version 0.6-13), and psych (version 2.2.9). Baseline characteristics of the pilot study group are displayed in eTable 3 in Supplement 1.^25,26,27^

The internal consistency of the questionnaire was assessed using the Cronbach α. The following 3 scales with 6 items each were evaluated: trust in AI, AI and diagnosis, and preferences and concerns toward AI. The trust in AI scale demonstrated excellent internal consistency (α = .94), and the AI and diagnosis and the preferences and concerns toward AI scales showed good consistency (α = .80 and α = .86, respectively).

The Kaiser-Meyer-Olkin measure and the Bartlett test of sphericity further validated the appropriateness of the data for factor analysis. The Kaiser-Meyer-Olkin measure was 0.93, indicating that sampling was adequate and that the data were suitable for factor analysis. The Bartlett test of sphericity yielded a significant result (*P* < .001), confirming that the variables were sufficiently correlated for factor analysis. Confirmatory factor analysis was used to assess the construct validity of the questionnaire. Model fit indices indicated a reasonable fit to the data, with a comparative fit index of 0.956 and a Tucker-Lewis index of 0.949, both above the recommended threshold of 0.900. The root mean square error of approximation was 0.066, and the standardized root mean square residual was 0.055, indicating a good model fit. Factor loadings were significant, indicating strong associations between items and their respective latent constructs.

### Variables

The instrument consisted of 3 dimensions: trust in AI, AI and diagnosis, and preferences and concerns toward AI, each with 6 items, complemented by a general data section with 8 items (self-reported gender, age, highest educational level, weekly use of technological devices, health status, AI knowledge, and general attitudes toward AI use in medicine and health care). We chose to collect data on gender rather than sex to allow for a more inclusive data collection that recognizes diverse individuals and reflects social identities, roles, and experiences that are not captured by biological characteristics. Gender categories were male, female, and diverse.

The trust in AI dimension measured confidence in AI improving health care, trust in AI providing information about health, diagnosis, response to treatment and making vital decisions, and agreement with the use of AI depending on disease severity by using 4- and 5-point Likert scale items. The AI and diagnosis dimension assessed attitudes toward AI analyzing radiographs and cancer, its role as a second opinion for physicians, trade-offs in diagnostic accuracy, and diagnostic preferences if AI and physicians would have equal accuracy by using 4- and 5-point Likert scale items and 2 multinomial items. The preferences and concerns toward AI dimension assessed attitudes on the use of AI in health care facilities, preference for visiting such facilities, and concerns about the influence of AI on cost, data security, physician-patient interaction, and replacement of human physicians by using 4- and 5-point Likert scale items.

### Statistical Analysis

Statistical analyses were conducted using R, version 4.3.1, with the packages tidyverse (version 2.0.0), ordinal (version 2023.12-4), and lme4 (version 1.1-35-1) for data manipulation and modeling.^25,28,29^ Survey results were summarized using frequencies and percentages for the total cohort, the Global North and Global South based on the definitions of the United Nations Finance Center for South-South Cooperation, and by continent according to the location of the hospital visited using the United Nations Geoscheme.^30,31^ To assess differences between patient groups (categorized by gender, age, highest educational level, number of technical devices used weekly, AI knowledge, and health status), we used cumulative link mixed models and binary mixed-effects models. Cumulative link mixed models were used for ordinal response items to account for the ordered nature of the responses while considering both the grouping factors as fixed effects and the collection site as a random effect to control for site-specific clustering. We observed substantial site-level heterogeneity (intraclass correlation = 0.22). For questions with categorical outcomes, binomial logistic regression models were fitted, using a strategy of 1 vs the remainder. Cases with missing data were excluded from the respective analyses. Adjusted *P* values were calculated using a Bonferroni correction to address the issue of multiple comparisons. An adjusted 2-sided *P* < .05 was considered statistically significant.

## Results

### Participants

**Figure 1.**
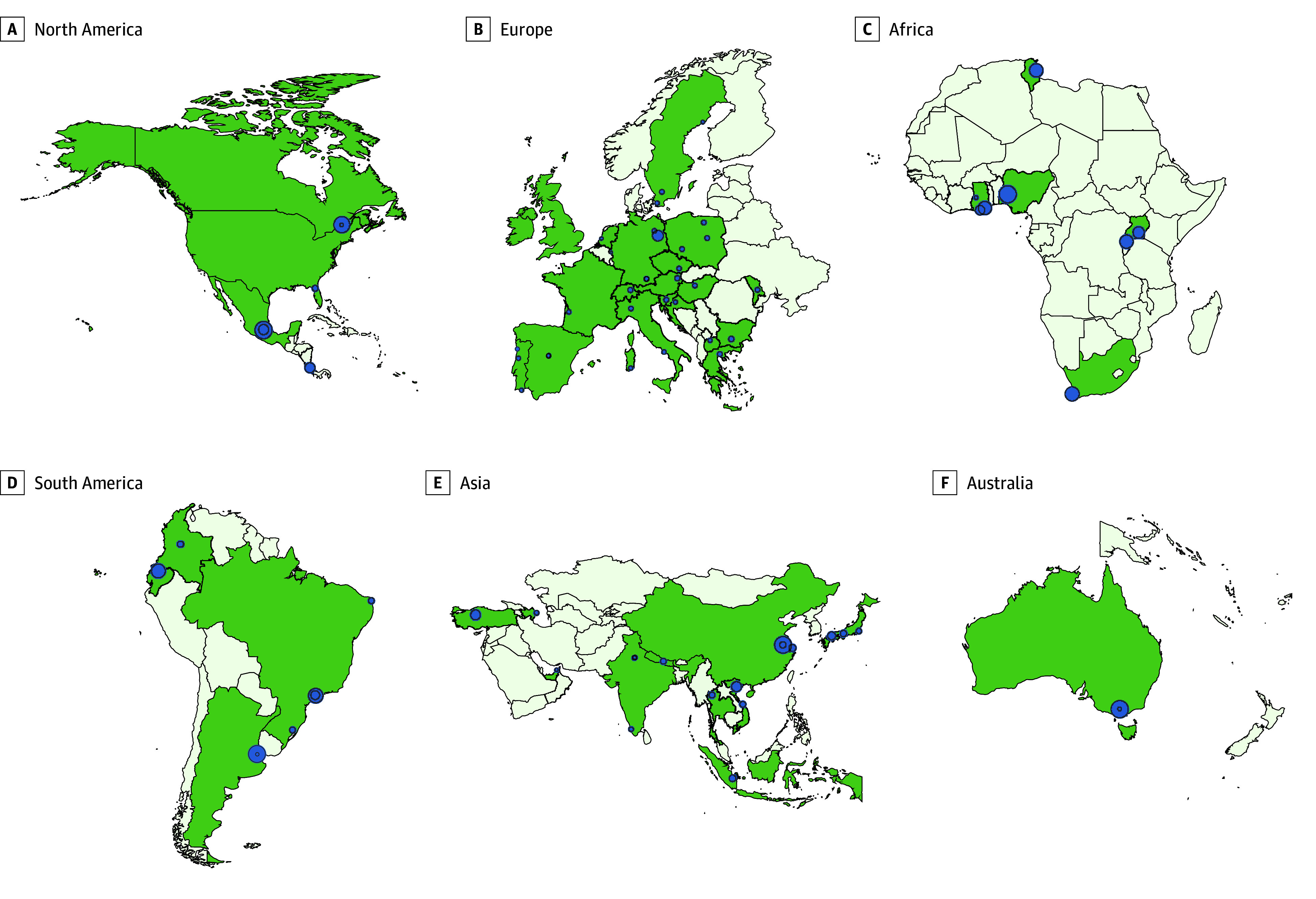
Geographical Distribution of Participating Institutions on World Map The size of the blue dots refers to the proportion of respondents per institution relative to the total number of respondents. Countries with at least 1 participating institution are highlighted in green.

A total of 13 955 surveys were collected, of which 1.1% (n = 149) were excluded from analysis due to no response to any item (n = 12 [0.1%]) or only to items in the general data section (n = 137 [1.0%]) (characteristics of excluded patients are given in eTable 4 in Supplement 1). Of 13 806 patients included, most of their surveys were collected in radiology departments (n = 7081 [51.3%]), followed by gastroenterology (n = 1098 [8.0%]), cardiology (n = 743 [5.4%]), and 21 other specialty departments (n = 4884 [35.4%]). Most patients (n = 8951 [64.8%]) visited hospitals in the Global North. Europe accounted for 41.7% of patients (n = 5764), followed by Asia (n = 3473 [25.2%]), North America (n = 2284 [16.5%]), South America (n = 1336 [9.7%]), Africa (n = 728 [5.3%]), and Australia (n = 221 [1.6%]). Figure 1 illustrates the geographical distribution of participating institutions. A detailed list of participating institutions and corresponding departments and patient numbers is in eTable 5 in Supplement 1.

Sociodemographic profiles and technology literacy, health status, and AI knowledge are shown in Table 1 for the overall study population (median [IQR] age, 48 [34-62] years; 6451 [46.7%] female, 32 [0.2%] gender diverse, 6973 [50.5%] male, and 350 [2.5%] gender not reported) and the region-specific subgroups. Regional breakdowns for each item response are presented in Table 2.

**Table 1.** Sociodemographic Data and Scores on Technological Literacy, Health Status, and AI Knowledge Items for the Total Study Population and Regional Breakdowns by Global North or South and Continents

Characteristic	Patients, No. (%)								
	Total (N = 13 806)	Global North (n = 8951)	Global South (n = 4855)	Europe (n = 5764)	Asia (n = 3473)	North America (n = 2284)	South America (n = 1336)	Africa (n = 728)	Australia (n = 221)
Gender									
Female	6451 (46.7)	4163 (46.5)	2288 (47.1)	2636 (45.7)	1472 (42.4)	1101 (48.2)	809 (60.6)	342 (47.0)	91 (41.2)
Male	6973 (50.5)	4639 (51.8)	2334 (48.1)	3008 (52.2)	1812 (52.2)	1137 (49.8)	516 (38.6)	375 (51.5)	125 (56.6)
Diverse	32 (0.2)	22 (0.2)	10 (0.2)	18 (0.3)	4 (0.1)	2 (0.1)	2 (0.2)	4 (0.6)	2 (0.9)
Not reported	350 (2.5)	127 (1.4)	223 (4.6)	102 (1.8)	185 (5.3)	44 (1.9)	9 (0.7)	7 (1.0)	3 (1.4)
Age, median (IQR), y	48 (34-62)	54 (39-66)	39 (28-52)	55 (39-67)	45 (31-59)	48 (36-61)	42 (30-55)	32 (25-42)	55 (37-66)
Not reported	1354 (9.8)	877 (9.8)	477 (9.8)	639 (11.1)	392 (11.3)	226 (9.9)	62 (4.6)	22 (3.0)	13 (5.9)
Highest educational level									
Elementary school diploma	1192 (8.6)	780 (8.7)	412 (8.5)	684 (11.9)	224 (6.4)	96 (4.2)	139 (10.4)	39 (5.4)	10 (4.5)
Middle school diploma	2844 (20.6)	1868 (20.9)	976 (20.1)	1416 (24.6)	463 (13.3)	448 (19.6)	464 (34.7)	46 (6.3)	7 (3.2)
High school diploma	4142 (30.0)	2790 (31.2)	1352 (27.8)	1431 (24.8)	998 (28.7)	946 (41.4)	449 (33.6)	229 (31.5)	89 (40.3)
University degree	5403 (39.1)	3346 (37.4)	2057 (42.4)	2089 (36.2)	1747 (50.3)	776 (34.0)	277 (20.7)	402 (55.2)	112 (50.7)
Not reported	225 (1.6)	167 (1.9)	58 (1.2)	144 (2.5)	41 (1.2)	18 (0.8)	7 (0.5)	12 (1.7)	3 (1.4)
Use of technical devices^a^									
Total No., median (IQR)	2 (1-3)	2 (1-3)	1 (1-2)	2 (1-3)	1 (1-2)	2 (1-3)	2 (1-2)	1 (1-2)	2 (2-3)
Smartphone	12554 (90.9)	8092 (90.4)	4462 (91.9)	5154 (89.4)	3203 (92.2)	2087 (91.4)	1244 (93.1)	661 (90.8)	205 (92.8)
PC or laptop	7902 (57.2)	5724 (64.0)	2178 (44.9)	3896 (67.6)	1400 (40.3)	1321 (57.8)	769 (57.6)	367 (50.4)	149 (67.4)
Game console	924 (6.7)	609 (6.8)	315 (6.5)	361 (6.3)	200 (5.8)	196 (8.6)	104 (7.8)	40 (5.5)	23 (10.4)
Tablet	3344 (24.2)	2519 (28.1)	825 (17.0)	1620 (28.1)	595 (17.1)	748 (32.8)	209 (15.6)	103 (14.1)	69 (31.2)
E-reader	907 (6.6)	677 (7.6)	230 (4.7)	463 (8.0)	144 (4.2)	175 (7.7)	87 (6.5)	25 (3.4)	13 (5.9)
Smartwatch	2443 (17.7)	1797 (20.1)	646 (13.3)	1183 (20.5)	423 (12.2)	495 (21.7)	222 (16.6)	56 (7.7)	64 (29.0)
None	363 (2.6)	265 (3.0)	98 (2.0)	202 (3.5)	79 (2.3)	39 (1.7)	32 (2.4)	10 (1.4)	1 (0.4)
Not reported	54 (0.4)	52 (0.6)	2 (0.0)	46 (0.8)	0	6 (0.3)	1 (0.1)	1 (0.1)	0
Health status^b^									
Very poor	203 (1.5)	66 (0.7)	137 (2.8)	56 (1.0)	48 (1.4)	3 (0.1)	35 (2.6)	57 (7.8)	4 (1.1)
Poor	1221 (8.8)	711 (7.9)	510 (10.5)	488 (8.5)	380 (10.9)	143 (6.3)	130 (9.7)	65 (8.9)	15 (6.8)
Sufficient	4244 (30.7)	2798 (31.3)	1446 (29.8)	1679 (29.1)	1319 (38.0)	652 (28.6)	454 (34.0)	107 (14.7)	33 (14.9)
Good	5439 (39.4)	3811 (42.6)	1628 (33.5)	2546 (44.2)	1150 (33.1)	966 (42.3)	442 (33.1)	237 (32.6)	98 (44.3)
Very good	2553 (18.5)	1448 (16.2)	1105 (22.8)	891 (1556)	559 (16.1)	502 (22.0)	273 (20.4)	258 (35.4)	70 (31.7)
Not reported	146 (1.1)	117 (1.3)	29 (0.6)	104 (1.8)	17 (0.5)	18 (0.8)	2 (0.2)	4 (0.6)	1 (0.4)
AI knowledge^c^									
No knowledge (never heard of AI)	1848 (13.4)	927 (10.4)	921 (19.0)	629 (10.9)	692 (19.9)	219 (9.6)	194 (14.5)	97 (13.3)	17 (7.7)
Little knowledge (eg, documentary seen on television)	8097 (58.6)	5459 (61.0)	2638 (54.3)	3530 (61.2)	2075 (59.8)	1273 (55.7)	751 (56.2)	349 (47.9)	119 (53.8)
Good knowledge (eg, read several articles about AI)	3423 (24.8)	2311 (25.8)	1112 (22.9)	1414 (24.5)	649 (18.7)	724 (31.7)	306 (22.9)	251 (34.5)	79 (35.8)
Expert (eg, involved in AI development)	211 (1.5)	131 (1.5)	80 (1.6)	80 (1.4)	36 (1.0)	47 (2.1)	17 (1.3)	25 (3.4)	6 (2.7)
Not reported	227 (1.6)	123 (1.4)	104 (2.1)	111 (1.9)	21 (0.6)	21 (0.9)	68 (5.1)	6 (0.8)	0

Abbreviations: AI, artificial intelligence; PC, personal computer.

^a^ Item: Which of these technical devices do you use at least once a week?

^b^ Item: What is your current general state of health?

^c^ Item: How would you rate your knowledge of AI?

**Table 2.** Absolute Survey Results and Regional Breakdowns for Each Item

Question^b^	Patients, No. (%)^a^								
	Total (n = 13 806)	Global North (n = 8951)	Global South (n = 4855)	Europe (n = 5764)	Asia (n = 3473)	North America (n = 2284)	South America (n = 1336)	Africa (n = 728)	Australia (n = 221)
Q1	13 502 (97.8)	8787 (98.2)	4715 (97.1)	5617 (97.4)	3427 (98.7)	2260 (98.9)	1261 (94.4)	721 (99.0)	216 (97.7)
Extremely negative	296 (2.2)	137 (1.6)	159 (3.4)	86 (1.5)	78 (2.3)	33 (1.5)	31 (2.5)	63 (8.7)	5 (2.3)
Rather negative	1033 (7.7)	657 (7.5)	376 (8.0)	448 (8.0)	246 (7.2)	142 (6.3)	108 (8.6)	68 (9.4)	21 (9.3)
Neutral	4398 (32.6)	3019 (34.4)	1379 (29.2)	1980 (35.2)	987 (28.8)	663 (29.3)	457 (36.2)	225 (31.2)	86 (39.8)
Rather positive	5554 (41.1)	3787 (43.1)	1767 (37.5)	2389 (42.5)	1384 (40.4)	1023 (45.3)	458 (36.3)	218 (30.2)	82 (38.0)
Extremely positive	2221 (16.4)	1187 (13.5)	1034 (21.9)	714 (12.7)	732 (21.4)	399 (17.7)	207 (16.4)	147 (20.4)	22 (10.2)
Q2	13 314 (96.4)	8627 (96.4)	4687 (96.5)	5477 (95.0)	3416 (98.4)	2167 (94.9)	1319 (98.7)	717 (98.5)	218 (98.6)
Completely disagree	428 (3.2)	207 (2.4)	221 (4.7)	118 (2.2)	112 (3.3)	55 (2.5)	73 (5.5)	61 (8.5)	9 (4.1)
Tend to disagree	1059 (8.0)	645 (7.5)	414 (8.8)	459 (8.4)	243 (7.1)	119 (5.5)	129 (9.8)	85 (11.9)	24 (11.0)
Neutral	3446 (26.0)	2480 (28.8)	966 (20.6)	1668 (30.4)	804 (23.5)	495 (22.8)	248 (18.8)	154 (21.5)	77 (35.3)
Tend to agree	5162 (38.8)	3482 (40.4)	1680 (35.8)	2200 (40.2)	1303 (38.1)	835 (38.5)	532 (40.3)	209 (29.2)	83 (38.1)
Agree completely	3219 (24.2)	1813 (21.0)	1406 (30.0)	1032 (18.8)	954 (27.9)	663 (30.6)	337 (25.6)	208 (29.0)	25 (11.5)
Q3	13 542 (98.1)	8753 (97.8)	4789 (98.6)	5593 (97.0)	3423 (98.6)	2252 (98.6)	1326 (99.2)	727 (99.9)	221 (100.0)
Very little	528 (3.9)	323 (3.7)	205 (4.3)	202 (3.6)	115 (3.4)	75 (3.3)	47 (3.5)	76 (10.5)	13 (5.9)
Little	1545 (11.4)	902 (10.3)	643 (13.4)	577 (10.3)	374 (10.9)	228 (10.1)	232 (17.5)	106 (14.6)	28 (12.7)
Medium	4896 (36.2)	3346 (38.2)	1550 (32.4)	2200 (39.3)	1277 (37.3)	682 (30.3)	469 (35.4)	186 (25.6)	82 (37.1)
Much	4566 (33.72)	3104 (35.5)	1462 (30.5)	1993 (35.6)	987 (28.8)	895 (39.7)	422 (31.8)	213 (29.3)	56 (25.3)
Very much	2007 (14.8)	1078 (12.3)	929 (19.4)	621 (11.1)	670 (19.6)	372 (16.5)	156 (11.8)	146 (20.1)	42 (19.0)
Q4	13 507 (97.8)	8730 (97.5)	4777 (98.4)	5573 (96.7)	3420 (98.5)	2245 (98.3)	1323 (99.0)	726 (99.7)	220 (99.6)
Very little	564 (4.2)	359 (4.1)	205 (4.3)	219 (3.9)	97 (2.8)	98 (4.4)	58 (4.4)	74 (10.2)	18 (8.2)
Little	1859 (13.8)	1083 (12.4)	776 (16.2)	719 (12.9)	478 (14.0)	275 (12.3)	249 (18.8)	104 (14.3)	34 (15.4)
Medium	5149 (38.1)	3569 (40.9)	1580 (33.1)	2455 (44.0)	1158 (33.9)	721 (32.1)	501 (37.9)	227 (31.3)	87 (39.6)
Much	4587 (34.0)	3075 (35.2)	1512 (31.6)	1804 (32.4)	1196 (35.0)	906 (40.4)	399 (30.2)	218 (30.0)	64 (29.1)
Very much	1348 (10.0)	644 (7.4)	704 (14.7)	376 (6.8)	491 (14.4)	245 (10.9)	116 (8.8)	103 (14.2)	17 (7.7)
Q5	13496 (97.8)	8716 (97.4)	4780 (98.5)	5561 (96.5)	3426 (98.7)	2242 (98.2)	1322 (99.0)	726 (99.7)	219 (99.1)
Very little	597 (4.4)	401 (4.6)	196 (4.1)	236 (4.2)	106 (3.1)	112 (5.0)	61 (4.6)	60 (8.3)	22 (10.1)
Little	1919 (14.2)	1110 (12.7)	809 (16.9)	750 (13.5)	473 (13.1)	282 (12.6)	253 (19.1)	134 (18.5)	27 (12.3)
Medium	5093 (37.7)	3514 (40.3)	1579 (33.0)	2395 (43.1)	1156 (33.7)	746 (33.3)	483 (36.5)	225 (31.0)	88 (40.2)
Much	4516 (33.5)	3027 (34.7)	1489 (31.2)	1796 (32.3)	1216 (35.5)	846 (37.7)	392 (29.6)	206 (28.4)	60 (27.4)
Very much	1371 (10.2)	664 (7.6)	707 (14.8)	384 (6.9)	475 (13.9)	256 (11.4)	133 (10.1)	101 (13.9)	22 (10.1)
Q6	13 480 (97.6)	8704 (97.2)	4776 (98.4)	5551 (96.3)	3423 (98.6)	2238 (98.0)	1322 (99.0)	726 (99.7)	220 (99.6)
Very little	614 (4.6)	424 (4.9)	190 (4.0)	261 (4.7)	105 (3.1)	113 (5.1)	50 (3.8)	63 (8.7)	22 (10.0)
Little	1962 (14.6)	1183 (13.6)	779 (16.3)	777 (14.0)	459 (13.4)	308 (13.8)	252 (19.1)	123 (16.9)	43 (19.6)
Medium	5267 (39.1)	3601 (41.4)	1666 (35.0)	2493 (44.9)	1209 (35.3)	723 (32.3)	522 (39.5)	243 (33.5)	77 (35.0)
Much	4346 (32.2)	2894 (33.2)	1452 (30.4)	1675 (30.2)	1187 (34.7)	853 (38.1)	381 (28.8)	191 (26.3)	59 (26.8)
Very much	1291 (9.6)	602 (6.9)	689 (14.4)	345 (6.2)	463 (13.5)	241 (10.8)	117 (8.9)	106 (14.6)	19 (8.6)
Q7	13 139 (95.2)	8439 (94.3)	4700 (96.8)	5361 (93.0)	3381 (97.4)	2179 (95.4)	1298 (97.2)	711 (97.7)	209 (94.6)
No use of AI independent of the disease	1499 (11.4)	876 (10.4)	623 (13.3)	546 (10.2)	388 (11.5)	237 (10.9)	174 (13.4)	131 (18.4)	23 (11.0)
Use only for minor illnesses (eg, cold)	3661 (27.9)	2101 (24.9)	1560 (33.2)	1366 (25.5)	1094 (32.4)	509 (23.4)	444 (34.2)	198 (27.9)	50 (23.9)
Use also for moderately severe diseases (eg, appendicitis)	3510 (26.7)	2336 (27.7)	1174 (25.0)	1444 (26.9)	947 (28.0)	643 (29.5)	247 (19.0)	168 (23.6)	61 (29.2)
Use also for severe diseases (eg, cancer, traffic accidents)	4469 (34.0)	3126 (37.0)	1343 (28.6)	2005 (37.4)	952 (28.2)	790 (36.3)	433 (33.4)	214 (30.1)	75 (35.9)
Q8	13 437 (97.3)	8668 (96.8)	4769 (98.2)	5522 (95.8)	3423 (98.6)	2225 (97.4)	1323 (99.0)	725 (99.6)	219 (99.1)
Completely disagree	1411 (10.5)	984 (11.4)	427 (9.0)	677 (12.3)	123 (3.6)	236 (10.6)	258 (19.5)	80 (11.0)	37 (16.9)
Tend to disagree	2609 (19.4)	1829 (21.1)	780 (16.4)	1235 (22.4)	443 (12.9)	475 (21.4)	281 (21.2)	136 (18.8)	39 (17.8)
Neutral	3659 (27.2)	2502 (28.9)	1157 (24.3)	1630 (29.5)	988 (28.9)	511 (23.0)	267 (20.2)	201 (27.7)	62 (28.3)
Tend to agree	4374 (32.6)	2716 (31.3)	1658 (34.8)	1590 (28.8)	1339 (39.1)	776 (34.9)	388 (29.3)	212 (29.2)	69 (31.5)
Agree completely	1384 (10.3)	637 (7.4)	747 (15.7)	390 (7.1)	530 (15.5)	227 (10.2)	129 (9.8)	96 (13.2)	12 (5.5)
Q9	12 986 (94.1)	8243 (92.1)	4743 (97.7)	5134 (89.1)	3401 (97.9)	2196 (96.2)	1317 (98.6)	722 (99.2)	216 (97.7)
Extremely negative	381 (2.9)	191 (2.3)	190 (4.0)	105 (2.0)	105 (3.1)	54 (2.5)	52 (4.0)	57 (7.9)	8 (3.7)
Rather negative	1226 (9.4)	685 (8.3)	541 (11.4)	470 (9.1)	268 (7.9)	162 (7.4)	237 (18.0)	69 (9.6)	20 (9.3)
Neutral	3682 (28.4)	2318 (28.1)	1364 (28.8)	1475 (28.7)	1093 (32.1)	528 (24.0)	336 (25.5)	190 (26.3)	60 (27.8)
Rather positive	5303 (40.8)	3726 (45.2)	1577 (33.2)	2292 (44.6)	1223 (36.0)	970 (44.2)	475 (36.2)	242 (33.5)	101 (46.8)
Extremely positive	2394 (18.4)	1323 (16.1)	1071 (22.6)	792 (15.4)	712 (20.9)	482 (22.0)	217 (16.5)	164 (22.7)	27 (12.5)
Q10	12 953 (93.8)	8219 (91.8)	4734 (97.5)	5110 (88.7)	3399 (97.9)	2192 (96.0)	1314 (98.4)	721 (99.0)	217 (98.2)
Extremely negative	567 (4.4)	323 (3.6)	244 (5.2)	211 (4.1)	118 (3.5)	79 (3.6)	84 (6.4)	63 (8.7)	12 (5.5)
Rather negative	1581 (12.1)	868 (10.6)	713 (15.1)	597 (11.7)	433 (12.7)	194 (8.9)	248 (18.9)	71 (9.8)	38 (17.5)
Neutral	3732 (28.1)	2354 (28.6)	1378 (29.1)	1477 (28.9)	1037 (30.5)	584 (26.6)	351 (26.7)	222 (30.8)	61 (28.1)
Rather positive	4909 (37.9)	3431 (41.7)	1478 (31.2)	2091 (40.2)	1190 (35.0)	895 (40.8)	438 (33.3)	219 (30.4)	76 (35.0)
Extremely positive	2164 (16.7)	1243 (15.1)	921 (19.5)	734 (14.4)	621 (18.3)	440 (20.1)	193 (14.7)	146 (20.3)	30 (13.8)
Q11	12 961 (93.9)	8232 (92.0)	4729 (97.4)	5122 (89.9)	3395 (97.8)	2188 (95.8)	1316 (98.5)	723 (99.3)	217 (98.2)
Extremely negative	345 (2.7)	161 (2.0)	184 (3.9)	82 (1.6)	110 (3.2)	50 (2.3)	43 (3.3)	51 (7.1)	9 (4.2)
Rather negative	836 (6.5)	392 (4.8)	444 (9.4)	255 (5.0)	280 (8.2)	79 (3.6)	112 (8.5)	93 (12.9)	17 (7.8)
Neutral	2967 (22.9)	1716 (20.8)	1251 (26.4)	1073 (21.0)	1006 (29.6)	391 (17.9)	295 (22.4)	161 (22.3)	41 (18.9)
Rather positive	5381 (41.5)	3672 (44.6)	1709 (36.1)	2238 (43.7)	1246 (36.7)	976 (44.6)	594 (45.1)	232 (32.1)	95 (43.8)
Extremely positive	3432 (26.5)	2291 (27.8)	1141 (24.1)	1474 (28.8)	753 (22.2)	692 (31.6)	272 (20.7)	186 (25.7)	55 (25.4)
Q12	12 563 (91.0)	7931 (88.6)	4632 (95.4)	4887 (84.9)	3331 (95.9)	2123 (93.0)	1294 (96.9)	721 (99.0)	207 (93.7)
A high degree of accuracy, the decision path is clearly comprehensible (explainable AI)	8816 (70.2)	5599 (70.6)	3217 (69.4)	3304 (67.6)	2091 (62.8)	1704 (80.3)	1045 (80.8)	520 (72.1)	152 (73.4)
A higher accuracy, the decision path however, is not comprehensible	3747 (29.8)	2332 (29.4)	1415 (30.6)	1583 (32.4)	1240 (37.2)	419 (19.7)	249 (19.2)	201 (27.9)	55 (26.6)
Q13	12 268 (88.9)	7671 (85.7)	4597 (94.7)	4680 (81.2)	3301 (95.1)	2086 (91.3)	1285 (96.2)	710 (97.5)	206 (93.2)
The AI misses almost no diagnosis, but often gives a false alarm	5701 (46.5)	3725 (48.6)	1976 (43.0)	2224 (47.5)	1323 (40.1)	1025 (49.1)	673 (52.4)	336 (47.3)	120 (58.2)
The AI almost never gives a false alarm, but sometimes misses a diagnosis	4452 (36.3)	2663 (34.7)	1789 (38.9)	1668 (35.6)	1296 (39.3)	732 (35.1)	456 (35.5)	243 (34.2)	57 (27.7)
The AI gives a false alarm about as often as it misses a diagnosis	2115 (17.2)	1283 (16.7)	832 (18.1)	788 (16.8)	682 (20.7)	329 (15.8)	156 (12.1)	131 (18.5)	29 (14.1)
Q14	12 652 (91.6)	8089 (90.4)	4563 (94.0)	5012 (87.0)	3305 (95.2)	2157 (94.4)	1244 (93.1)	720 (98.9)	214 (96.8)
Physicians make the diagnosis alone	829 (6.6)	570 (7.1)	259 (5.7)	345 (6.9)	198 (6.0)	111 (5.2)	113 (9.1)	51 (7.1)	11 (5.1)
AI and physicians make the diagnosis together. Physicians make the final decision	9222 (72.9)	6092 (75.3)	3130 (68.6)	3853 (76.9)	2212 (66.9)	1594 (73.9)	896 (72.0)	496 (68.9)	171 (79.9)
AI and physicians make the diagnosis together. Both have equal authority	1722 (13.6)	1063 (13.1)	659 (14.4)	574 (11.4)	563 (17.0)	298 (13.8)	152 (12.2)	114 (15.8)	21 (9.8)
AI and physicians make the diagnosis together. AI makes the final decision	317 (2.5)	153 (1.9)	164 (3.6)	111 (2.2)	98 (3.0)	30 (1.4)	40 (3.2)	34 (4.7)	4 (1.9)
AI makes the diagnosis alone	562 (4.4)	211 (2.6)	351 (7.7)	129 (2.6)	234 (7.1)	124 (5.8)	43 (3.5)	25 (3.5)	7 (3.3)
Q15	12 652 (91.6)	8077 (90.2)	4575 (94.2)	4992 (86.6)	3244 (93.4)	2167 (94.9)	1312 (98.2)	723 (99.3)	214 (96.8)
Extremely negative	322 (2.6)	138 (1.7)	184 (4.0)	69 (1.4)	93 (2.9)	39 (1.8)	38 (2.9)	76 (10.5)	7 (3.3)
Rather negative	895 (7.1)	419 (5.2)	476 (10.4)	274 (5.5)	338 (10.4)	89 (4.1)	103 (7.8)	75 (10.4)	16 (7.5)
Neutral	3594 (28.4)	2381 (29.5)	1213 (26.5)	1555 (31.2)	890 (27.4)	502 (23.2)	434 (33.1)	159 (22.0)	54 (25.2)
Rather positive	5228 (41.3)	3617 (44.8)	1611 (35.2)	2184 (43.8)	1219 (37.6)	990 (45.7)	494 (37.6)	242 (33.5)	99 (46.3)
Extremely positive	2613 (20.6)	1522 (18.8)	1091 (23.9)	910 (18.2)	704 (21.7)	547 (25.2)	243 (18.5)	171 (23.6)	38 (17.8)
Q16	12 497 (90.5)	7947 (88.8)	4523 (93.2)	4905 (85.1)	3214 (92.5)	2150 (94.1)	1298 (97.2)	715 (98.2)	215 (97.3)
Always prefer facilities without AI	775 (6.2)	426 (5.4)	349 (7.7)	299 (6.1)	213 (6.6)	78 (3.6)	130 (10.0)	38 (5.3)	17 (7.9)
Tend to prefer facilities without AI	2800 (22.4)	1855 (23.3)	945 (20.9)	1183 (24.1)	645 (20.1)	426 (19.8)	355 (27.4)	126 (17.6)	65 (30.2)
Rather prefer facilities with AI	7022 (56.2)	4750 (59.8)	2272 (50.2)	2841 (57.9)	1808 (56.2)	1287 (59.9)	656 (50.5)	314 (43.9)	116 (54.0)
Always prefer facilities with AI	1900 (15.2)	943 (11.9)	957 (21.2)	582 (11.9)	548 (17.0)	359 (16.7)	157 (12.1)	237 (33.2)	17 (7.9)
Q17	12 668 (91.8)	8104 (90.5)	4564 (94.0)	5019 (87.1)	3239 (93.3)	2161 (94.6)	1311 (98.1)	722 (99.2)	216 (97.7)
Very worried	2324 (18.4)	1497 (18.5)	827 (18.1)	718 (14.3)	441 (13.6)	703 (32.5)	267 (20.4)	152 (21.1)	43 (19.9)
Somewhat concerned	4413 (34.8)	2830 (34.9)	1583 (34.7)	1673 (33.3)	1233 (38.1)	727 (33.6)	441 (33.6)	252 (34.9)	87 (40.3)
Neutral	3595 (28.4)	2220 (27.4)	1375 (30.1)	1513 (30.2)	1008 (31.1)	453 (21.0)	350 (26.7)	213 (29.5)	58 (26.8)
Rather unconcerned	1627 (12.8)	1115 (13.8)	512 (11.2)	775 (15.4)	394 (12.2)	219 (10.1)	151 (11.5)	67 (9.3)	21 (9.7)
Unconcerned	709 (5.6)	442 (5.4)	267 (5.8)	340 (6.8)	163 (5.0)	59 (2.7)	102 (7.8)	38 (5.3)	7 (3.2)
Q18	12 669 (91.8)	8109 (90.6)	4560 (93.9)	5025 (87.2)	3237 (93.2)	2160 (94.6)	1312 (98.2)	719 (98.8)	216 (97.7)
Very worried	3123 (24.6)	2150 (26.5)	973 (21.3)	1206 (24.0)	516 (15.9)	786 (36.4)	385 (29.3)	177 (24.6)	53 (24.5)
Somewhat concerned	4700 (37.1)	3174 (39.1)	1526 (33.5)	2023 (40.3)	1265 (39.1)	698 (32.3)	375 (28.6)	241 (33.5)	98 (45.4)
Neutral	2958 (23.4)	1699 (21.0)	1259 (27.6)	1044 (20.8)	882 (27.2)	467 (21.6)	338 (25.8)	183 (25.4)	44 (20.4)
Rather unconcerned	1399 (11.0)	862 (10.6)	537 (11.8)	593 (11.8)	403 (12.4)	178 (8.2)	133 (10.1)	76 (10.6)	16 (7.4)
Unconcerned	489 (3.9)	224 (2.8)	265 (5.8)	159 (3.2)	171 (5.3)	31 (1.4)	81 (6.2)	42 (5.8)	5 (2.3)
Q19	12 773 (92.5)	8105 (90.6)	4668 (96.2)	5025 (87.2)	3341 (96.2)	2158 (94.5)	1311 (98.1)	722 (99.2)	216 (97.7)
Very worried	3868 (30.3)	2724 (33.6)	1144 (24.5)	1580 (31.4)	593 (17.8)	918 (42.5)	466 (35.6)	230 (31.9)	81 (37.5)
Somewhat concerned	4018 (31.5)	2743 (33.8)	1275 (27.3)	1717 (34.2)	1102 (33.0)	647 (30.0)	293 (22.4)	187 (25.9)	72 (33.3)
Neutral	2595 (20.2)	1409 (17.4)	1186 (25.4)	841 (16.7)	867 (26.0)	375 (17.4)	334 (25.5)	143 (19.8)	35 (16.2)
Rather unconcerned	1571 (12.3)	910 (11.2)	661 (14.2)	647 (12.9)	501 (15.0)	180 (8.3)	131 (10.0)	89 (12.3)	23 (10.6)
Unconcerned	721 (5.6)	319 (3.9)	402 (8.6)	240 (4.8)	278 (8.2)	38 (1.8)	87 (6.6)	73 (10.1)	5 (2.3)
Q20	12 751 (92.4)	8091 (90.4)	4660 (96.0)	5009 (86.9)	3334 (96.0)	2159 (94.5)	1310 (98.1)	723 (99.3)	216 (97.7)
Very worried	3330 (26.1)	2151 (26.6)	1179 (25.3)	1093 (21.8)	676 (20.3)	845 (39.1)	421 (32.1)	245 (33.9)	50 (23.2)
Somewhat concerned	3989 (31.3)	2439 (30.1)	1550 (33.3)	1408 (28.1)	1241 (37.2)	688 (31.9)	362 (27.6)	215 (29.7)	75 (34.7)
Neutral	3356 (26.3)	2205 (27.2)	1151 (24.7)	1518 (30.3)	872 (26.2)	444 (20.6)	327 (25.0)	132 (18.3)	63 (29.2)
Rather unconcerned	1365 (10.7)	885 (10.9)	480 (10.3)	683 (13.6)	359 (10.8)	128 (5.9)	127 (9.7)	51 (7.0)	17 (7.9)
Unconcerned	711 (5.6)	411 (5.1)	300 (6.4)	307 (6.1)	186 (5.6)	54 (2.5)	73 (5.6)	80 (11.1)	11 (5.1)

Abbreviations: AI, artificial intelligence; Q, question.

^a^ All percentages are calculated relative to the number of respondents per question within each geographic region rather than the total patient population.

^b^ Q1, What are your general views on the use of AI in medicine? Q2, AI should be increasingly used in the health care sector. Q3, How much confidence do you have that AI can improve health care? Q4, How much do you trust an AI to provide reliable information about your health? Q5, How much do you trust an AI to provide accurate information about your diagnosis? Q6, How much do you trust an AI to provide accurate information about your response to therapy? Q7, Which of the following statements about a potential application of AI in medicine do you most likely agree with? Q8, I would trust a highly accurate AI to make a vital decision for me. Q9, How would you rate it if a certified AI software analyzes x-ray images? Q10, How would you rate it if a certified AI software diagnoses cancer? Q11, How would you rate it if a certified AI software would be available to doctors as a second opinion? Q12/Q13, Suppose an AI makes a diagnosis. What would you prefer? Q14, Suppose an AI has about the same accuracy as doctors. Which situation for a diagnosis would you prefer? Q15, What do you think of health care facilities (clinics, practices) using AI software to aid in diagnosis? Q16, Would you prefer to visit health care facilities that use AI software? Q17, How concerned are you about AI compromising the protection of your personal data? Q18, How concerned are you that the use of AI will reduce the contact between physicians and patients? Q19, How concerned are you that AI could replace human doctors in the future? Q20, How concerned are you that the use of AI will lead to higher health care costs?

### General Attitudes Toward AI

Most patients were positive about the general use of AI in medicine in question 1 (Q1) (n = 7775 of 13 502 [57.6%]) and favored its increasing application in health care in Q2 (n = 838 of 13 314 [62.9%]). Female respondents were slightly less positive about the general use of AI in medicine than males, with 55.6% of female respondents (n = 3511 of 6318) having rather positive or extremely positive views on AI compared with 59.1% of male respondents (n = 4057 of 6864) (adjusted odds ratio [AOR], 0.84 [95% CI, 0.78-0.90] for Q1) (eTable 6 in Supplement 1). Patients were more dismissive toward AI if they reported worse overall health status. Of patients with very poor health status, 26.6% (n = 53 of 199) had extremely negative views on AI and 29.2% (n = 58 of 199) had rather negative views on AI. In comparison, only 1.3% (n = 33 of 2538 with extremely negative views) and 5.3% (n = 134 of 2538 with rather negative views) of patients with very good health shared those views. This pattern was also reflected in the AORs, which increased with higher self-reported health status, ranging from 0.15 (95% CI, 0.11-0.21) for very poor health to 0.71 (95% CI, 0.64-0.78) for good health, compared with respondents who indicated very good health (Q1) (eTable 7 in Supplement 1). Gantt diagrams depicting the response distribution for survey items Q1 and Q2 are provided in eFigure 1 in Supplement 1.

Similar observations were made for higher AI knowledge, in which 83.3% (n = 175 of 210) of self-reported AI experts had rather positive or extremely positive views, compared with 38.0% (n = 667 of 1755) of respondents with no AI knowledge (Q1). For this question, AORs ranged from 1.75 (95% CI, 1.56-1.96) for little AI knowledge to 7.11 (95% CI, 5.19-9.74) for expert AI knowledge compared with no AI knowledge (eTable 8 in Supplement 1).

Patients with a higher technological literacy, measured by the number of technology devices used weekly, also expressed more positive views on AI (AOR, 1.17 [95% CI, 1.13-1.21] for Q1) (eTable 9 in Supplement 1). Age (AOR, 0.99 [95% CI, 0.78-1.26] for Q1; AOR, 1.07 [95% CI, 0.84-1.36] for Q2) and level of education (eg, AOR, 1.27 [95% CI, 1.1-1.48] for Q1 and 1.05 [95% CI, 0.91-1.2] for Q2 comparing elementary school with university education) were not significantly associated with general attitudes (eTables 9 and 10 in Supplement 1). Absolute survey results for each item stratified by gender, education level, health status, AI knowledge, age, and weekly use of technological devices are presented in eTables 11 through 15 in Supplement 1.

### Trust in AI

Less than half of the respondents indicated a positive attitude toward the items related to trust in AI. Overall, 48.5% (n = 6573 of 13 542) of patients surveyed were confident that AI would improve health care (Q3), 43.9% (n = 5935 of 13 507) trusted AI to provide reliable health information (Q4), 43.6% (n = 5887 of 13 496) trusted AI to provide accurate information about their diagnosis (Q5), and 41.8% (n = 5637 of 13 480) trusted AI to provide accurate information about their response to therapy (Q6).

While the majority of female patients responded positively toward AI for all items on trust, they were slightly less favorable than male patients, reflected in the lower AORs compared with males, ranging from 0.76 (95% CI, 0.71-0.80 for Q5 and 95% CI, 0.71-0.81 for Q6) to 0.80 (95% CI, 0.74-0.85 for Q4) (eTable 6 in Supplement 1). For instance, in Q3, 45.0% of female respondents (n = 2862 of 6357) had much confidence or very much confidence that AI can improve health care, compared with 51.4% of male respondents (n = 3526 of 6861) (eTable 11 in Supplement 1).

**Figure 2.**
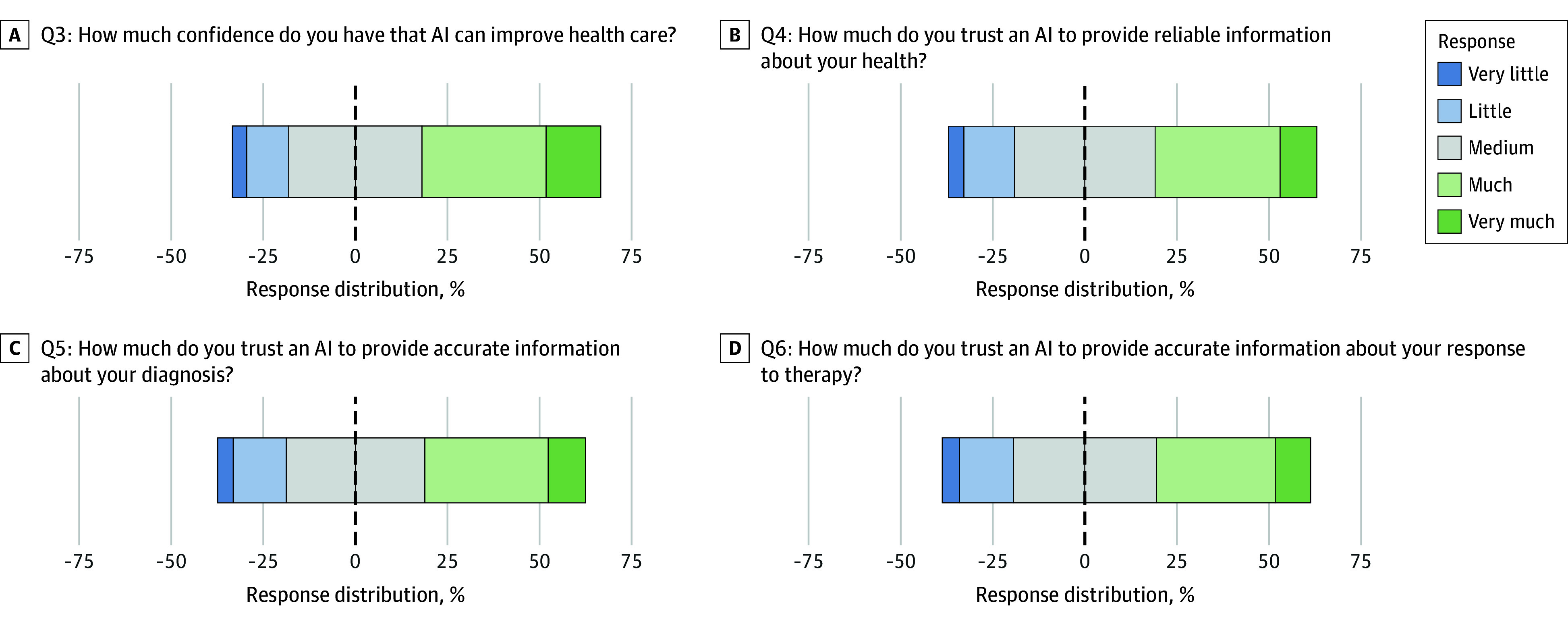
Gantt Diagrams Depicting the Results for Each Item in the Trust in Artificial Intelligence (AI) Section Q3 through Q6 represent question items 3 through 6.

For Q3 to Q6, patients with expert knowledge consistently answered more favorably toward AI, with AORs ranging from 3.26 (95% CI, 2.41-4.41) in Q4 to 5.11 (95% CI, 3.76-6.94) in Q3 (eTable 8 in Supplement 1). For example, in Q3, 77.3% of self-reported AI experts (n = 163 of 211) had much confidence or very much confidence in AI improving health care, compared with only 35.9% of respondents (n = 630 of 1756) with no AI knowledge (eTable 13 in Supplement 1). Similarly, AORs for Q3 to Q6 increased with better self-reported health status, with the reference group of patients reporting very good health consistently demonstrating the highest AORs (eTable 7 in Supplement 1). Gantt diagrams depicting the response distribution for all items on trust in AI are shown in Figure 2.

Only 11.4% of patients (n = 1499 of 13 139) were against using AI regardless of the disease (Q7). In contrast, of 13 139 patients, 27.9% (n = 3661 of 13 139) preferred to use AI only for minor conditions, such as the common cold, 26.7% (n = 3510 of 13 139) accepted AI for moderate conditions, such as appendicitis, and 34.0% (n = 4469 of 13 139) were open to using AI for severe conditions, such as traffic accidents. Notably, 42.9% of patients (n = 5758 of 13 437) trusted a highly accurate AI to make vital health decisions on their behalf (Q8). However, this trust varied significantly in terms of both health status and AI knowledge. Of patients with very good health, 50.4% (n = 1273 of 2526) trusted AI for vital decisions, compared with only 26.0% of patients (n = 51 of 196) with very poor health, reflected in an AOR of 0.45 (95% CI, 0.33-0.62) (eTables 7 and 12 in Supplement 1). Of self-reported AI experts, 91.4% (n = 192 of 210) trusted or strongly trusted AI for vital decisions, compared with only 36.0% (n = 628 of 1745) of respondents with no AI knowledge, with AORs ranging from 1.18 (95% CI, 1.06-1.32) for little knowledge to 2.06 (95% CI, 1.52-2.8) for expert knowledge (eTables 8 and 13 in Supplement 1).

### Preferences Toward AI Applications in Diagnostics and Health Care Facilities

Most patients preferred health care facilities that use AI software to assist in diagnosis (Q15), with 62.0% (n = 7841 of 12 652) expressing a positive attitude. Similarly, most patients indicated that they would often or always prefer facilities that use AI (n = 8922 of 12 497 [71.4%] for Q16). Female patients were less positive than male patients for both items (n = 3585 of 5990 [59.9%] vs n = 4041 of 6329 [63.9%] for Q15; n = 4061 of 5912 [68.7%] vs n = 4618 of 6263 [73.7%] for Q16), with AORs of 0.79 (95% CI, 0.78-0.79) for Q15 and 0.81 (95% CI, 0.75-0.88) for Q16 (eTables 6 and 11 in Supplement 1). AI knowledge was also significantly associated with these preferences, with AORs for expert knowledge of 4.77 (95% CI, 3.51-6.48) for Q15 and 3.18 (95% CI, 2.29-4.42) for Q16, compared with no knowledge. For instance in Q15, 79.7% of self-reported AI experts (n = 161 of 202) had positive attitudes toward health care facilities using AI compared with 46.2% of respondents with no knowledge (n = 747 of 1616) (eTables 8 and 13 in Supplement 1). Younger age and higher technological literacy were only associated with a more positive attitude for Q15 (eTable 9 in Supplement 1).

The use of AI was viewed positively in various medical scenarios: 59.3% (n = 7697 of 12 986) supported the use of AI for radiograph analysis (Q9), 54.6% (n = 7073 of 12 953) for cancer diagnosis (Q10), and 67.9% (n = 8804 of 12 961) for availability as a second opinion for physicians (Q11). Notably, 70.2% of patients (n = 8816 of 12 563) preferred explainable AI (Q12), even if this meant a trade-off in accuracy compared with black-box models. This observation was consistent across subgroups with mostly nonsignificant differences.

Regarding diagnostic accuracy (Q13), 46.5% of patients (n = 5701 of 12 268) preferred AI with higher sensitivity compared with 36.3% of patients (n = 4452 of 12 268) who preferred AI with higher specificity. When asked about joint diagnosis by physicians and AI when both have the same accuracy (Q14), the majority of patients (n = 9222 of 12 652 [72.9%]) preferred a collaborative diagnostic approach in which physicians make the final decision. Only a small proportion (4.4%; n = 562 of 12 652) supported the idea of fully autonomous AI in diagnosis, while 6.6% (n = 829 of 12 652) favored physicians making the diagnosis independently of AI. Gantt diagrams depicting the response distribution for all items on preferences toward AI applications in diagnostics and health care facilities are provided in eFigure 2 in Supplement 1.

### Concerns Toward AI

Concerns about data protection were expressed in Q17 by 53.2% of patients (n = 6737 of 12 668). Even more participants were concerned about the potential influence of AI on health care delivery: 61.8% (n = 7823 of 12 669) feared that AI could reduce physician-patient interaction (Q18), while 61.7% (n = 7886 of 12 773) were concerned that AI could replace human physicians (Q19). The expectation that AI will lead to increased health care costs (Q20) was a concern for 57.4% of patients (n = 7319 of 12 751).

Younger age was associated with lower concerns for all items in Q17 to Q20, with AORs ranging from 0.36 (95% CI, 0.29-0.46) on Q18 to 0.62 (95% CI, 0.49-0.79) on Q17 (eTable 9 in Supplement 1). However, absolute differences were small, with 53.3% of patients 48 years of age or younger (n = 3167 of 5938) vs 53.4% of patients older than 48 years (n = 2984 of 5593) expressing concerns in Q17 or 57.4% of patients 48 years or younger (n = 3412 of 5940) vs 66.7% of patients older than 48 years (n = 3731 of 5592) expressing concerns in Q18 (eTable 14 in Supplement 1).

**Figure 3.**
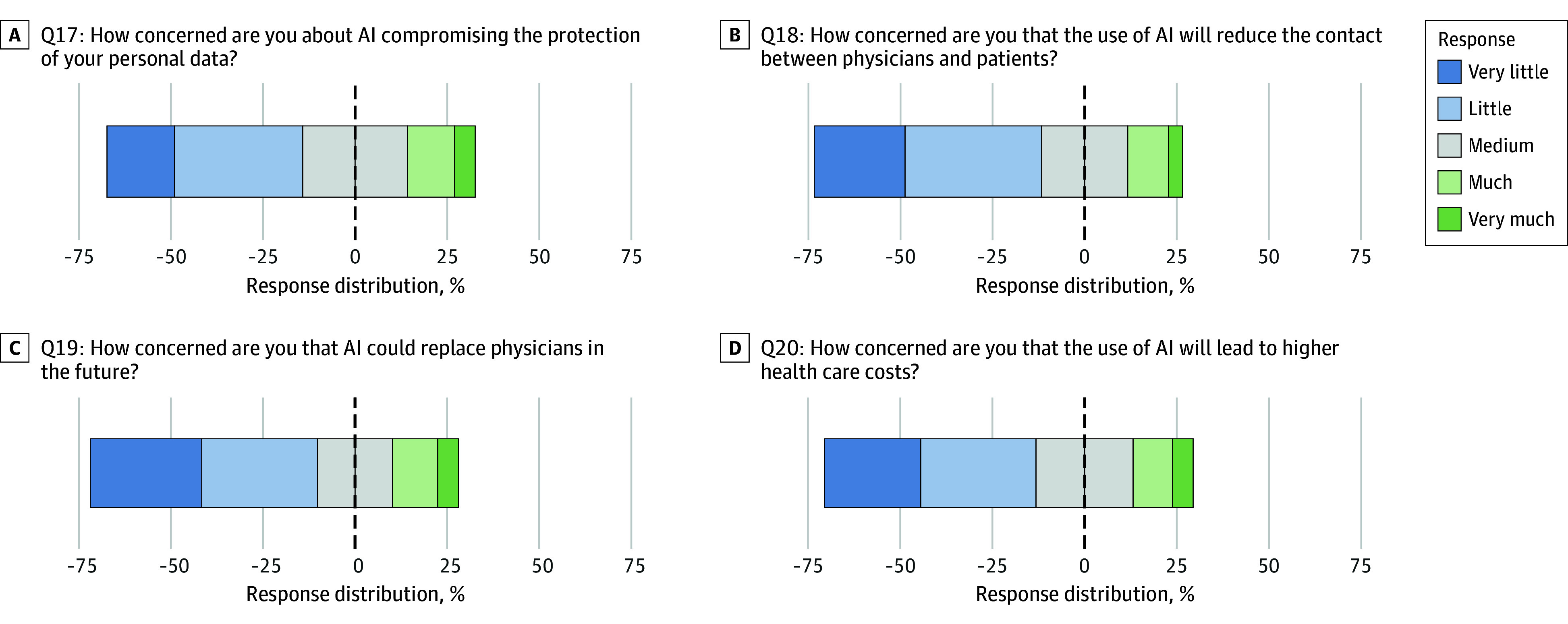
Gantt Diagrams Depicting the Results for Each Item in the Concerns Toward AI Section Q17 through Q20 represent question items 17 through 20. AI indicates artificial intelligence.

Notably, higher self-reported AI knowledge was associated with lower concerns about the replacement of human physicians in Q19 (n = 107 of 205 experts [52.2%] vs n = 1037 of 1668 [62.2%] with no knowledge; expert AOR, 1.84 [95% CI, 1.35-2.50]) and increased health care costs in Q20 (51.7% of experts vs 62.6% with no knowledge; expert AOR, 1.83 [95% CI, 1.34-2.50]) (eTables 8 and 13 in Supplement 1). Figure 3 shows Gantt diagrams depicting the responses of all items on the concern toward AI scale.

## Discussion

This multinational cross-sectional study represents, to our knowledge, the most extensive and comprehensive survey to date of patient attitudes toward AI in health care worldwide. With 13 806 participants from 43 countries, our findings provide a multifaceted understanding of patients’ preferences, trust, and concerns about AI in health care. The results illustrated a nuanced landscape of attitudes, with most patients expressing support for the use of AI in health care while also articulating concerns about its implementation.

Previous studies examining patient attitudes toward AI in health care have been limited to individual countries or specific clinical areas. For example, positive attitudes toward the use of AI in health care ranged from 53% in a German tertiary referral hospital to 94% in a German radiology patient study.^19,22,24,32^ Although this overall pattern is also reflected in our findings, with 57.6% of respondents expressing a generally positive view of the use of AI in health care, our study provides a more comprehensive and granular understanding of patient attitudes. Notably, 71.4% of patients indicated a preference for health care facilities that use AI software (Q16). Interestingly, the preference for facilities using AI was higher than the percentage of patients expressing a generally positive view of AI (57.6%, Q1) or favoring an increase in AI use in health care (62.9%, Q2). One potential explanation for this discrepancy may be that the use of AI is perceived as a marker of modern technology, and patients anticipate that other aspects of the hospital may also be more modern. While our current study design did not assess this hypothesis, future research could explore these differences in more depth through qualitative interviews or targeted surveys that examine how patients weigh technological capabilities against other institutional factors when choosing health care institutions.

Our study also observed how attitudes toward AI varied significantly based on demographic factors and health status. Young, healthy males viewed AI most positively, whereas older patients and those with poorer health expressed more reservations. This gradient of acceptance suggests that as the likelihood of AI being applied to one’s own care increases, patients become more cautious in their outlook. Similar patterns were also observed for the use of AI depending on disease severity, with patients less likely to accept AI for more severe conditions. This finding is also supported by a recent study by Khullar et al,^22^ who found that 31% of respondents to an online survey agreed with the use of AI in cancer diagnosis, compared with 55% for diagnoses based on chest radiographs. However, it is important to note that their study allowed respondents to agree with AI use in multiple scenarios and focused on AI potentially replacing physicians in these activities. In contrast, our study examined the acceptance of AI application across a spectrum of disease severities, regardless of whether it was supplementing or replacing human physicians. In contrast, a study by Robertson et al^18^ involving 2675 patients showed no significant preference for AI use based on disease severity.

While the present study findings may highlight the role of health status in shaping AI perceptions, it is important to consider that this association may be partially confounded by other psychological and experiential factors. Our study indicates that patients with more severe illnesses often exhibit greater skepticism toward AI in health care. This association may be influenced by several factors. First, individuals with chronic diseases are at higher risk of developing depression, which can lead to more severe symptoms and a general pessimistic outlook.^33^ This mental state may contribute to reduced trust in new medical technologies. Second, patients with chronic conditions typically have more frequent interactions with health care systems, increasing their exposure to potential negative experiences, such as misdiagnoses or prolonged treatments.^34^ Such experiences may diminish trust in health care innovations.

Another noteworthy finding of our study is the pronounced inclination toward explainable AI, which was observed to be independent of demographic characteristics. A total of 70.2% of patients indicated a preference for AI with transparent decision-making processes, even if this entailed a slight compromise in accuracy. This preference for explainability is considerably higher than that reported in a previous US study, which found that only 42% of patients felt uncomfortable with highly accurate AI diagnoses that lacked explainability.^22^ Our study results suggested a global desire for transparency in AI-driven health care decisions, which has important implications for the development and implementation of AI in medical settings.

Moreover, our results indicated a reinforcement in the importance of maintaining human oversight in AI-assisted health care. Despite the ongoing debate about the use of autonomous AI in health care,^35,36,37^ our findings indicate that the majority of patients prefer physicians to retain control when using AI in clinical settings. Notably, only 4.4% of patients preferred fully autonomous AI. Supporting these findings, previous studies have reported that 67% to 96% of patients would prefer physician-led diagnoses to AI recommendations.^19,24,38,39^ On the other hand, the preference for physicians to make diagnoses without the assistance of AI was expressed by only 6.6% of patients in our study, suggesting a substantial endorsement of AI among the survey participants.

Despite the generally favorable views on AI, we also observed multiple concerns among respondents. More than half of patients indicated apprehensions about data security, reduced physician-patient interaction, and potential increases in health care costs. These findings underscore the need for a balanced approach to AI implementation in health care, one that addresses patient concerns while leveraging the potential benefits of AI technology.

### Limitations

This study had limitations. A potential limitation of our study is that patient attitudes toward AI in health care may be influenced by their general perceptions of health care services. For instance, a study by de Veer et al^40^ involving 1014 older adults found that positive experiences with e-health applications were associated with higher satisfaction with health care services. Similarly, a systematic review by Rahimi et al^41^ highlighted that the successful adoption of e-health technologies is associated with patients’ overall trust and satisfaction with the health care system. These findings suggest that general attitudes toward health care may be associated with the acceptance of new technologies, including AI. Thus, there is a risk that our survey responses reflect more general sentiments about health care rather than specific views about AI, which should be further explored in a more experimental setting in future research.

Additionally, the nonprobability convenience sampling likely resulted in low response rates and may have introduced selection and noncoverage bias, affecting data representativeness. Despite these issues, the sampling method enabled the collection of diverse patient attitudes across various countries and health care settings. The uncertain selection probabilities and unsupervised survey administration may limit the robustness of inferences. To address site-specific clustering and stratification variations, we used mixed models for subgroup analysis. While substantial site-level heterogeneity (intraclass correlation = 0.22) precluded meaningful country-level comparisons, the findings offered valuable insights into multinational patient attitudes toward AI in health care and can inform future research. Additionally, while we found associations between factors such as AI knowledge and positive attitudes, our cross-sectional design could not establish whether these associations are causal or due to self-selection. Ultimately, our focus on radiology departments, while providing access to patients from diverse clinical services, introduces additional considerations. Radiology departments typically engage with more ambulatory and stable patients, and these patients may have heightened awareness of diagnostic technology given their immediate interaction with imaging services. Furthermore, the mix of referring clinical services varied substantially between institutions, potentially affecting the generalizability of our findings to other clinical settings. Importantly, while our mixed-effects models provided robust inference about associations within our study cohort, these findings should not be interpreted as representative of the general population, as our sample specifically reflected the perspectives of hospital patients who were willing and able to complete our survey. Future research should aim to further refine the understanding of how health care settings influence attitudes toward AI, including comparing hospitalized patients and outpatients. We encourage replication and extension, particularly in underrepresented populations, and have made study materials available to support this goal.

## Conclusions

In this cross-sectional study of patient attitudes toward AI in health care across 6 continents, we found that while patients generally favored AI-equipped health care facilities, they preferred explainable AI systems and physician-led decision-making. In addition, patient attitudes varied significantly based on demographics and health status. These findings may be used by stakeholders in the health care AI sector to maximize patient acceptance of AI by prioritizing transparency, maintaining human oversight, and tailoring AI implementation to patient characteristics.

## Notes

Supplement 1. **eTable 1.** STROBE Statement **eTable 2.** Institutional Review Board Approval Information **eTable 3.** Baseline Characteristics of the Pilot Study Group **eTable 4.** Baseline Characteristics of Excluded Patients **eTable 5.** Overview of Participating Institutions, Departments, and Patients **eTable 6.** Adjusted Odds Ratios for Self-Reported Gender From Regression Analyses **eTable 7.** Adjusted Odds Ratios for Self-Reported Health Status From Regression Analyses **eTable 8.** Adjusted Odds Ratios for Self-Reported AI Knowledge From Regression Analyses **eTable 9.** Adjusted Odds Ratios for Age and Number of Technical Devices Used Weekly From Regression Analyses **eTable 10.** Adjusted Odds Ratios for Highest Educational Level From Regression Analyses **eTable 11.** Absolute Survey Results for Each Item Stratified By Gender **eTable 12.** Absolute Survey Results for Each Item Stratified by Self-Reported Health Status **eTable 13.** Absolute Survey Results for Each Item Stratified by Self-Reported AI Knowledge **eTable 14.** Absolute Survey Results for Each Item Stratified by Median Age and Median Number of Technical Devices Used Weekly **eTable 15.** Absolute Survey Results for Each Item Stratified by Highest Educational Level **eFigure 1.** Gantt Diagrams Depicting the Results for Each Item in the “General Attitudes Towards AI” Section **eFigure 2.** Gantt Diagrams Depicting the Results for Each Item in the “Preferences Towards AI Applications in Diagnostics and Healthcare Facilities” Section

Supplement 2.
Data Sharing Statement
