# Supplementary material for: Multinational Attitudes Toward AI in Health Care and Diagnostics Among Hospital Patients
Source: JAMA Netw Open. 2025 Jun 10;8(6):e2514452. doi: 10.1001/jamanetworkopen.2025.14452 (PMC12152705; doi:10.1001/jamanetworkopen.2025.14452)
Supplement: Supplement 1. — eTable 1. STROBE Statement eTable 2. Institutional Review Board Approval Information eTable 3. Baseline Characteristics of the Pilot Study Group eTable 4. Baseline Characteristics of Excluded Patients eTable 5. Overview of Participating Institutions, Departments, and Patients eTable 6. Adjusted Odds Ratios for Self-Reported Gender From Regression Analyses eTable 7. Adjusted Odds Ratios for Self-Reported Health Status From Regression Analyses eTable 8. Adjusted Odds Ratios for Self-Reported AI Knowledge From Regression Analyses eTable 9. Adjusted Odds Ratios for Age and Number of Technical Devices Used Weekly From Regression Analyses eTable 10. Adjusted Odds Ratios for Highest Educational Level From Regression Analyses eTable 11. Absolute Survey Results for Each Item Stratified By Gender eTable 12. Absolute Survey Results for Each Item Stratified by Self-Reported Health Status eTable 13. Absolute Survey Results for Each Item Stratified by Self-Reported AI Knowledge eTable 14. Absolute Survey Results for Each Item Stratified by Median Age and Median Number of Technical Devices Used Weekly eTable 15. Absolute Survey Results for Each Item Stratified by Highest Educational Level eFigure 1. Gantt Diagrams Depicting the Results for Each Item in the “General Attitudes Towards AI” Section eFigure 2. Gantt Diagrams Depicting the Results for Each Item in the “Preferences Towards AI Applications in Diagnostics and Healthcare Facilities” Section [file jamanetwopen-e2514452-s001.pdf]

Supplementary Online Content

Busch F, Hoffmann L, Xu L, et al. Multinational attitudes toward AI in health care and diagnostics among hospital patients. *JAMA Netw Open*. 2025;8(6):e2514452. doi:10.1001/jamanetworkopen.2025.14452

eTable 1. STROBE statement
eTable 2. Institutional review board approval information
eTable 3. Baseline characteristics of the pilot study group
eTable 4. Baseline characteristics of excluded patients
eTable 5. Overview of participating institutions, departments, and patients
eTable 6. Adjusted odds ratios for self-reported gender from regression analyses
eTable 7. Adjusted odds ratios for self-reported health status from regression analyses
eTable 8. Adjusted odds ratios for self-reported AI knowledge from regression analyses
eTable 9. Adjusted odds ratios for age and number of technical devices used weekly from regression analyses
eTable 10. Adjusted odds ratios for highest educational level from regression analyses
eTable 11. Absolute survey results for each item stratified by gender
eTable 12. Absolute survey results for each item stratified by self-reported health status
eTable 13. Absolute survey results for each item stratified by self-reported AI knowledge
eTable 14. Absolute survey results for each item stratified by median age and median number of technical devices used weekly
eTable 15. Absolute survey results for each item stratified by highest educational level
eFigure 1. Gantt diagrams depicting the results for each item in the “General attitudes towards AI” section
eFigure 2. Gantt diagrams depicting the results for each item in the “Preferences towards AI applications in diagnostics and healthcare facilities” section

This supplementary material has been provided by the authors to give readers additional information about their work.

© 2025 Busch F et al. *JAMA Network Open*.

eTable 1. STROBE statement

	Item No	Recommendation	Location
**Title and abstract**	1	(*a*) Indicate the study's design with a commonly used term in the title or the abstract	Page 1
		(*b*) Provide in the abstract an informative and balanced summary of what was done and what was found	Page 5
**Introduction**			
Background/rationale	2	Explain the scientific background and rationale for the investigation being reported	Page 6
Objectives	3	State specific objectives, including any prespecified hypotheses	Page 6
**Methods**			
Study design	4	Present key elements of study design early in the paper	Pages 7-8
Setting	5	Describe the setting, locations, and relevant dates, including periods of recruitment, exposure, follow-up, and data collection	Page 7
Participants	6	(*a*) Give the eligibility criteria, and the sources and methods of selection of participants	Page 7
Variables	7	Clearly define all outcomes, exposures, predictors, potential confounders, and effect modifiers. Give diagnostic criteria, if applicable	Pages 8-9
Data sources/ measurement	8*	For each variable of interest, give sources of data and details of methods of assessment (measurement). Describe comparability of assessment methods if there is more than one group	Pages 8-9
Bias	9	Describe any efforts to address potential sources of bias	Pages 9-10
Study size	10	Explain how the study size was arrived at	Page 7
Quantitative variables	11	Explain how quantitative variables were handled in the analyses. If applicable, describe which groupings were chosen and why	Pages 8-9
Statistical methods	12	(*a*) Describe all statistical methods, including those used to control for confounding	Pages 7-9
		(*b*) Describe any methods used to examine subgroups and interactions	Pages 7-9
		(*c*) Explain how missing data were addressed	Page 9
		(*d*) If applicable, describe analytical methods taking account of sampling strategy	N/A
		(*e*) Describe any sensitivity analyses	Page 9
**Results**			
Participants	13*	(a) Report numbers of individuals at each stage of study—eg numbers potentially eligible, examined for eligibility, confirmed eligible, included in the study, completing follow-up, and analysed	Pages 9-10
		(b) Give reasons for non-participation at each stage	Pages 9-10
		(c) Consider use of a flow diagram	N/A
Descriptive data	14*	(a) Give characteristics of study participants (eg demographic, clinical, social) and information on exposures and potential confounders	Pages 9-10
		(b) Indicate number of participants with missing data for each variable of interest	Pages 9-13
Outcome data	15*	Report numbers of outcome events or summary measures	Pages 9-13
Main results	16	(*a*) Give unadjusted estimates and, if applicable, confounder-adjusted estimates and their precision (eg,	Pages 9-13

© 2025 Busch F et al. *JAMA Network Open*.

eTable 1. STROBE statement

	Item No	Recommendation	Location
		95% confidence interval). Make clear which confounders were adjusted for and why they were included	
		(*b*) Report category boundaries when continuous variables were categorised	N/A
		(*c*) If relevant, consider translating estimates of relative risk into absolute risk for a meaningful time period	N/A
Other analyses	17	Report other analyses done—eg analyses of subgroups and interactions, and sensitivity analyses	Pages 9-13
**Discussion**			
Key results	18	Summarise key results with reference to study objectives	Pages 13-15
Limitations	19	Discuss limitations of the study, taking into account sources of potential bias or imprecision. Discuss both direction and magnitude of any potential bias	Pages 15-16
Interpretation	20	Give a cautious overall interpretation of results considering objectives, limitations, multiplicity of analyses, results from similar studies, and other relevant evidence	Pages 13-15
Generalisability	21	Discuss the generalisability (external validity) of the study results	Pages 15-16
**Other information**			
Funding	22	Give the source of funding and the role of the funders for the present study and, if applicable, for the original study on which the present article is based	Pages 17-18

[*] Give information separately for exposed and unexposed groups.
Abbreviation: N/A, not applicable.

© 2025 Busch F et al. *JAMA Network Open*.

eTable 2. Institutional review board approval information

Institution	Approval number
Alfred Health	305/23
All India Institute of Medical Sciences	IEC-209/11.04.2023, OP-32/16.05.2023
A.C.Camargo Cancer Center	6.443.601 (CAEE N ° 74036923.5.0000.5432)
Aristotle University of Thessaloniki	75078/2023
ARS Algarve/Coimbra University and Medical School	36/2023
Center for Medical Education and Clinical Research (CEMIC)	9256
Centro Hospitalar Vila Nova de Gaia-Espinho	145/2023
Charité – University Medicine Berlin	EA4/213/22
Chiba University Hospital	HK202305-06
Faculty of Medicine, Chiang Mai University	RAD-2566-09468
Federal University of Rio Grande do Norte	6.274.288 (CAEE N° 69991023.4.0000.0253)
Hospital Universitario Fundación Jiménez Díaz	PIC097-23
Institute of Oncology Ljubljana	ERIDEK-0037/2023
Fundación Universitaria Sanitas - Instituto Global de Excelencia Clínica	CEIFUS 1454-23
KIST Medical College and Teaching Hospital	2079/80/94
Leiden University Medical Center	23-3015
Linnaeus University, Umeå University, Lund University	2023-03578-01
McGill University Health Center	2023-9537
Mie Chuo Medical Center	MCERB-202301
Muhammadiyah University of Palembang	113/EC/KBHKI/FK-UMP/V/2023
Ramón y Cajal University Hospital	139/23
Royal Victorian Eye and Ear Hospital	23-1580HL
Ulster University	23/0039
Universidad de Las Américas	2023-EXC-003
Universidade Federal de São Paulo	6.416.852 (CAEE N° 70812723.0.0000.5505)
University Hospital Brno	13-080323/EK
University Hospital Dubrava	2023/1602-09
Algarve University Hospital Center	UAIF 062/2023
University of Cape Town	223/2023
Universidad de Costa Rica	CEC-UCR-356-2023
University of Medicine and Pharmacy, Hue University	H2023/022
Fondazione IRCCS Policlinico San Matteo	0020473/23
University of Salerno	13/23

© 2025 Busch F et al. *JAMA Network Open*.

eTable 3. Baseline characteristics of the pilot study group

	Total (N=100)
Gender, no. (%)	-
Female	36 (36%)
Male	64 (64%)
Diverse	0
Not reported	0
Age	-
Median (IQR), y	59 (40-67)
Not reported, no. (%)	17 (17%)
Highest educational level, no. (%)	-
Elementary School Diploma	12 (12%)
Middle School Diploma	30 (30%)
High School Diploma	18 (18%)
University Degree	38 (38%)
Not reported	2 (2%)
Which of these technical devices do you use at least once a week?	-
Median (IQR), total no.	2 (2-3)
Smartphone, no. (%)	96 (96%)
PC/laptop, no. (%)	71 (71%)
Game console (eg, PlayStation, Switch), no. (%)	8 (8%)
Tablet (eg, iPad), no. (%)	33 (33%)
E-reader, no. (%)	8 (8%)
Smartwatch, no. (%)	23 (23%)
None, no. (%)	2 (2%)
Not reported, no.	0
What is your current general state of health?, no. (%)	-
Very poor	0
Poor	13 (13%)
Sufficient	47 (47%)
Good	27 (27%)
Very good	13 (13%)
Not reported	3 (3%)
How would you rate your knowledge of artificial intelligence (AI)?, no. (%)	-
No knowledge (never heard of AI)	8 (8%)
Little knowledge (eg, documentary seen on television)	64 (64%)
Good knowledge (eg, read several articles about AI)	25 (25%)
Expert (eg, involved in AI development)	2 (2%)
Not reported	1 (1%)

Abbreviation: IQR, interquartile range.

© 2025 Busch F et al. *JAMA Network Open*.

eTable 4. Baseline characteristics of excluded patients

	Total (N=149)
Institution, no. (%)	
Charité – University Medicine Berlin, Campus Benjamin Franklin	127 (85.23%)
University of Warmia and Mazury	8 (5.37%)
Faculty of Health Sciences Brandenburg, Brandenburg Medical School Theodor Fontane	3 (2.01%)
Jinling Hospital, Affiliated Hospital of Medical School, Nanjing University	3 (2.01%)
Coimbra University and Medical School	2 (1.34%)
Butabika National Referral Mental Hospital	1 (0.67%)
Dr. B.R.A. Institute Rotary Cancer Hospital, All India Institute of Medical Sciences	1 (0.67%)
Max Institute of Cancer Care, Vaishali	1 (0.67%)
Royal Victoria Hospital	1 (0.67%)
Universidad de Costa Rica	1 (0.67%)
University of Cape Town	1 (0.67%)
Gender, no. (%)	-
Female	63 (42.28%)
Male	77 (51.68%)
Diverse	0
Not reported	6 (4.03%)
Age	-
Median (IQR), y	62 (53-74)
Not reported, no. (%)	58 (38.93%)
Highest educational level, no. (%)	-
Elementary School Diploma	23 (15.44%)
Middle School Diploma	23 (15.44%)
High School Diploma	10 (6.71%)
University Degree	27 (18.12%)
Not reported	76 (51.01%)
Which of these technical devices do you use at least once a week?	-
Median (IQR), total no.	2 (1-3)
Smartphone, no. (%)	59 (39.6%)
PC/laptop, no. (%)	32 (21.48%)
Game console (eg, PlayStation, Switch), no. (%)	3 (2.01%)
Tablet (eg, iPad), no. (%)	17 (11.41%)
E-reader, no. (%)	3 (2.01%)
Smartwatch, no. (%)	10 (6.71%)
None, no. (%)	6 (4.03%)
Not reported, no. (%)	91 (61.07%)
What is your current general state of health?, no. (%)	-
Very poor	1 (0.67%)
Poor	8 (5.37%)
Sufficient	19 (12.75%)
Good	18 (12.08%)
Very good	10 (6.71%)
Not reported	102 (68.46%)

Abbreviation: IQR, interquartile range.

© 2025 Busch F et al. *JAMA Network Open*.

eTable 5. Overview of participating institutions, departments, and patients

Country/Institution	City	Department	Patients, no. (%)
Total	-	-	13806
Argentina	-	-	421 (3.05%)
1. Center for Medical Education and Clinical Research (CEMIC)	Autonomous City of Buenos Aires	Radiology	329 (2.38%)
2. Hospital Italiano de Buenos Aires	Autonomous City of Buenos Aires	Radiology	92 (0.67%)
Australia	-	-	221 (1.60%)
3. Royal Victorian Eye and Ear Hospital	Melbourne	Ophthalmology	100 (0.72%)
4. The Alfred Hospital	Melbourne	Radiology	121 (0.88%)
Austria	-	-	225 (1.63%)
5. Medical University of Vienna	Vienna	Orthopedics, Trauma Surgery	225 (1.63%)
Azerbaijan	-	-	100 (0.72%)
6. Azerbaijan Medical University	Baku	Surgery	100 (0.72%)
Brazil	-	-	581 (4.21%)
7. A.C.Camargo Cancer Center	São Paulo	Radiology	130 (0.94%)
8. Federal University of Rio Grande do Norte	Natal	Collective Health	100 (0.72%)
9. Pontifical Catholic University of Rio Grande do Sul	Porto Alegre	Radiology	100 (0.72%)
10. Universidade Federal de São Paulo	São Paulo	Radiology	251 (1.82%)
Bulgaria	-	-	255 (1.85%)
11. Karidad Medical Health Center	Plovdiv	Cardiology	255 (1.85%)
Canada	-	-	816 (5.91%)
12. Montreal General Hospital	Montreal	Radiology	299 (2.17%)
13. Royal Victoria Hospital	Montreal	Radiology	457 (3.31%)
14. Lachine Hospital	Montreal	Radiology	60 (0.43%)
China			1315 (9.52%)
15. Jinling Hospital, Affiliated Hospital of Medical School, Nanjing University	Nanjing	Radiology	1019 (7.38%)
16. Renji Hospital, Shanghai Jiao Tong University School of Medicine	Shanghai	Rheumatology	160 (1.16%)
17. Zhongda Hospital, Southeast University	Nanjing	Radiology	136 (0.99%)
Colombia	-	-	100 (0.72%)
18. Fundación Universitaria Sanitas	Bogotá D.C.	Primary care	100 (0.72%)
Costa Rica	-	-	258 (1.87%)
19. Universidad de Costa Rica	San José	Radiology	258 (1.87%)
Croatia	-	-	100 (0.72%)
20. Clinical Hospital Dubrava	Zagreb	Hematology	100 (0.72%)
Czech Republic	-	-	148 (1.07%)
21. Masaryk University	Brno	Infectious diseases	148 (1.07%)
Ecuador	-	-	234 (1.69%)
22. Universidad de Las Américas	Quito	Respiratory Medicine	234 (1.69%)
France	-	-	103 (0.75%)
23. University of Bordeaux	Bordeaux	Urology	103 (0.75%)
Germany	-	-	2091 (15.15%)
24. Charité – University Medicine Berlin, Campus Benjamin Franklin	Berlin	Radiology	1639 (11.87%)
25. Ludwig Maximilian University of Munich	Munich	Radiology	134 (0.97%)

© 2025 Busch F et al. *JAMA Network Open*.

eTable 5. Overview of participating institutions, departments, and patients

Country/Institution	City	Department	Patients, no. (%)
26. Faculty of Health Sciences Brandenburg, Brandenburg Medical School Theodor Fontane	Neuruppin	Urology	139 (1.01%)
27. Technical University of Munich	Munich	Radiology	165 (1.20%)
28. University Hospital Aachen	Aachen	Radiology	14 (0.10%)
Ghana	-	-	172 (1.25%)
29. RAAJ Specialist Scan	Cape Coast	Radiology	50 (0.36%)
30. Sonotech Medical Center	Accra	Radiology	100 (0.72%)
31. Wenchi Methodist Hospital	Wenchi	Radiology	22 (0.16%)
Greece	-	-	187 (1.35%)
32. Aristotle University of Thessaloniki	Thessaloniki	Urology	187 (1.35%)
Hungary	-	-	145 (1.05%)
33. Semmelweis University	Budapest	Otorhinolaryngology, Head and Neck Surgery	145 (1.05%)
India	-	-	300 (2.17%)
34. Dr. B.R.A. Institute Rotary Cancer Hospital, All India Institute of Medical Sciences	New Delhi	Oncology	100 (0.72%)
35. Max Institute of Cancer Care, Vaishali	Ghaziabad	Oncology	98 (0.71%)
36. VPS Lakeshore Hospital	Kochi	Gastroenterology	102 (0.74%)
Indonesia	-	-	138 (1.00%)
37. Muhammadiyah University of Palembang	Palembang	Neurology	138 (1.00%)
Italy	-	-	368 (2.67%)
38. Azienda Ospedaliero - Universitaria di Cagliari	Cagliari	Radiology	106 (0.77%)
39. University of Naples "Federico II"	Naples	Radiology	127 (0.92%)
40. University of Pavia	Pavia	Gastroenterology	135 (0.98%)
Japan	-	-	556 (4.03%)
41. Chiba University	Chiba	Cardiology	111 (0.80%)
42. Hiroshima City Hiroshima Citizens Hospital	Hiroshima	Dermatology	200 (1.45%)
43. Kyoto University Graduate School of Medicine	Kyoto	Ophthalmology	138 (1.00%)
44. Mie Chuo Medical Center	Tsu	Neurosurgery	107 (0.78%)
Mexico	-	-	1110 (8.04%)
45. National Institute of Cardiology Ignacio Chavez	Mexico City	Cardiology	249 (1.80%)
46. Salvador Zubirán National Institute of Health Sciences and Nutrition	Mexico City	Gastroenterology, Unit of Liver Transplantation	861 (6.24%)
Nepal	-	-	125 (0.91%)
47. KIST Medical College and Teaching Hospital	Kathmandu	Outpatient	125 (0.91%)
Nigeria	-	-	173 (1.25%)
48. University of Ilorin Teaching Hospital	Ilorin	Anesthesiology	173 (1.25%)
Poland	-	-	469 (3.40%)
49. Medical University of Warsaw	Warsaw	Neurology	158 (1.14%)
50. University of Warmia and Mazury	Olsztyn	Ophthalmology	163 (1.18%)
51. Wroclaw Medical University	Wroclaw	Otolaryngology, Head and Neck Surgery	148 (1.07%)
Portugal	-	-	348 (2.52%)

© 2025 Busch F et al. *JAMA Network Open*.

eTable 5. Overview of participating institutions, departments, and patients

Country/Institution	City	Department	Patients, no. (%)
52. Centro Hospitalar Vila Nova de Gaia/Espinho	Vila Nova de Gaia	Oncology	91 (0.66%)
53. Coimbra University and Medical School	Coimbra	Otorhinolaryngology, Head and Neck Surgery	143 (1.04%)
54. University of Algarve	Faro	Radiology	114 (0.83%)
Republic of Moldova	-	-	167 (1.21%)
55. Nicolae Testemițanu State University of Medicine and Pharmacy	Chișinău	Respiratory Medicine, Allergology	167 (1.21%)
Republic of North Macedonia	-	-	104 (0.75%)
56. Ss. Cyril and Methodius University	Skopje	Cardiac Surgery	104 (0.75%)
Rwanda	-	-	100 (0.72%)
57. Byumba Level 2 Teaching Hospital	Byumba City	Outpatient	100 (0.72%)
Slovenia	-	-	189 (1.37%)
58. Institute of Oncology Ljubljana	Ljubljana	Oncology	189 (1.37%)
South Africa	-	-	109 (0.79%)
59. University of Cape Town	Cape Town	Orthopedics	109 (0.79%)
Spain	-	-	228 (1.65%)
60. Hospital Universitario Fundación Jiménez Díaz	Madrid	Cardiology	128 (0.93%)
61. University Hospital Ramón y Cajal	Madrid	Radiology	100 (0.72%)
Sweden	-	-	394 (2.85%)
62. Central Hospital Växjö	Växjö	Radiology	183 (1.33%)
63. Lund University	Malmö	Radiology	144 (1.04%)
64. Umeå University	Umeå	Outpatient	67 (0.49%)
Switzerland	-	-	200 (1.45%)
65. Kantonsspital Glarus	Glarus	Radiology	200 (1.45%)
Thailand	-	-	128 (0.93%)
66. Chiang Mai University	Chiang Mai	Radiology	128 (0.93%)
The Netherlands	-	-	43 (0.31%)
67. Leiden University Medical Center	Leiden	Ophthalmology	43 (0.31%)
Tunisia	-	-	103 (0.75%)
68. Centre Hospitalier Universitaire Hédi Chaker de Sfax	Sfax	Obstetrics, Gynecology	103 (0.75%)
Turkey	-	-	284 (2.06%)
69. Hacettepe University	Ankara	Nuclear Medicine	284 (2.06%)
Uganda	-	-	71 (0.51%)
70. Butabika National Referral Mental Hospital	Kampala	Radiology	71 (0.51%)
United Arab Emirates	-	-	95 (0.69%)
71. Ajman University	Ajman	Oral surgery	95 (0.69%)
United States	-	-	100 (0.72%)
72. University of Florida	Florida	Radiology	100 (0.72%)
Vietnam	-	-	432 (3.13%)
73. Hanoi Medical University Hospital	Hanoi	Radiology	308 (2.23%)
74. Hue University	Hue	Radiology	124 (0.90%)

Abbreviation: NA, not available.

© 2025 Busch F et al. *JAMA Network Open*.

eTable 6. Adjusted odds ratios for self-reported gender from regression analyses

	**Male (N=6973)**	**Female (N=6451)**	**Diverse (N=32)**
Q1[a], no. (%)	6099 (87.47%)	5775 (89.52%)	24 (75%)
Adjusted OR (95% CI), *P* value	1 [Reference]	0.84 (0.78-0.9), p<.001	0.54 (0.24-1.22), p=1
Q2[a], no. (%)	5970 (85.62%)	5724 (88.73%)	23 (71.88%)
Adjusted OR (95% CI), *P* value	1 [Reference]	0.75 (0.7-0.8), p<.001	0.49 (0.2-0.78), p=1
Q3[a], no. (%)	6079 (87.18%)	5767 (89.4%)	24 (75%)
Adjusted OR (95% CI), *P* value	1 [Reference]	0.77 (0.71-0.82), p<.001	0.56 (0.24-1.3), p=1
Q4[a], no. (%)	6072 (87.08%)	5755 (89.21%)	24 (75%)
Adjusted OR (95% CI), *P* value	1 [Reference]	0.8 (0.74-0.85), p<.001	0.36 (0.16-0.79), p=1
Q5[a], no. (%)	6069 (87.04%)	5758 (89.26%)	24 (75%)
Adjusted OR (95% CI), *P* value	1 [Reference]	0.76 (0.71-0.8), p<.001	0.42 (0.2-0.90), p=1
Q6 [a], no. (%)	6062 (86.94%)	5753 (89.18%)	24 (75%)
Adjusted OR (95% CI), *P* value	1 [Reference]	0.76 (0.71-0.81), p<.001	0.35 (0.16-0.78), p=1
Q7[a], no. (%)	5929 (85.03%)	5630 (87.27%)	24 (75%)
Adjusted OR (95% CI), *P* value	1 [Reference]	0.88 (0.82-0.94), p<.009	1.06 (0.5-2.24), p=1
Q8[a], no. (%)	6050 (86.76%)	5733 (88.87%)	24 (75%)
Adjusted OR (95% CI), *P* value	1 [Reference]	0.76 (0.7-0.81), p<.001	0.44 (0.2-1), p=1
Q9[a], no. (%)	5815 (83.39%)	5601 (86.82%)	24 (75%)
Adjusted OR (95% CI), *P* value	1 [Reference]	0.85 (0.84-0.86), p<.001	0.38 (0.17-0.84), p=1
Q10[a], no. (%)	5804 (83.24%)	5591 (86.67%)	24 (75%)
Adjusted OR (95% CI), *P* value	1 [Reference]	0.77 (0.72-0.83), p<.001	0.47 (0.21-1.03), p=1
Q11[a], no. (%)	5806 (83.26%)	5596 (86.75%)	24 (75%)
Adjusted OR (95% CI), *P* value	1 [Reference]	0.85 (0.84-0.86), p<.001	0.59 (0.58-0.6), p<.001
Q12[b], no. (%)	5657 (81.13%)	5460 (84.64%)	24 (75%)
Adjusted OR (95% CI), *P* value	1 [Reference]	1.02 (0.93-1.12), p=1	1.25 (0.47-1.34), p=1
Q13[c], no. (%)	5533 (79.35%)	5362 (83.12%)	24 (75%)
Adjusted OR 1 (95% CI), *P* value	1 [Reference]	1.02 (0.93-1.12), p=1	1.25 (0.47-3.34), p=1
Adjusted OR 2 (95% CI), *P* value	1 [Reference]	0.97 (0.89-1.06), p=1	0.78 (0.3-2), p=1
Q14[a], no. (%)	5712 (81.92%)	5493 (85.15%)	24 (75%)
Adjusted OR (95% CI), *P* value	1 [Reference]	0.97 (0.89-1.06), p=1	0.74 (0.28-1.94), p=1
Q15[a], no. (%)	5673 (81.36%)	5463 (84.68%)	23 (71.88%)
Adjusted OR (95% CI), *P* value	1 [Reference]	0.79 (0.78-0.79), p<.001	0.54 (0.24-1.22), p=11
Q16[a], no. (%)	5631 (80.75%)	5404 (83.77%)	24 (75%)
Adjusted OR (95% CI), *P* value	1 [Reference]	0.81 (0.75-0.88), p<.001	0.82 (0.35-1.91), p=1
Q17[a], no. (%)	5688 (81.57%)	5464 (84.7%)	24 (75%)
Adjusted OR (95% CI), *P* value	1 [Reference]	0.9 (0.83-0.96), p=0.74	0.59 (0.28-1.27), p=1
Q18[a], no. (%)	5674 (81.37%)	5481 (84.96%)	24 (75%)

© 2025 Busch F et al. *JAMA Network Open*.

eTable 6. Adjusted odds ratios for self-reported gender from regression analyses

Adjusted OR (95% CI), *P* value	1 [Reference]	0.67 (0.62-0.72), p<.001	0.48 (0.23-1), p=.05
Q19[a], no. (%)	5741 (82.33%)	5516 (85.51%)	24 (75%)
Adjusted OR (95% CI), *P* value	1 [Reference]	0.73 (0.67-0.78), p<.001	0.56 (0.27-1.18), p=1
Q20[a], no. (%)	5734 (82.23%)	5506 (85.35%)	24 (75%)
Adjusted OR (95% CI), *P* value	1 [Reference]	0.7 (0.65-0.75), p<.001	0.56 (0.27-1.18), p=1

[a] Cumulative link mixed models were used for ordinal regression analysis.
[b] A binary mixed-effects regression analysis with the reference category "A high degree of accuracy, the decision path is clearly comprehensible (explainable AI)." compared to "A higher accuracy, the decision path however, is not comprehensible." was performed.
[c] Two binary mixed-effects regression analyses with the reference category "The AI gives a false alarm about as often as it misses a diagnosis." compared to "The AI misses almost no diagnosis, but often gives a false alarm." (adjusted OR 1) and "The AI almost never gives a false alarm, but sometimes misses a diagnosis." (adjusted OR 2) were performed.
Abbreviations: CI, confidence interval; OR, odds ratio; Q1, What are your general views on the use of artificial intelligence (AI) in medicine?; Q2, Artificial intelligence (AI) should be increasingly used in the healthcare sector.; Q3, How much confidence do you have that AI can improve healthcare?; Q4, How much do you trust an AI to provide reliable information about your health?; Q5, How much do you trust an AI to provide accurate information about your diagnosis?; Q6, How much do you trust an AI to provide accurate information about your response to therapy?; Q7, Which of the following statements about a potential application of AI in medicine do you most likely agree with?; Q8, I would trust a highly accurate AI to make a vital decision for me.; Q9, How would you rate it if a certified AI software analyzes X-ray images?; Q10, How would you rate it if a certified AI software diagnoses cancer?; Q11, How would you rate it if a certified AI software would be available to doctors as a second opinion?; Q12/Q13, Suppose an AI makes a diagnosis. What would you prefer?; Q14, Suppose an AI has about the same accuracy as doctors. Which situation for a diagnosis would you prefer?; Q15, What do you think of healthcare facilities (clinics, practices) using AI software to aid in diagnosis?; Q16, Would you prefer to visit healthcare facilities that use AI software? Q17, How concerned are you about AI compromising the protection of your personal data?; Q18, How concerned are you that the use of AI will reduce the contact between physicians and patients?; Q19, How concerned are you that AI could replace human doctors in the future?; Q20, How concerned are you that the use of AI will lead to higher healthcare costs?.

© 2025 Busch F et al. *JAMA Network Open*.

eTable 7. Adjusted odds ratios for self-reported health status from regression analyses

	Very poor (N=203)	Poor (N=1221)	Sufficient (N=4244)	Good (N=5439)	Very good (N=2553)
Q1[a], no. (%)	183 (90.15%)	1061 (86.9%)	3699 (87.16%)	4728 (86.93%)	2227 (87.23%)
Adjusted OR (95% CI), *P* value	0.15 (0.11-0.21), p<.001	0.54 (0.46-0.63), p<.001	0.56 (0.5-0.62), p<.001	0.71 (0.64-0.78), p<.001	1 [Reference]
Q2[a], no. (%)	182 (89.66%)	1043 (85.42%)	3653 (86.07%)	4638 (85.27%)	2201 (86.21%)
Adjusted OR (95% CI), *P* value	0.21 (0.15-0.3), p<.001	0.62 (0.53-0.72), p<.001	0.62 (0.55-0.69), p<.001	0.77 (0.67-0.85), p<.001	1 [Reference]
Q3[a], no. (%)	181 (89.16%)	1052 (86.16%)	3685 (86.83%)	4726 (86.89%)	2226 (87.19%)
Adjusted OR (95% CI), *P* value	0.21 (0.16-0.3), p<.001	0.57 (0.49-0.67), p<.001	0.62 (0.56-0.7), p<.001	0.75 (0.67-0.82), p<.001	1 [Reference]
Q4[a], no. (%)	181 (89.16%)	1048 (85.83%)	3675 (86.59%)	4722 (86.82%)	2225 (87.15%)
Adjusted OR (95% CI), *P* value	0.27 (0.19-0.37), p<.001	0.61 (0.52-0.71), p<.001	0.61 (0.54-0.68), p<.001	0.78 (0.71-0.86), p<.001	1 [Reference]
Q5[a], no. (%)	180 (88.67%)	1047 (85.75%)	3676 (86.62%)	4723 (86.84%)	2225 (87.15%)
Adjusted OR (95% CI), *P* value	0.31 (0.23-0.42), p<.001	0.6 (0.52-0.7), p<.001	0.7 (0.6-0.75), p<.001	0.85 (0.77-0.93), p=.24	1 [Reference]
Q6 [a], no. (%)	181 (89.16%)	1044 (85.5%)	3671 (86.5%)	4720 (86.78%)	2223 (87.07%)
Adjusted OR (95% CI), *P* value	0.28 (0.2-0.38), p<.001	0.59 (0.51-0.69), p<.001	0.62 (0.55-0.69), p<.001	0.7 (0.7-0.85), p<.001	1 [Reference]
Q7[a], no. (%)	181 (89.16%)	1022 (83.7%)	3596 (84.73%)	4607 (84.7%)	2177 (85.27%)
Adjusted OR (95% CI), *P* value	1.1 (0.8-1.5), p=1	0.93 (0.8-1.09), p=1	0.93 (0.83-1.03), p=1	0.98 (0.89-1.08), p=1	1 [Reference]
Q8[a], no. (%)	182 (89.66%)	1044 (85.5%)	3670 (86.48%)	4692 (86.27%)	2219 (86.92%)
Adjusted OR (95% CI), *P* value	0.45 (0.33-0.62), p<.001	0.74 (0.63-0.86), p=0.02	0.71 (0.64-0.79), p<.001	0.88 (0.8-0.97), p=1	1 [Reference]
Q9[a], no. (%)	176 (86.7%)	998 (81.74%)	3542 (83.46%)	4539 (83.45%)	2185 (85.59%)
Adjusted OR (95% CI), *P* value	0.2 (0.2-0.21), p<.001	0.61 (0.6-0.62), p<.001	0.59 (0.59-0.6), p<.001	0.77 (0.71-0.82), p<.001	1 [Reference]
Q10[a], no. (%)	177 (87.19%)	995 (81.49%)	3527 (83.11%)	4536 (83.4%)	2184 (85.55%)
Adjusted OR (95% CI), *P* value	0.35 (0.25-0.48), p<.001	0.7 (0.6-0.82), p=.002	0.67 (0.6-0.74), p<.001	0.83 (0.75-0.91), p=.04	1 [Reference]
Q11[a], no. (%)	176 (86.7%)	995 (81.49%)	3535 (83.29%)	4535 (83.38%)	2185 (85.59%)
Adjusted OR (95% CI), *P* value	0.33 (0.33-0.34), p<.001	0.78 (0.77-0.79), p<.001	0.74 (0.73-0.75), p<.001	0.89 (0.83-0.95), p=.29	1 [Reference]
Q12[b], no. (%)	172 (84.73%)	964 (78.95%)	3452 (81.34%)	4412 (81.12%)	2141 (83.86%)
Adjusted OR (95% CI), *P* value	1.27 (0.83-1.94), p=1	1.09 (0.89-1.33), p=1	1.34 (0.99-1.31), p=1	1.07 (0.94-1.21), p=1	1 [Reference]
Q13[c], no. (%)	168 (82.76%)	939 (76.9%)	3394 (79.97%)	4323 (79.48%)	2095 (82.06%)

© 2025 Busch F et al. *JAMA Network Open*.

eTable 7. Adjusted odds ratios for self-reported health status from regression analyses

	Very poor (N=203)	Poor (N=1221)	Sufficient (N=4244)	Good (N=5439)	Very good (N=2553)
Adjusted OR 1 (95% CI), *P* value	1.27 (0.83-1.94), p=1	1.09 (0.89-1.33), p=1	1.14 (0.99-1.31), p=1	1.07 (0.94-1.21), p=1	1 [Reference]
Adjusted OR 2 (95% CI), *P* value	1.1 (0.74-1.65), p=1	1.07 (0.89-1.29), p=1	1.08 (0.95-1.23), p=1	1.1 (0.98-1.23), p=1	1 [Reference]
Q14[a], no. (%)	172 (84.73%)	975 (79.85%)	3476 (81.9%)	4462 (82.04%)	2144 (83.98%)
Adjusted OR (95% CI), *P* value	1.14 (0.77-1.67), p=1	1.03 (0.85-1.67), p=1	0.86 (0.75-0.98), p=1	0.95 (0.84-1.07), p=1	1 [Reference]
Q15[a], no. (%)	170 (83.74%)	979 (80.18%)	3463 (81.6%)	4428 (81.41%)	2119 (83%)
Adjusted OR (95% CI), *P* value	0.31 (0.31-0.31), p<.001	0.68 (0.68-0.69), p<.001	0.66 (0.65-0.67), p<.001	0.84 (0.83-0.85), p<.001	1 [Reference]
Q16[a], no. (%)	170 (83.74%)	963 (78.87%)	3424 (80.68%)	4394 (80.79%)	2108 (82.57%)
Adjusted OR (95% CI), *P* value	0.84 (0.51-1.2), p=1	0.85 (0.72-1), p=1	0.78 (0.69-0.88), p=.01	0.9 (0.81-1), p=1	1 [Reference]
Q17[a], no. (%)	172 (84.73%)	978 (80.1%)	3467 (81.69%)	4433 (81.5%)	2126 (83.27%)
Adjusted OR (95% CI), *P* value	1.48 (1.07-2.04), p=1	1.29 (1.1-1.5), p=.35	1.77 (1.05-1.31), p=.99	1.07 (1.97-1.19), p=1	1 [Reference]
Q18[a], no. (%)	172 (84.73%)	980 (80.26%)	3466 (81.67%)	4435 (81.54%)	2126 (83.27%)
Adjusted OR (95% CI), *P* value	1.76 (1.26-2.46), p=.23	1.22 (1.04-1.42), p=1	1.09 (0.97-1.21), p=1	1.07 (0.97-1.18), p=1	1 [Reference]
Q19[a], no. (%)	172 (84.73%)	982 (80.43%)	3485 (82.12%)	4480 (82.37%)	2162 (84.68%)
Adjusted OR (95% CI), *P* value	1.94 (0.96-1.87), p=1	1.1 (0.94-1.28), p=1	1.1 (0.99-1.23), p=1	1.11 (1.01-1.23), p=1	1 [Reference]
Q20[a], no. (%)	172 (84.73%)	979 (80.18%)	3477 (81.93%)	4477 (82.31%)	2159 (84.57%)
Adjusted OR (95% CI), *P* value	1 (1.43-2.8), p=.01	1.15 (0.98-1.34), p=1	1.02 (0.92-1.14), p=1	1.02 (0.92-1.12), p=1	1 [Reference]

[a] Cumulative link mixed models were used for ordinal regression analysis.
[b] A binary mixed-effects regression analysis with the reference category "A high degree of accuracy, the decision path is clearly comprehensible (explainable AI)." compared to "A higher accuracy, the decision path however, is not comprehensible." was performed.
[c] Two binary mixed-effects regression analyses with the reference category "The AI gives a false alarm about as often as it misses a diagnosis." compared to "The AI misses almost no diagnosis, but often gives a false alarm." (adjusted OR 1) and "The AI almost never gives a false alarm, but sometimes misses a diagnosis." (adjusted OR 2) were performed.
Abbreviations: CI, confidence interval; OR, odds ratio; Q1, What are your general views on the use of artificial intelligence (AI) in medicine?; Q2, Artificial intelligence (AI) should be increasingly used in the healthcare sector.; Q3, How much confidence do you have that AI can improve healthcare?; Q4, How much do you trust an AI to provide reliable information about your health?; Q5, How much do you trust an AI to provide accurate information about your diagnosis?; Q6, How much do you trust an AI to provide accurate information about your response to therapy?; Q7, Which of the following statements about a potential application of AI in medicine do you most likely agree with?; Q8, I would trust a highly accurate AI to make a vital decision for me.; Q9, How would you rate it if a certified AI software analyzes X-ray images?; Q10, How would you rate it if a certified AI software diagnoses cancer?; Q11, How would you rate it if a certified AI software would be available to doctors as a second opinion?; Q12/Q13, Suppose an AI makes a diagnosis. What would you prefer?; Q14, Suppose an AI has about the same accuracy as doctors. Which situation for a diagnosis would you prefer?; Q15, What do you think of healthcare facilities (clinics, practices) using AI software to aid in diagnosis?; Q16, Would you prefer to visit healthcare facilities that use AI software? Q17, How concerned are you about AI compromising the protection of your personal data?; Q18, How concerned are you that the use of AI will reduce the contact between physicians and patients?; Q19, How concerned are you that AI could replace human doctors in the future?; Q20, How concerned are you that the use of AI will lead to higher healthcare costs.

© 2025 Busch F et al. *JAMA Network Open*.

eTable 8. Adjusted odds ratios for self-reported AI knowledge from regression analyses

	No knowledge (N=1848)	Little knowledge (N=8097)	Good knowledge (N=3423)	Expert (N=211)
Q1[a], no. (%)	1532 (82.9%)	7175 (88.61%)	3003 (87.73%)	188 (89.1%)
Adjusted OR (95% CI), *P* value	1 [Reference]	1.75 (1.56-1.96), p<.001	3.17 (2.77-3.63), p<.001	7.11 (5.19-9.74), p<.001
Q2[a], no. (%)	1495 (80.9%)	7055 (87.13%)	2978 (87%)	189 (89.57%)
Adjusted OR (95% CI), *P* value	1 [Reference]	1.38 (1.24-1.54), p<.001	1.48 (1.32-1.67), p<.001	3.8 (2.77-5.2), p<.001
Q3[a], no. (%)	1531 (82.85%)	7155 (88.37%)	2995 (87.5%)	189 (89.57%)
Adjusted OR (95% CI), *P* value	1 [Reference]	1.46 (1.3-1.64), p<.001	2.53 (2.21-2.89), p<.001	5.11 (3.76-6.94), p<.001
Q4[a], no. (%)	1525 (82.52%)	7143 (88.22%)	2994 (87.47%)	189 (89.57%)
Adjusted OR (95% CI), *P* value	1 [Reference]	1.51 (1.34-1.69), p<.001	2.41 (2.11-2.76), p<.001	3.26 (2.41-4.41), p<.001
Q5[a], no. (%)	1528 (82.68%)	7141 (88.19%)	2994 (87.47%)	188 (89.1%)
Adjusted OR (95% CI), *P* value	1 [Reference]	1.43 (1.27-1.6), p<.001	2.27 (1.98-2.59), p<.001	3.96 (2.91-5.4), p<.001
Q6 [a], no. (%)	1527 (82.63%)	7130 (88.06%)	2993 (87.44%)	189 (89.57%)
Adjusted OR (95% CI), *P* value	1 [Reference]	1.43 (1.27-1.6), p<.001	2.27 (1.99-2.6), p<.001	3.44 (2.53-4.67), p<.001
Q7[a], no. (%)	1493 (80.79%)	6976 (86.16%)	2930 (85.6%)	184 (87.2%)
Adjusted OR (95% CI), *P* value	1 [Reference]	1.89 (1.68-2.12), p<.001	2.8 (2.45-3.2), p<.001	4.48 (3.26-6.15), p<.001
Q8[a], no. (%)	1523 (82.41%)	7108 (87.79%)	2987 (87.26%)	189 (89.57%)
Adjusted OR (95% CI), *P* value	1 [Reference]	1.18 (1.06-1.32), p=.83	1.74 (1.53-1.98), p<.001	2.06 (1.52-2.8), p<.001
Q9[a], no. (%)	1484 (80.3%)	6869 (84.83%)	2904 (84.84%)	183 (86.73%)
Adjusted OR (95% CI), *P* value	1 [Reference]	1.23 (1.22-1.24), p<.001	1.98 (1.95-2), p<.001	4.44 (3.31-5.97), p<.001
Q10[a], no. (%)	1477 (79.92%)	6864 (84.77%)	2895 (84.57%)	183 (86.73%)
Adjusted OR (95% CI), *P* value	1 [Reference]	1.32 (1.17-1.48), p<.001	1.91 (1.67-2.19), p<.001	3.36 (2.46-4.59), p<.001
Q11[a], no. (%)	1473 (79.71%)	6870 (84.85%)	2901 (84.75%)	182 (86.26%)
Adjusted OR (95% CI), *P* value	1 [Reference]	1.35 (1.34-1.37), p<.001	1.96 (1.93-1.98), p<.001	4.29 (4.23-4.35), p<.001
Q12[b], no. (%)	1423 (77%)	6690 (82.62%)	2848 (83.2%)	180 (85.31%)
Adjusted OR (95% CI), *P* value	1 [Reference]	0.86 (0.75-1), p=1	0.72 (0.61-0.69), p=.04	1.06 (0.72-0.85), p=1
Q13[c], no. (%)	1400 (75.76%)	6544 (80.82%)	2799 (81.77%)	176 (83.41%)
Adjusted OR 1 (95% CI), *P* value	1 [Reference]	0.86 (0.75-1), p=1	0.72 (0.61-0.69), p=.04	1.06 (0.72-0.85), p=1
Adjusted OR 2 (95% CI), *P* value	1 [Reference]	1.08 (0.94-1.24), p=1	1.11 (0.95-1.3), p=1	0.97 (0.67-1.4), p=1
Q14[a], no. (%)	1440 (77.92%)	6740 (83.24%)	2866 (83.73%)	183 (86.73%)
Adjusted OR (95% CI), *P* value	1 [Reference]	1.23 (1.06-1.43), p=1	1.3 (1.1-1.53), p=.7	1.65 (1.16-2.36), p=1
Q15[a], no. (%)	1410 (76.3%)	6723 (83.03%)	2846 (83.14%)	180 (85.31%)
Adjusted OR (95% CI), *P* value	1 [Reference]	1.45 (1.44-1.46), p<.001	2.3 (2.28-2.32), p<.001	4.77 (3.51-6.48), p<.001
Q16[a], no. (%)	1401 (75.81%)	6651 (82.14%)	2827 (82.59%)	180 (85.31%)
Adjusted OR (95% CI), *P* value	1 [Reference]	1.38 (1.21-1.56), p<.001	1.99 (1.72-2.3), p<.001	3.18 (2.29-4.42), p<.001
Q17[a], no. (%)	1414 (76.52%)	6728 (83.09%)	2852 (83.32%)	182 (86.26%)
Adjusted OR (95% CI), *P* value	1 [Reference]	0.94 (0.84-1.06), p=1	1.15 (1-1.31), p=1	1.03 (0.75-1.39), p=1
Q18[a], no. (%)	1417 (76.68%)	6730 (83.12%)	2850 (83.26%)	182 (86.26%)

© 2025 Busch F et al. *JAMA Network Open*.

eTable 8. Adjusted odds ratios for self-reported AI knowledge from regression analyses

	No knowledge (N=1848)	Little knowledge (N=8097)	Good knowledge (N=3423)	Expert (N=211)
Adjusted OR (95% CI), *P* value	1 [Reference]	1.02 (0.91-1.15), p=1	1.45 (1.27-1.66), p<.001	1.46 (1.08-2), p=1
Q19[a], no. (%)	1460 (79%)	6770 (83.61%)	2868 (83.79%)	183 (86.73%)
Adjusted OR (95% CI), *P* value	1 [Reference]	0.94 (0.84-1.01), p=1	1.3 (1.14-1.49), p=.03	1.84 (1.35-2.5), p=.03
Q20[a], no. (%)	1455 (78.73%)	6763 (83.52%)	2865 (83.7%)	181 (85.78%)
Adjusted OR (95% CI), *P* value	1 [Reference]	1.09 (0.97-1.22), p=1	1.6 (1.4-1.8), p<.001	1.83 (1.34-2.5), p=.001

[a] Cumulative link mixed models were used for ordinal regression analysis.
[b] A binary mixed-effects regression analysis with the reference category "A high degree of accuracy, the decision path is clearly comprehensible (explainable AI)." compared to "A higher accuracy, the decision path however, is not comprehensible." was performed.
[c] Two binary mixed-effects regression analyses with the reference category "The AI gives a false alarm about as often as it misses a diagnosis." compared to "The AI misses almost no diagnosis, but often gives a false alarm." (adjusted OR 1) and "The AI almost never gives a false alarm, but sometimes misses a diagnosis." (adjusted OR 2) were performed.
Abbreviations: CI, confidence interval; OR, odds ratio; Q1, What are your general views on the use of artificial intelligence (AI) in medicine?; Q2, Artificial intelligence (AI) should be increasingly used in the healthcare sector.; Q3, How much confidence do you have that AI can improve healthcare?; Q4, How much do you trust an AI to provide reliable information about your health?; Q5, How much do you trust an AI to provide accurate information about your diagnosis?; Q6, How much do you trust an AI to provide accurate information about your response to therapy?; Q7, Which of the following statements about a potential application of AI in medicine do you most likely agree with?; Q8, I would trust a highly accurate AI to make a vital decision for me.; Q9, How would you rate it if a certified AI software analyzes X-ray images?; Q10, How would you rate it if a certified AI software diagnoses cancer?; Q11, How would you rate it if a certified AI software would be available to doctors as a second opinion?; Q12/Q13, Suppose an AI makes a diagnosis. What would you prefer?; Q14, Suppose an AI has about the same accuracy as doctors. Which situation for a diagnosis would you prefer?; Q15, What do you think of healthcare facilities (clinics, practices) using AI software to aid in diagnosis?; Q16, Would you prefer to visit healthcare facilities that use AI software? Q17, How concerned are you about AI compromising the protection of your personal data?; Q18, How concerned are you that the use of AI will reduce the contact between physicians and patients?; Q19, How concerned are you that AI could replace human doctors in the future?; Q20, How concerned are you that the use of AI will lead to higher healthcare costs?.

© 2025 Busch F et al. *JAMA Network Open*.

eTable 9. Adjusted odds ratios for age and number of technical devices used weekly from regression analyses

	Age (N=12452)	Total number tech devices (N=13752)
Q1[a], no. (%)	11898 (95.55%)	11898 (86.52%)
Adjusted OR (95% CI), *P* value	0.99 (0.78-1.26), p=1	1.17 (1.13-1.21), p<.001
Q2[a], no. (%)	11717 (94.10%)	11717 (85.20%)
Adjusted OR (95% CI), *P* value	1.07 (0.84-1.36), p=1	1.14 (1.1-1.18), p<.001
Q3[a], no. (%)	11870 (95.33%)	11870 (86.31%)
Adjusted OR (95% CI), *P* value	0.77 (0.61-0.98), p=1	1.15 (1.11-1.19), p<.001
Q4[a], no. (%)	11851 (95.17%)	11851 (86.18%)
Adjusted OR (95% CI), *P* value	0.95 (0.75-1.2), p=1	1.13 (1.09-1.17), p<.001
Q5[a], no. (%)	11851 (95.17%)	11851 (86.18%)
Adjusted OR (95% CI), *P* value	1.43 (1.27-1.6), p=1	1.1 (1.06-1.14), p<.001
Q6 [a], no. (%)	11839 (95.08%)	11839 (86.09%)
Adjusted OR (95% CI), *P* value	0.88 (0.7-1.12), p=1	1.07 (1.04-1.11), p=.0.2
Q7[a], no. (%)	11583 (93.02%)	11583 (84.23%)
Adjusted OR (95% CI), *P* value	1.62 (1.28-2.06), p<.02	1.13 (1.09-1.17), p<.001
Q8[a], no. (%)	11807 (94.82%)	11807 (85.86%)
Adjusted OR (95% CI), *P* value	1.41 (1.11-1.78), p=1	1.18 (1.06-1.02), p=1
Q9[a], no. (%)	11440 (91.87%)	11440 (83.19%)
Adjusted OR (95% CI), *P* value	0.89 (0.88-0.9), p<.001	1.09 (1.08-1.11), p<.001
Q10[a], no. (%)	11419 (91.70%)	11419 (83.04%)
Adjusted OR (95% CI), *P* value	1.32 (1.17-1.48), p=.13	1.32 (1.17-1.13), p<.001
Q11[a], no. (%)	11426 (91.76%)	11426 (83.09%)
Adjusted OR (95% CI), *P* value	1.05 (1.03-1.06), p<.001	1.15 (1.14-1.17), p<.001
Q12[b], no. (%)	11141 (89.47%)	11141 (81.01%)
Adjusted OR (95% CI), *P* value	1.62 (1.18-2.15), p=.76	0.91 (0.87-0.95), p=.02
Q13[c], no. (%)	10919 (87.69%)	10919 (79.40%)
Adjusted OR 1 (95% CI), *P* value	1.12 (1.18-2.21), p=.76	0.91 (0.87-0.95), p=.02
Adjusted OR 2 (95% CI), *P* value	2.12 (1.59-2.83), p<.001	0.92 (0.88-0.96), p=.01
Q14[a], no. (%)	11229 (90.18%)	11229 (81.65%)
Adjusted OR (95% CI), *P* value	0.5 (0.37-0.66), p<.001	0.98 (0.94-1.03), p=1
Q15[a], no. (%)	11159 (89.62%)	11159 (81.14%)
Adjusted OR (95% CI), *P* value	0.91 (0.9-0.92), p<.001	1.16 (1.15-1.17), p<.001
Q16[a], no. (%)	11059 (88.81%)	11059 (80.42%)
Adjusted OR (95% CI), *P* value	0.83 (0.64-1.07), p=1	1.05 (1.02-1.09), p=1
Q17[a], no. (%)	11176 (89.75%)	11176 (81.27%)
Adjusted OR (95% CI), *P* value	0.62 (0.49-0.79), p=.03	1.09 (1.06-1.13), p<.001
Q18[a], no. (%)	11179 (89.78%)	11179 (81.29%)
Adjusted OR (95% CI), *P* value	0.36 (0.29-0.46), p<.001	1.03 (1-1.07), p=1
Q19[a], no. (%)	11281 (90.60%)	11281 (82.03%)
Adjusted OR (95% CI), *P* value	0.39 (0.3-0.49), p<.001	1.06 (1.03-1.1), p=.12
Q20[a], no. (%)	11264 (90.46%)	11264 (81.91%)
Adjusted OR (95% CI), *P* value	0.5 (0.4-0.64), p<.001	1.06 (1.03-1.1), p=.21

[a] Cumulative link mixed models were used for ordinal regression analysis.
[b] A binary mixed-effects regression analysis with the reference category "A high degree of accuracy, the decision path is clearly comprehensible (explainable AI)." compared to "A higher accuracy, the decision path however, is not comprehensible." was performed.
[c] Two binary mixed-effects regression analyses with the reference category "The AI gives a false alarm about as often as it misses a diagnosis." compared to "The AI misses almost no diagnosis, but often gives a false alarm." (adjusted OR 1) and "The AI almost never gives a false alarm, but sometimes misses a diagnosis." (adjusted OR 2) were performed.
Abbreviations: CI, confidence interval; OR, odds ratio; Q1, What are your general views on the use of artificial intelligence (AI) in medicine?; Q2, Artificial intelligence (AI) should be increasingly used in the healthcare sector.; Q3, How much confidence do you have that AI can improve healthcare?; Q4, How much do you trust an AI to provide reliable information about your health?; Q5, How much do you trust an AI to provide accurate information about your diagnosis?; Q6, How much do you trust an AI to provide accurate information about your response to therapy?; Q7, Which of the following statements about a potential application of AI in medicine do you most likely agree with?; Q8, I would trust a highly accurate AI to make a vital decision for me.; Q9, How would you rate it if a certified AI software analyzes X-ray images?; Q10, How would you rate it if a certified AI software diagnoses cancer?; Q11, How would you rate it if a certified AI software would be available to doctors as a second opinion?; Q12/Q13, Suppose an AI makes a diagnosis. What would you prefer?; Q14, Suppose an AI has about the same accuracy as doctors. Which situation for a diagnosis would you prefer?; Q15, What do you think of healthcare facilities (clinics,

practices) using AI software to aid in diagnosis?; Q16, Would you prefer to visit healthcare facilities that use AI software? Q17, How concerned are you about AI compromising the protection of your personal data?; Q18, How concerned are you that the use of AI will reduce the contact between physicians and patients?; Q19, How concerned are you that AI could replace human doctors in the future?; Q20, How concerned are you that the use of AI will lead to higher healthcare costs?.

© 2025 Busch F et al. *JAMA Network Open*.

eTable 10. Adjusted odds ratios for highest educational level from regression analyses

	Elementary School (N=1192)	Middle School (N=2844)	High School (N=4142)	University (N=5403)
Q1[a], no. (%)	961 (80.62%)	2427 (85.34%)	3783 (91.33%)	4727 (87.49%)
Adjusted OR (95% CI), *P* value	1 [Reference]	0.93 (0.81-1.08), p=1	1.03 (0.89-1.19), p=1	1.27 (1.1-1.48), p=1
Q2[a], no. (%)	922 (77.35%)	2356 (82.84%)	3748 (90.49%)	4691 (86.82%)
Adjusted OR (95% CI), *P* value	1 [Reference]	0.88 (0.76-1.01), p=1	1.02 (0.89-1.17), p=1	1.05 (0.91-1.2), p=1
Q3[a], no. (%)	955 (80.12%)	2423 (85.2%)	3777 (91.19%)	4715 (87.27%)
Adjusted OR (95% CI), *P* value	1 [Reference]	0.9 (0.78-1.04), p=1	0.94 (0.81-1.1), p=1	1.15 (1-1.33), p=1
Q4[a], no. (%)	951 (79.78%)	2411 (84.77%)	3774 (91.12%)	4715 (87.27%)
Adjusted OR (95% CI), *P* value	1 [Reference]	0.86 (0.76-1.01), p=1	0.98 (0.93-1.06), p=1	1.03 (0.89-1.19), p=1
Q5[a], no. (%)	952 (79.87%)	2411 (84.77%)	3774 (91.12%)	4714 (87.25%)
Adjusted OR (95% CI), *P* value	1 [Reference]	0.86 (0.74-0.99), p=1	0.86 (0.74-1), p=1	0.97 (0.84-1.12), p=1
Q6 [a], no. (%)	944 (79.19%)	2414 (84.88%)	3770 (91.02%)	4711 (87.19%)
Adjusted OR (95% CI), *P* value	1 [Reference]	0.84 (0.73-0.97), p=1	0.81 (0.7-0.94), p=1	0.93 (0.81-1.08), p=1
Q7[a], no. (%)	915 (76.76%)	2361 (83.02%)	3695 (89.21%)	4612 (85.36%)
Adjusted OR (95% CI), *P* value	1 [Reference]	1.09 (0.94-1.26), p=1	1.38 (1.19-1.6), p=.005	1.68 (1.45-1.95), p<.001
Q8[a], no. (%)	942 (79.03%)	2404 (84.53%)	3766 (90.92%)	4695 (86.9%)
Adjusted OR (95% CI), *P* value	1 [Reference]	0.84 (0.73-0.97), p=1	0.78 (0.68-0.9), p=.17	0.81 (0.85-0.93), p=.93
Q9[a], no. (%)	898 (75.34%)	2306 (81.08%)	3700 (89.33%)	4536 (83.95%)
Adjusted OR (95% CI), *P* value	1 [Reference]	0.88 (0.88-0.9), p<.001	0.98 (0.97-0.99), p=1	1.22 (1.21-1.24), p<.001
Q10[a], no. (%)	896 (75.17%)	2297 (80.77%)	3693 (89.16%)	4533 (83.9%)
Adjusted OR (95% CI), *P* value	1 [Reference]	0.86 (0.74-1), p=1	0.91 (0.79-1.06), p=1	0.99 (0.85-1.14), p=1
Q11[a], no. (%)	895 (75.08%)	2301 (80.91%)	3690 (89.09%)	4540 (84.03%)
Adjusted OR (95% CI), *P* value	1 [Reference]	0.98 (0.97-1), p=1	1.08 (1.07-1.1), p<.001	1.37 (1.35-1.39), p<.001
Q12[b], no. (%)	859 (72.06%)	2206 (77.57%)	3602 (86.96%)	4474 (82.81%)
Adjusted OR (95% CI), *P* value	1 [Reference]	1.03 (0.86-1.24), p=1	1.09 (0.9-1.31), p=1	1 (0.83-1.2), p=1
Q13[c], no. (%)	822 (68.96%)	2161 (75.98%)	3549 (85.68%)	4387 (81.2%)
Adjusted OR 1 (95% CI), *P* value	1 [Reference]	1.03 (0.86-1.24), p=1	1.09 (0.9-1.31), p=1	1 (0.82-1.2), p=1
Adjusted OR 2 (95% CI), *P* value	1 [Reference]	0.92 (0.77-1.1), p=1	0.97 (0.81-1.56), p=1	1 (0.84-1.2), p=1
Q14[a], no. (%)	872 (73.15%)	2253 (79.22%)	3636 (87.78%)	4468 (82.69%)
Adjusted OR (95% CI), *P* value	1 [Reference]	0.86 (0.72-1.03), p=1	0.82 (0.68-0.99), p=1	0.76 (0.64-0.92), p=1
Q15[a], no. (%)	874 (73.32%)	2254 (79.25%)	3600 (86.91%)	4431 (82.01%)
Adjusted OR (95% CI), *P* value	1 [Reference]	0.81 (0.8-0.81), p<.001	0.93 (0.92-0.94), p<.001	1.18 (1.17-1.19), p<.001
Q16[a], no. (%)	854 (71.64%)	2234 (78.55%)	3564 (86.05%)	4407 (81.57%)
Adjusted OR (95% CI), *P* value	1 [Reference]	0.84 (0.72-1), p=1	0.91 (0.77-1.07), p=1	1.16 (0.98-1.37), p=1
Q17[a], no. (%)	873 (73.24%)	2248 (79.04%)	3611 (87.18%)	4444 (82.25%)
Adjusted OR (95% CI), *P* value	1 [Reference]	1.08 (0.93-1.25), p=1	1.03 (0.89-1.2), p=1	1.03 (0.89-1.19), p=1
Q18[a], no. (%)	871 (73.07%)	2255 (79.29%)	3607 (87.08%)	4446 (82.29%)

© 2025 Busch F et al. *JAMA Network Open*.

eTable 10. Adjusted odds ratios for highest educational level from regression analyses

	Elementary School (N=1192)	Middle School (N=2844)	High School (N=4142)	University (N=5403)
Adjusted OR (95% CI), *P* value	1 [Reference]	0.91 (0.78-1.06), p=1	0.86 (0.74-1), p=1	0.87 (0.74-1.01), p=1
Q19[a], no. (%)	880 (73.83%)	2270 (79.82%)	3655 (88.24%)	4476 (82.84%)
Adjusted OR (95% CI), *P* value	1 [Reference]	1.01 (0.87-1.17), p=1	0.94 (0.8-1.09), p=1	1.13 (0.97-1.3), p=1
Q20[a], no. (%)	879 (73.74%)	2271 (79.85%)	3657 (88.29%)	4457 (82.49%)
Adjusted OR (95% CI), *P* value	1 [Reference]	1.14 (0.99-1.33), p=1	1.06 (0.91-1.23), p=1	1.27 (1.11-1.5), p=.27

[a] Cumulative link mixed models were used for ordinal regression analysis.

[b] A binary mixed-effects regression analysis with the reference category "A high degree of accuracy, the decision path is clearly comprehensible (explainable AI)." compared to "A higher accuracy, the decision path however, is not comprehensible." was performed.

[c] Two binary mixed-effects regression analyses with the reference category "The AI gives a false alarm about as often as it misses a diagnosis." compared to "The AI misses almost no diagnosis, but often gives a false alarm." (adjusted OR 1) and "The AI almost never gives a false alarm, but sometimes misses a diagnosis." (adjusted OR 2) were performed.

Abbreviations: CI, confidence interval; OR, odds ratio; Q1, What are your general views on the use of artificial intelligence (AI) in medicine?; Q2, Artificial intelligence (AI) should be increasingly used in the healthcare sector.; Q3, How much confidence do you have that AI can improve healthcare?; Q4, How much do you trust an AI to provide reliable information about your health?; Q5, How much do you trust an AI to provide accurate information about your diagnosis?; Q6, How much do you trust an AI to provide accurate information about your response to therapy?; Q7, Which of the following statements about a potential application of AI in medicine do you most likely agree with?; Q8, I would trust a highly accurate AI to make a vital decision for me.; Q9, How would you rate it if a certified AI software analyzes X-ray images?; Q10, How would you rate it if a certified AI software diagnoses cancer?; Q11, How would you rate it if a certified AI software would be available to doctors as a second opinion?; Q12/Q13, Suppose an AI makes a diagnosis. What would you prefer?; Q14, Suppose an AI has about the same accuracy as doctors. Which situation for a diagnosis would you prefer?; Q15, What do you think of healthcare facilities (clinics, practices) using AI software to aid in diagnosis?; Q16, Would you prefer to visit healthcare facilities that use AI software? Q17, How concerned are you about AI compromising the protection of your personal data?; Q18, How concerned are you that the use of AI will reduce the contact between physicians and patients?; Q19, How concerned are you that AI could replace human doctors in the future?; Q20, How concerned are you that the use of AI will lead to higher healthcare costs?

© 2025 Busch F et al. *JAMA Network Open*.

eTable 11. Absolute survey results for each item stratified by gender

	Male (N=6973)	Female (N=6451)	Diverse (N=32)
Q1, no. (%)	6864 (98.44%)	6318 (97.94%)	31 (96.88%)
Extremely negative	150 (2.19%)	141 (2.23%)	2 (6.45%)
Rather negative	417 (6.08%)	596 (9.43%)	5 (16.13%)
Neutral	2240 (32.63%)	2070 (32.76%)	6 (19.35%)
Rather positive	2836 (41.32%)	2593 (41.04%)	11 (35.48%)
Extremely positive	1221 (17.79%)	918 (14.53%)	7 (22.58%)
Q2, no. (%)	6708 (96.20%)	6302 (97.69%)	29 (90.63%)
Completely disagree	167 (2.49%)	252 (4%)	3 (10.34%)
Tend to disagree	454 (6.77%)	590 (9.36%)	7 (24.14%)
Neutral	1727 (25.75%)	1657 (26.29%)	4 (13.79%)
Tend to agree	2549 (38 %)	2503 (39.72%)	6 (20.69%)
Agree completely	1811 (27 %)	1300 (20.63%)	9 (31.03%)
Q3, no. (%)	6861 (98.39%)	6357 (98.54%)	30 (93.75%)
Very little	219 (3.19 %)	296 (4.66%)	3 (10%)
Little	668 (9.74 %)	850 (13.37%)	4 (13.33%)
Medium	2448 (35.68%)	2349 (36.95%)	10 (33.33%)
Much	2361 (34.41%)	2122 (33.38%)	4 (13.33%)
Very much	1165 (16.98%)	740 (11.64%)	9 (30%)
Q4, no. (%)	6847 (98.19%)	6344 (98.34%)	30 (93.75%)
Very little	219 (3.2%)	331 (5.22%)	3 (10%)
Little	888 (12.97%)	938 (14.79%)	6 (20%)
Medium	2551 (37.26%)	2492 (39.28%)	11 (36.67%)
Much	2427 (35.45%)	2079 (32.77%)	5 (16.67%)
Very much	762 (11.13%)	504 (7.94%)	5 (16.67%)
Q5, no. (%)	6838 (98.06%)	6342 (98.31%)	30 (93.75%)
Very little	243 (3.55%)	343 (5.41%)	3 (10%)
Little	875 (12.8%)	1007 (15.88%)	6 (20%)
Medium	2522 (36.88%)	2473 (38.99%)	12 (40%)
Much	2412 (35.27%)	2015 (31.77%)	5 (16.67%)
Very much	786 (11.49%)	504 (7.95%)	4 (13.33%)
Q6, no. (%)	6826 (97.89%)	6337 (98.23%)	30 (93.75%)
Very little	244 (3.57%)	355 (5.6%)	6 (20%)
Little	915 (13.4%)	1013 (15.99%)	4 (13.33%)
Medium	2591 (37.96%)	2566 (40.49%)	11 (36.67%)
Much	2339 (34.27%)	1927 (30.41%)	4 (13.33%)
Very much	737 (10.8%)	476 (7.51%)	5 (16.67%)
Q7, no. (%)	6656 (95.45%)	6185 (95.88%)	28 (87.50%)
No use of AI independent of the disease.	690 (10.37%)	756 (12.22%)	3 (10.71%)
Use only for minor illnesses (eg, cold).	1698 (25.51%)	1876 (30.33%)	5 (17.86%)
Use also for moderately severe diseases (eg, appendicitis).	1872 (28.13%)	1549 (25.04%)	10 (35.71%)
Use also for severe diseases (eg, cancer, traffic accidents).	2396 (36%)	2004 (32.4%)	10 (35.71%)
Q8, no. (%)	6812 (97.69%)	6310 (97.81%)	29 (90.63%)
Completely disagree	629 (9.23%)	765 (12.12%)	7 (24.14%)
Tend to disagree	1197 (17.57%)	1380 (21.87%)	5 (17.24%)
Neutral	1942 (28.51%)	1650 (26.15%)	3 (10.34%)
Tend to agree	2257 (33.13%)	1992 (31.57%)	8 (17.24%)
Agree completely	787 (11.55%)	523 (8.29%)	6 (24.14%)

eTable 11. Absolute survey results for each item stratified by gender

	Male (N=6973)	Female (N=6451)	Diverse (N=32)
Q9, no. (%)	6486 (93.02%)	6145 (95.26%)	30 (93.75%)
Extremely negative	152 (2.34%)	220 (3.58%)	3 (10%)
Rather negative	516 (7.96%)	684 (11.13%)	5 (16.67%)
Neutral	1886 (29.08 %)	1698 (27.63%)	8 (26.67%)
Rather positive	2651 (40.87%)	2542 (41.37%)	8 (26.67%)
Extremely positive	1281 (19.75%)	1001 (16.29%)	6 (20%)
Q10, no. (%)	6466 (92.73%)	6134 (95.09%)	30 (93.75%)
Extremely negative	206 (3.19%)	346 (5.64%)	3 (10%)
Rather negative	724 (11.2%)	820 (13.37%)	6 (20%)
Neutral	1853 (28.66%)	1781 (29.03%)	6 (20%)
Rather positive	2491 (38.52%)	2311 (37.68%)	10 (33.33%)
Extremely positive	1192 (18.43%)	876 (14.28%)	5 (16.67%)
Q11, no. (%)	6472 (92.82%)	6142 (95.21%)	30 (93.75%)
Extremely negative	141 (2.18%)	190 (3.09%)	3 (10%)
Rather negative	346 (5.35 %)	473 (7.7%)	3 (10%)
Neutral	1489 (23.01%)	1403 (22.84%)	5 (16.67%)
Rather positive	2672 (41.29%)	2576 (41.94%)	10 (33.33%)
Extremely positive	1824 (28.18%)	1500 (24.42%)	9 (30%)
Q12, no. (%)	6284 (90.12%)	5964 (92.45%)	30 (93.75%)
A high degree of accuracy, the decision path is clearly comprehensible (explainable AI).	4333 (68.95%)	4271 (71.61%)	21 (70%)
A higher accuracy, the decision path however, is not comprehensible.	1951 (31.05%)	1693 (28.39%)	9 (30%)
Q13, no. (%)	6117 (87.72%)	5832 (90.4%)	30 (93.75%)
The AI misses almost no diagnosis, but often gives a false alarm.	2792 (45.64%)	2761 (47.34%)	17 (56.67%)
The AI almost never gives a false alarm, but sometimes misses a diagnosis.	2260 (36.95%)	2077 (35.61%)	9 (30%)
The AI gives a false alarm about as often as it misses a diagnosis.	1065 (17.41%)	994 (17.04%)	4 (13.33%)
Q14, no. (%)	6341 (90.94%)	5976 (92.64%)	30 (93.75%)
Physicians make the diagnosis alone.	379 (5.98%)	435 (7.28%)	4 (13.33%)
AI and physicians make the diagnosis together. Doctors make the final decision.	4715 (74.36%)	4301 (71.97%)	18 (60%)
AI and physicians make the diagnosis together. Both have equal authority.	787 (12.41%)	875 (14.64%)	4 (13.33%)
AI and physicians make the diagnosis together. The AI makes the final decision.	160 (2.52%)	143 (2.39%)	5 (16.67%)
AI makes the diagnosis alone.	290 (4.57%)	222 (3.71%)	2 (6.67%)

© 2025 Busch F et al. *JAMA Network Open*.

eTable 11. Absolute survey results for each item stratified by gender

	Male (N=6973)	Female (N=6451)	Diverse (N=32)
Q15, no. (%)	6329 (90.76%)	5990 (92.85%)	29 (90.63%)
Extremely negative	140 (2.21%)	177 (2.95%)	1 (3.45%)
Rather negative	420 (6.64%)	455 (7.6%)	4 (13.79%)
Neutral	1728 (27.3%)	1773 (29.6%)	9 (31.03%)
Rather positive	2609 (41.22%)	2502 (41.77%)	7 (24.14%)
Extremely positive	1432 (22.63%)	1083 (18.08%)	8 (27.59%)
Q16, no. (%)	6263 (89.82%)	5912 (91.64%)	30 (93.75%)
Always prefer facilities without AI.	324 (5.17%)	431 (7.29%)	3 (10%)
Tend to prefer facilities without AI.	1321 (21.09%)	1420 (24.02%)	5 (16.67%)
Rather prefer facilities with AI.	3584 (57.22%)	3296 (55.75%)	14 (46.67%)
Always prefer facilities with AI.	1034 (16.51%)	765 (12.94%)	8 (26.67%)
Q17, no. (%)	6339 (90.91%)	5993 (92.9%)	30 (93.75%)
Very worried	1051 (16.58%)	1208 (20.16%)	8 (26.67%)
Somewhat concerned	2131 (33.62%)	2161 (36.06%)	9 (30%)
Neutral	1908 (30.1%)	1601 (26.71%)	4 (13.33%)
Rather unconcerned	856 (13.5%)	731 (12.2%)	5 (16.67%)
Unconcerned	393 (6.2%)	292 (4.87%)	4 (13.33%)
Q18, no. (%)	6320 (90.64%)	6012 (93.19%)	30 (93.75%)
Very worried	1305 (20.65%)	1737 (28.89%)	8 (26.67%)
Somewhat concerned	2337 (36.98%)	2251 (37.44%)	12 (40%)
Neutral	1659 (26.25%)	1217 (20.24%)	6 (20%)
Rather unconcerned	746 (11.8%)	613 (10.2%)	2 (6.67%)
Unconcerned	273 (4.32%)	194 (3.23%)	2 (6.67%)
Q19, no. (%)	6392 (91.67%)	6043 (93.68%)	30 (93.75%)
Very worried	1587 (24.83%)	2185 (36.16%)	9 (30%)
Somewhat concerned	2085 (32.62%)	1844 (30.51%)	12 (40%)
Neutral	1440 (22.53%)	1077 (17.82%)	6 (20%)
Rather unconcerned	875 (13.69%)	649 (10.74%)	1 (3.33%)
Unconcerned	505 (7.9%)	288 (4.77%)	2 (6.67%)
Q20, no. (%)	6381 (91.51%)	6035 (93.55%)	30 (93.75%)
Very worried	1412 (22.13%)	1841 (30.51%)	6 (20%)
Somewhat concerned	1976 (30.97%)	1899 (31.47%)	11 (36.67%)
Neutral	1804 (28.27%)	1470 (24.36%)	8 (26.67%)
Rather unconcerned	779 (12.21%)	547 (9.06%)	4 (13.33%)
Unconcerned	410 (6.43%)	278 (4.61%)	1 (3.33%)

Abbreviations: Q1, What are your general views on the use of artificial intelligence (AI) in medicine?; Q2, Artificial intelligence (AI) should be increasingly used in the healthcare sector.; Q3, How much confidence do you have that AI can improve healthcare?; Q4, How much do you trust an AI to provide reliable information about your health?; Q5, How much do you trust an AI to provide accurate information about your diagnosis?; Q6, How much do you trust an AI to provide accurate information about your response to therapy?; Q7, Which of the following statements about a potential application of AI in medicine do you most likely agree with?; Q8, I would trust a highly accurate AI to make a vital decision for me.; Q9, How would you rate it if a certified AI software analyzes X-ray images?; Q10, How would you rate it if a certified AI software diagnoses cancer?; Q11, How would you rate it if a certified AI software would be available to doctors as a second opinion?; Q12/Q13, Suppose an AI makes a diagnosis. What would you prefer?; Q14, Suppose an AI has about the same accuracy as doctors. Which situation for a diagnosis would you prefer?; Q15, What do you think of healthcare facilities (clinics, practices) using AI software to aid in diagnosis?; Q16, Would you prefer to visit healthcare facilities that use AI software? Q17, How concerned are you about AI compromising the protection of your personal data?; Q18, How concerned are you that the use of AI will reduce the contact between physicians and patients?; Q19, How concerned are you that AI could replace human doctors in the future?; Q20, How concerned are you that the use of AI will lead to higher healthcare costs?.

© 2025 Busch F et al. *JAMA Network Open*.

eTable 12. Absolute survey results for each item stratified by self-reported health status

	Very poor (N=203)	Poor (N=1221)	Sufficient (N=4244)	Good (N=5439)	Very good (N=2553)
Q1, no. (%)	199 (98.03%)	1185 (97.05%)	4160 (98.02%)	5351 (98.38%)	2538 (99.41%)
Extremely negative	53 (26.63%)	78 (6.58%)	77 (1.85%)	54 (1.01%)	33 (1.3%)
Rather negative	58 (29.15%)	231 (19.49%)	282 (6.78%)	318 (5.94%)	134 (5.28%)
Neutral	52 (26.13%)	390 (32.91%)	1700 (40.87%)	1628 (30.42%)	608 (23.96%)
Rather positive	22 (11.06%)	324 (27.34%)	1592 (38.27%)	2547 (47.6%)	1035 (40.78%)
Extremely positive	14 (7.04%)	162 (13.67%)	509 (12.27%)	804 (15.03%)	718 (28.29%)
Q2, no. (%)	198 (97.54%)	1169 (95.74%)	4114 (96.94%)	5258 (96.67%)	2501 (97.96%)
Completely disagree	64 (32.32%)	106 (9.07%)	113 (2.75%)	89 (1.69%)	54 (2.16%)
Tend to disagree	49 (24.75%)	231 (19.76%)	303 (7.37%)	327 (6.22%)	140 (5.6%)
Neutral	39 (19.7%)	295 (25.24%)	1334 (32.43%)	1284 (24.42%)	475 (18.99%)
Tend to agree	22 (11.11%)	297 (25.41%)	1560 (37.92%)	2296 (43.67%)	961 (38.42%)
Agree completely	24 (12.12%)	240 (20.53%)	804 (19.54%)	1262 (24%)	871 (34.83%)
Q3, no. (%)	195 (96.06%)	1190 (97.46%)	4164 (98.11%)	5377 (98.86%)	2534 (99.26%)
Very little	57 (29.23%)	86 (7.23%)	157 (3.77%)	147 (2.73%)	77 (3.04%)
Little	49 (25.13%)	290 (24.37%)	462 (11.1%)	521 (9.69%)	212 (8.37%)
Medium	53 (27.18%)	385 (32.35%)	1829 (43.92%)	1884 (35.04%)	713 (28.14%)
Much	24 (12.31%)	288 (24.2%)	1238 (29.73%)	2113 (39.3%)	884 (34.89%)
Very much	12 (6.15%)	141 (11.85%)	428 (10.28%)	712 (13.24%)	648 (25.57%)
Q4, no. (%)	196 (96.55%)	1184 (96.97%)	4144 (97.64%)	5375 (98.82%)	2530 (99.1%)
Very little	48 (24.49%)	82 (6.39%)	177 (4.27%)	156 (2.9%)	99 (3.91%)
Little	57 (29.08%)	272 (22.97%)	619 (14.94%)	647 (12.04%)	255 (10.08%)
Medium	57 (29.08%)	433 (36.57%)	1775 (42.83%)	2046 (38.07%)	797 (31.5%)
Much	22 (11.22%)	290 (24.49%)	1275 (30.77%)	2032 (37.8%)	949 (37.51%)
Very much	12 (6.12%)	107 (9.04%)	298 (7.19%)	494 (9.19%)	430 (17%)
Q5, no. (%)	194 (95.57%)	1183 (96.89%)	4143 (97.62%)	5373 (98.79%)	2531 (99.14%)
Very little	35 (18.04%)	71 (6%)	185 (4.47%)	183 (3.41%)	120 (4.74%)
Little	64 (32.99%)	285 (24.09%)	648 (15.64%)	644 (11.99%)	268 (10.59%)
Medium	62 (31.96%)	437 (36.94%)	1709 (41.25%)	2039 (37.95%)	807 (31.88%)
Much	23 (11.86%)	290 (24.51%)	1282 (30.94%)	2005 (37.32%)	900 (35.56%)
Very much	10 (5.15%)	98 (8.28%)	321 (7.75%)	502 (9.34%)	436 (17.23%)
Q6, no. (%)	195 (96.06%)	1176 (96.31%)	4139 (97.53%)	5368 (98.69%)	2529 (99.06%)
Very little	38 (19.49%)	77 (6.55%)	190 (4.59%)	191 (3.56%)	115 (4.55%)
Little	62 (31.79%)	269 (22.87%)	648 (15.66%)	691 (12.87%)	276 (10.91%)
Medium	61 (31.28%)	442 (37.59%)	1791 (43.27%)	2130 (39.68%)	809 (31.99%)
Much	24 (12.31%)	295 (25.09%)	1204 (29.09%)	1897 (35.34%)	909 (35.94%)
Very much	10 (5.13%)	93 (7.91%)	306 (7.39%)	459 (8.55%)	420 (16.61%)
Q7, no. (%)	195 (96.06%)	1146 (93.86%)	4033 (95.03%)	5222 (96.01%)	2473 (96.87%)
No use of AI independent of the disease.	15 (7.69%)	138 (12.04%)	474 (11.75%)	527 (10.09%)	339 (13.71%)
Use only for minor illnesses (eg, cold).	71 (36.41%)	316 (27.57%)	1191 (29.53%)	1472 (28.19%)	595 (24.06%)

© 2025 Busch F et al. *JAMA Network Open*.

eTable 12. Absolute survey results for each item stratified by self-reported health status

	Very poor (N=203)	Poor (N=1221)	Sufficient (N=4244)	Good (N=5439)	Very good (N=2553)
Use also for moderately severe diseases (eg, appendicitis).	51 (26.15%)	315 (27.49%)	1081 (26.8%)	1444 (27.65%)	598 (24.18%)
Use also for severe diseases (eg, cancer, traffic accidents).	58 (29.74%)	377 (32.9%)	1287 (31.91%)	1779 (34.07%)	941 (38.05%)
Q8, no. (%)	196 (96.55%)	1172 (95.99%)	4129 (97.29%)	5338 (98.14%)	2526 (98.94%)
Completely disagree	52 (26.53%)	154 (13.14%)	438 (10.61%)	489 (9.16%)	261 (10.33%)
Tend to disagree	65 (33.16%)	315 (26.88%)	781 (18.91%)	1018 (19.07%)	414 (16.39%)
Neutral	29 (14.8%)	305 (26.02%)	1331 (32.24%)	1399 (26.21%)	478 (18.92%)
Tend to agree	35 (17.86%)	271 (23.12%)	1258 (30.47%)	1920 (35.97%)	868 (34.36%)
Agree completely	16 (8.16%)	127 (10.84%)	321 (7.77%)	512 (9.59%)	405 (16.03%)
Q9, no. (%)	190 (93.6%)	1119 (91.65%)	3950 (93.07%)	5127 (94.26%)	2478 (97.06%)
Extremely negative	42 (22.11%)	96 (8.58%)	102 (2.58%)	82 (1.6%)	51 (2.06%)
Rather negative	52 (27.37%)	196 (17.52%)	413 (10.46%)	397 (7.74%)	161 (6.5%)
Neutral	50 (26.32%)	297 (26.54%)	1387 (35.11%)	1368 (26.68%)	541 (21.83%)
Rather positive	28 (14.74%)	358 (31.99%)	1492 (37.77%)	2341 (45.66%)	1039 (41.93%)
Extremely positive	18 (9.47%)	172 (15.37%)	556 (14.08%)	939 (18.31%)	686 (27.68%)
Q10, no. (%)	191 (94.09%)	1116 (91.4%)	3930 (92.6%)	5121 (94.15%)	2475 (96.94%)
Extremely negative	41 (21.47%)	110 (9.86%)	163 (4.15%)	153 (2.99%)	91 (3.68%)
Rather negative	41 (21.47%)	197 (17.65%)	558 (14.2%)	536 (10.47%)	235 (9.49%)
Neutral	61 (31.94%)	309 (27.69%)	1279 (32.54%)	1460 (28.51%)	595 (24.04%)
Rather positive	34 (17.8%)	340 (30.47%)	1423 (36.21%)	2109 (41.18%)	959 (38.75%)
Extremely positive	14 (7.33%)	160 (14.34%)	507 (12.9%)	863 (16.85%)	595 (24.04%)
Q11, no. (%)	190 (93.6%)	1116 (91.4%)	3941 (92.86%)	5117 (94.08%)	2477 (97.02%)
Extremely negative	41 (21.58%)	76 (6.81%)	83 (2.11%)	78 (1.52%)	61 (2.46%)
Rather negative	52 (27.37%)	178 (15.95%)	230 (5.84%)	255 (4.98%)	115 (4.64%)
Neutral	40 (21.05%)	143 (12.81%)	1133 (28.75%)	1064 (20.79%)	469 (18.93%)
Rather positive	35 (18.42%)	373 (33.42%)	1658 (42.07%)	2307 (45.09%)	958 (38.68%)
Extremely positive	22 (11.58%)	246 (22.04%)	837 (21.24%)	1413 (27.61%)	876 (35.37%)
Q12, no. (%)	184 (90.64%)	1075 (88.04%)	3828 (90.2%)	4953 (91.06%)	2418 (94.71%)
A high degree of accuracy, the decision path is clearly	140 (76.09%)	786 (73.12%)	2548 (66.56%)	3532 (71.31%)	1745 (72.17%)

© 2025 Busch F et al. *JAMA Network Open*.

eTable 12. Absolute survey results for each item stratified by self-reported health status

	Very poor (N=203)	Poor (N=1221)	Sufficient (N=4244)	Good (N=5439)	Very good (N=2553)
comprehensible (explainable AI).					
A higher accuracy, the decision path however, is not comprehensible.	44 (23.91%)	189 (17.58%)	1280 (33.44%)	1421 (28.69%)	653 (27.01%)
Q13, no. (%)	177 (87.19%)	1037 (84.93%)	3760 (88.6%)	4842 (89.02%)	2363 (92.56%)
The AI misses almost no diagnosis, but often gives a false alarm.	96 (54.24%)	470 (45.32%)	1633 (43.43%)	2272 (46.92%)	1192 (50.44%)
The AI almost never gives a false alarm, but sometimes misses a diagnosis.	51 (28.81%)	343 (33.08%)	1425 (37.9%)	1779 (36.74%)	827 (35%)
The AI gives a false alarm about as often as it misses a diagnosis.	30 (16.95%)	224 (21.6%)	702 (18.67%)	791 (16.34%)	344 (14.56%)
Q14, no. (%)	184 (90.64%)	1076 (88.12%)	3842 (90.53%)	5018 (92.26%)	2423 (94.91%)
Physicians make the diagnosis alone.	8 (4.35%)	74 (6.88%)	293 (7.63%)	302 (6.02%)	144 (5.94%)
AI and physicians make the diagnosis together. Doctors make the final decision.	9 (4.89%)	30 (2.79%)	2790 (72.62%)	3701 (73.75%)	1727 (71.28%)
AI and physicians make the diagnosis together. Both have equal authority.	27 (14.67%)	158 (14.68%)	540 (14.06%)	687 (13.69%)	300 (12.38%)
AI and physicians make the	138 (75%)	783 (72.77%)	84 (2.19%)	125 (2.49%)	68 (2.81%)

© 2025 Busch F et al. *JAMA Network Open*.

eTable 12. Absolute survey results for each item stratified by self-reported health status

	Very poor (N=203)	Poor (N=1221)	Sufficient (N=4244)	Good (N=5439)	Very good (N=2553)
diagnosis together. The AI makes the final decision.					
AI makes the diagnosis alone.	2 (1.09%)	31 (2.88%)	135 (3.51%)	203 (4.05%)	184 (7.59%)
Q15, no. (%)	182 (89.66%)	1096 (89.76%)	3860 (90.95%)	5005 (92.02%)	2406 (94.24%)
Extremely negative	57 (31.32%)	84 (7.66%)	79 (2.05%)	50 (1%)	47 (1.95%)
Rather negative	42 (23.08%)	179 (16.33%)	325 (8.42%)	251 (5.01%)	90 (3.74%)
Neutral	35 (19.23%)	309 (28.19%)	1331 (34.48%)	1367 (27.31%)	525 (21.82%)
Rather positive	30 (16.48%)	346 (31.57%)	1504 (38.96%)	2281 (45.57%)	1024 (42.56%)
Extremely positive	18 (9.89%)	178 (16.24%)	621 (16.09%)	1056 (21.1%)	720 (29.93%)
Q16, no. (%)	182 (89.66%)	1075 (88.04%)	3805 (89.66%)	4953 (91.06%)	2386 (93.46%)
Always prefer facilities without AI.	18 (9.89%)	108 (10.05%)	247 (6.49%)	258 (5.21%)	134 (5.62%)
Tend to prefer facilities without AI.	25 (13.74%)	271 (25.21%)	933 (24.52%)	1089 (21.99%)	466 (19.53%)
Rather prefer facilities with AI.	99 (54.4%)	545 (50.7%)	2188 (57.5%)	2862 (57.78%)	1280 (53.65%)
Always prefer facilities with AI.	40 (21.98%)	151 (14.05%)	437 (11.48%)	744 (15.02%)	506 (21.21%)
Q17, no. (%)	184 (90.64%)	1094 (89.6%)	3863 (91.02%)	5007 (92.06%)	2412 (94.48%)
Very worried	25 (13.59%)	184 (16.82%)	611 (15.82%)	928 (18.53%)	552 (22.89%)
Somewhat concerned	61 (33.15%)	327 (29.89%)	1344 (34.79%)	1806 (36.07%)	842 (34.91%)
Neutral	38 (20.65%)	335 (30.62%)	1211 (31.35%)	1417 (28.3%)	567 (23.51%)
Rather unconcerned	38 (20.65%)	159 (14.53%)	474 (12.27%)	341 (6.81%)	297 (12.31%)
Unconcerned	22 (11.96%)	89 (8.14%)	223 (5.77%)	215 (4.29%)	154 (6.38%)
Q18, no. (%)	183 (90.15%)	1096 (89.76%)	3861 (90.98%)	5012 (92.15%)	2409 (94.36%)
Very worried	25 (13.66%)	271 (24.73%)	878 (22.74%)	1215 (24.24%)	702 (29.14%)
Somewhat concerned	55 (30.05%)	358 (32.66%)	1468 (38.02%)	1984 (39.59%)	797 (33.08%)
Neutral	37 (20.22%)	254 (23.18%)	1007 (26.08%)	1127 (22.49%)	512 (21.25%)
Rather unconcerned	41 (22.4%)	153 (13.96%)	394 (10.2%)	534 (10.65%)	265 (11%)
Unconcerned	25 (13.66%)	60 (5.47%)	114 (2.95%)	152 (3.03%)	133 (5.52%)
Q19, no. (%)	184 (90.64%)	1097 (89.84%)	3881 (91.45%)	5058 (93%)	2443 (95.69%)
Very worried	43 (23.37%)	333 (30.36%)	1107 (28.52%)	1536 (30.37%)	806 (32.99%)
Somewhat concerned	40 (21.74%)	330 (30.08%)	1256 (32.36%)	1664 (32.9%)	697 (28.53%)
Neutral	31 (16.85%)	217 (19.78%)	886 (22.83%)	898 (17.75%)	453 (18.54%)

eTable 12. Absolute survey results for each item stratified by self-reported health status

	Very poor (N=203)	Poor (N=1221)	Sufficient (N=4244)	Good (N=5439)	Very good (N=2553)
Rather unconcerned	40 (21.74%)	146 (13.31%)	439 (11.31%)	608 (12.02%)	326 (13.34%)
Unconcerned	30 (16.3%)	71 (6.47%)	139 (3.58%)	261 (1.16%)	161 (6.59%)
Q20, no. (%)	184 (90.64%)	1095 (89.68%)	3870 (91.19%)	5053 (92.9%)	2444 (95.73%)
Very worried	38 (20.65%)	275 (25.11%)	993 (25.66%)	1282 (25.37%)	717 (29.34%)
Somewhat concerned	34 (18.48%)	321 (29.32%)	1261 (32.58%)	1656 (32.77%)	692 (28.31%)
Neutral	32 (17.39%)	189 (17.26%)	1071 (27.67%)	1343 (26.58%)	588 (24.06%)
Rather unconcerned	38 (20.65%)	128 (11.69%)	366 (9.46%)	532 (10.53%)	284 (11.62%)
Unconcerned	42 (22.83%)	82 (7.49%)	178 (4.6%)	240 (4.75%)	163 (6.67%)

Abbreviations: Q1, What are your general views on the use of artificial intelligence (AI) in medicine?; Q2, Artificial intelligence (AI) should be increasingly used in the healthcare sector.; Q3, How much confidence do you have that AI can improve healthcare?; Q4, How much do you trust an AI to provide reliable information about your health?; Q5, How much do you trust an AI to provide accurate information about your diagnosis?; Q6, How much do you trust an AI to provide accurate information about your response to therapy?; Q7, Which of the following statements about a potential application of AI in medicine do you most likely agree with?; Q8, I would trust a highly accurate AI to make a vital decision for me.; Q9, How would you rate it if a certified AI software analyzes X-ray images?; Q10, How would you rate it if a certified AI software diagnoses cancer?; Q11, How would you rate it if a certified AI software would be available to doctors as a second opinion?; Q12/Q13, Suppose an AI makes a diagnosis. What would you prefer?; Q14, Suppose an AI has about the same accuracy as doctors. Which situation for a diagnosis would you prefer?; Q15, What do you think of healthcare facilities (clinics, practices) using AI software to aid in diagnosis?; Q16, Would you prefer to visit healthcare facilities that use AI software? Q17, How concerned are you about AI compromising the protection of your personal data?; Q18, How concerned are you that the use of AI will reduce the contact between physicians and patients?; Q19, How concerned are you that AI could replace human doctors in the future?; Q20, How concerned are you that the use of AI will lead to higher healthcare costs?.

© 2025 Busch F et al. *JAMA Network Open*.

eTable 13. Absolute survey results for each item stratified by self-reported AI knowledge

	No knowledge (N=1848)	Little knowledge (N=8097)	Good knowledge (N=3423)	Expert (N=211)
Q1, no. (%)	1755 (94.97%)	8054 (99.47%)	3402 (99.39%)	210 (99.53%)
Extremely negative	78 (4.44%)	122 (1.51%)	84 (2.47%)	7 (3.33%)
Rather negative	151 (8.6%)	669 (8.31%)	196 (5.76%)	9 (4.29%)
Neutral	859 (48.95%)	2879 (35.75%)	620 (18.22%)	19 (9.05%)
Rather positive	398 (22.68%)	3396 (42.17%)	1647 (48.41%)	80 (38.1%)
Extremely positive	269 (15.33%)	988 (12.27%)	855 (25.13%)	95 (45.24%)
Q2, no. (%)	1703 (92.15%)	7898 (97.54%)	3367 (98.36%)	210 (99.53%)
Completely disagree	90 (5.28%)	205 (2.6%)	115 (3.42%)	8 (3.81%)
Tend to disagree	137 (8.04%)	692 (8.76%)	212 (6.3%)	9 (4.29%)
Neutral	714 (41.93%)	2183 (27.64%)	493 (14.64%)	26 (12.38%)
Tend to agree	388 (22.78%)	3259 (41.26%)	1409 (41.85%)	63 (30%)
Agree completely	374 (21.96%)	1559 (19.74%)	1138 (33.8%)	104 (49.52%)
Q3, no. (%)	1756 (95.02%)	8021 (99.06%)	3396 (99.21%)	211 (100%)
Very little	135 (7.69%)	269 (3.35%)	106 (3.12%)	7 (3.32%)
Little	283 (16.12%)	959 (11.96%)	256 (7.54%)	14 (6.64%)
Medium	708 (40.32%)	3247 (40.48%)	873 (25.71%)	27 (12.8%)
Much	386 (21.98%)	2651 (33.05%)	1404 (41.34%)	73 (34.6%)
Very much	244 (13.9%)	895 (11.16%)	757 (22.29%)	90 (42.65%)
Q4, no. (%)	1748 (94.59%)	8008 (98.9%)	3390 (99.04%)	210 (99.53%)
Very little	143 (8.18%)	284 (3.55%)	116 (3.42%)	6 (2.86%)
Little	411 (23.51%)	1075 (13.42%)	324 (9.56%)	21 (10%)
Medium	613 (35.07%)	3407 (42.54%)	1033 (30.47%)	52 (24.76%)
Much	354 (20.25%)	2674 (33.39%)	1432 (42.24%)	77 (36.67%)
Very much	227 (12.99%)	568 (7.09%)	485 (14.31%)	54 (25.71%)
Q5, no. (%)	1750 (94.7%)	7999 (98.79%)	3393 (99.12%)	208 (98.58%)
Very little	138 (7.89%)	315 (3.94%)	130 (3.83%)	4 (1.92%)
Little	418 (23.89%)	1119 (13.99%)	324 (9.55%)	25 (12.02%)
Medium	610 (34.86%)	3366 (42.08%)	1044 (30.77%)	42 (20.19%)
Much	364 (20.8%)	2598 (32.48%)	1423 (41.94%)	75 (36.06%)
Very much	220 (12.57%)	601 (7.51%)	472 (13.91%)	62 (29.81%)
Q6, no. (%)	1750 (94.7%)	7985 (98.62%)	3390 (99.04%)	210 (99.53%)
Very little	149 (8.51%)	312 (3.91%)	140 (4.13%)	6 (2.86%)
Little	428 (24.46%)	1142 (14.3%)	328 (9.68%)	26 (12.38%)
Medium	593 (33.89%)	3482 (43.61%)	1098 (32.39%)	50 (23.81%)
Much	255 (14.57%)	2482 (31.08%)	1392 (41.06%)	74 (35.24%)
Very much	225 (12.86%)	567 (7.1%)	432 (12.74%)	54 (25.71%)
Q7, no. (%)	1701 (92.05%)	6686 (82.57%)	3319 (96.96%)	204 (96.68%)
No use of AI independent of the disease.	455 (26.75%)	736 (11.01%)	276 (8.32%)	11 (5.39%)
Use only for minor illnesses (eg, cold).	595 (34.98%)	2316 (34.64%)	678 (20.43%)	45 (22.06%)
Use also for moderately severe diseases (eg, appendicitis).	277 (16.28%)	2251 (33.67%)	911 (27.45%)	33 (16.18%)
Use also for severe diseases (eg, cancer, traffic accidents).	374 (21.99%)	2482 (37.12%)	1454 (43.81%)	115 (56.37%)
Q8, no. (%)	1745 (94.43%)	7954 (98.23%)	3383 (98.83%)	210 (99.53%)
Completely disagree	202 (11.58%)	805 (10.12%)	348 (10.29%)	25 (11.9%)
Tend to disagree	271 (15.53%)	1674 (21.05%)	605 (17.88%)	38 (18.1%)
Neutral	644 (36.91%)	2247 (28.25%)	706 (20.87%)	27 (12.86%)

© 2025 Busch F et al. *JAMA Network Open*.

eTable 13. Absolute survey results for each item stratified by self-reported AI knowledge

	No knowledge (N=1848)	Little knowledge (N=8097)	Good knowledge (N=3423)	Expert (N=211)
Tend to agree	367 (21.03%)	2622 (32.96%)	1269 (37.51%)	146 (69.52%)
Agree completely	261 (14.96%)	606 (%)	455 (13.45%)	46 (21.9%)
Q9, no. (%)	1698 (91.88%)	7642 (94.38%)	3248 (94.89%)	205 (97.16%)
Extremely negative	90 (5.3%)	188 (2.46%)	88 (2.71%)	6 (29.93%)
Rather negative	161 (9.48%)	799 (10.46%)	224 (6.9%)	9 (4.39%)
Neutral	657 (38.69%)	2336 (30.57%)	614 (18.9%)	25 (12.2%)
Rather positive	459 (27.03%)	3225 (32.2%)	1490 (45.87%)	122 (59.51%)
Extremely positive	331 (19.49%)	1094 (14.32%)	832 (25.62%)	103 (50.24%)
Q10, no. (%)	1691 (91.5%)	7630 (94.23%)	3239 (94.62%)	204 (96.68%)
Extremely negative	113 (6.68%)	305 (4%)	117 (3.61%)	10 (4.9%)
Rather negative	296 (17.5%)	959 (12.57%)	295 (9.11%)	15 (7.35%)
Neutral	534 (31.58%)	2398 (31.43%)	721 (22.26%)	32 (15.69%)
Rather positive	430 (25.43%)	2911 (38.15%)	1433 (44.24%)	65 (31.86%)
Extremely positive	318 (18.81%)	1057 (13.85%)	673 (20.78%)	82 (40.2%)
Q11, no. (%)	1684 (91.13%)	7639 (94.34%)	3246 (94.83%)	203 (96.21%)
Extremely negative	76 (4.51%)	173 (2.26%)	81 (2.5%)	8 (3.94%)
Rather negative	132 (4.84%)	513 (6.72%)	170 (5.24%)	18 (8.87%)
Neutral	613 (36.4%)	1842 (24.11%)	461 (14.2%)	19 (9.36%)
Rather positive	500 (29.69%)	3357 (43.95%)	1393 (42.91%)	50 (24.63%)
Extremely positive	363 (21.56%)	1754 (22.96%)	1141 (35.15%)	121 (59.61%)
Q12, no. (%)	1611 (87.18%)	7397 (91.35%)	3182 (92.96%)	201 (95.26%)
A high degree of accuracy, the decision path is clearly comprehensible (explainable AI).	985 (61.14%)	5155 (69.69%)	2394 (75.24%)	150 (74.63%)
A higher accuracy, the decision path however, is not comprehensible.	626 (38.86%)	2242 (30.31%)	788 (24.76%)	51 (25.37%)
Q13, no. (%)	1584 (85.71%)	7212 (89.07%)	3118 (91.09%)	194 (91.94%)
The AI misses almost no diagnosis, but often gives a false alarm.	628 (39.65%)	3362 (46.62%)	1506 (48.3%)	118 (60.82%)
The AI almost never gives a false alarm, but sometimes misses a diagnosis.	631 (39.84%)	2592 (35.94%)	1131 (36.27%)	58 (29.9%)
The AI gives a false alarm about as often as it misses a diagnosis.	325 (20.52%)	1258 (17.44%)	481 (15.43%)	18 (9.28%)
Q14, no. (%)	1643 (88.91%)	7484 (92.43%)	3204 (93.6%)	204 (96.68%)
Physicians make the diagnosis alone.	246 (14.97%)	424 (5.67%)	150 (4.68%)	5 (2.45%)
AI and physicians make the diagnosis together. Doctors make the final decision.	968 (58.92%)	5590 (74.69%)	2422 (75.59%)	153 (75%)
AI and physicians make the diagnosis	205 (12.48%)	1049 (14.02%)	437 (13.64%)	19 (9.31%)

© 2025 Busch F et al. *JAMA Network Open*.

eTable 13. Absolute survey results for each item stratified by self-reported AI knowledge

	No knowledge (N=1848)	Little knowledge (N=8097)	Good knowledge (N=3423)	Expert (N=211)
together. Both have equal authority.				
AI and physicians make the diagnosis together. The AI makes the final decision.	75 (4.56%)	165 (2.2%)	70 (2.18%)	4 (1.96%)
AI makes the diagnosis alone.	149 (9.07%)	256 (3.42%)	125 (3.9%)	23 (11.27%)
Q15, no. (%)	1616 (87.45%)	7466 (92.21%)	3186 (93.08%)	202 (95.73%)
Extremely negative	70 (4.33%)	163 (2.18%)	74 (2.32%)	8 (3.96%)
Rather negative	221 (13.68%)	503 (6.74%)	146 (4.58%)	9 (4.46%)
Neutral	578 (35.77%)	2351 (31.49%)	592 (18.58%)	24 (11.88%)
Rather positive	448 (27.72%)	3196 (73.56%)	1460 (45.83%)	54 (26.73%)
Extremely positive	299 (18.5%)	1254 (39.24%)	914 (28.69%)	107 (52.97%)
Q16, no. (%)	1601 (86.63%)	7366 (90.97%)	3156 (92.2%)	201 (95.26%)
Always prefer facilities without AI.	188 (11.74%)	398 (5.4%)	153 (4.85%)	10 (4.98%)
Tend to prefer facilities without AI.	438 (27.36%)	1831 (24.86%)	490 (15.53%)	19 (9.45%)
Rather prefer facilities with AI.	684 (42.72%)	4230 (57.43%)	1903 (60.3%)	105 (52.24%)
Always prefer facilities with AI.	291 (18.18%)	907 (12.31%)	610 (19.33%)	67 (33.33%)
Q17, no. (%)	1622 (87.77%)	7472 (92.28%)	3190 (93.19%)	204 (96.68%)
Very worried	363 (22.38%)	1267 (16.96%)	597 (18.71%)	48 (23.53%)
Somewhat concerned	427 (26.33%)	2804 (37.53%)	1062 (33.29%)	67 (32.84%)
Neutral	592 (36.5%)	2143 (28.68%)	777 (24.36%)	39 (19.12%)
Rather unconcerned	147 (9.06%)	909 (12.17%)	521 (16.33%)	29 (14.22%)
Unconcerned	93 (5.73%)	349 (4.67%)	233 (7.3%)	21 (10.29%)
Q18, no. (%)	1625 (87.93%)	7470 (92.26%)	3189 (93.16%)	203 (96.21%)
Very worried	470 (28.92%)	1819 (24.35%)	721 (22.61%)	45 (22.17%)
Somewhat concerned	555 (34.15%)	2947 (39.45%)	1077 (33.77%)	69 (33.99%)
Neutral	410 (25.23%)	1714 (22.95%)	762 (23.89%)	38 (18.72%)
Rather unconcerned	121 (7.45%)	772 (10.33%)	451 (14.14%)	39 (19.21%)
Unconcerned	69 (4.25%)	219 (2.93%)	178 (5.58%)	12 (5.91%)
Q19, no. (%)	1668 (90.26%)	7512 (92.78%)	3205 (93.63%)	205 (97.16%)
Very worried	563 (33.75%)	2304 (30.67%)	866 (27.02%)	47 (22.93%)
Somewhat concerned	474 (28.42%)	2490 (33.15%)	954 (29.77%)	60 (29.27%)
Neutral	393 (23.56%)	1511 (20.11%)	632 (19.72%)	28 (13.66%)
Rather unconcerned	152 (9.11%)	833 (11.09%)	522 (16.29%)	44 (21.46%)
Unconcerned	86 (5.16%)	374 (4.98%)	231 (7.21%)	26 (12.68%)
Q20, no. (%)	1662 (89.94%)	7505 (92.69%)	3203 (93.57%)	201 (95.26%)
Very worried	537 (32.31%)	1953 (26.02%)	713 (22.26%)	48 (23.88%)
Somewhat concerned	503 (30.26%)	2478 (33.02%)	907 (28.32%)	56 (27.86%)
Neutral	444 (26.71%)	2009 (26.77%)	383 (11.96%)	33 (16.42%)
Rather unconcerned	111 (6.68%)	734 (9.78%)	467 (14.58%)	26 (12.94%)
Unconcerned	67 (4.03%)	331 (4.41%)	378 (11.8%)	28 (13.9%)

Abbreviations: Q1, What are your general views on the use of artificial intelligence (AI) in medicine?; Q2, Artificial intelligence (AI) should be increasingly used in the healthcare sector.; Q3, How much confidence do you have that AI can improve healthcare?; Q4, How much do you trust an AI to provide reliable information about your health?; Q5, How much do you trust an

© 2025 Busch F et al. *JAMA Network Open*.

AI to provide accurate information about your diagnosis?; Q6, How much do you trust an AI to provide accurate information about your response to therapy?; Q7, Which of the following statements about a potential application of AI in medicine do you most likely agree with?; Q8, I would trust a highly accurate AI to make a vital decision for me.; Q9, How would you rate it if a certified AI software analyzes X-ray images?; Q10, How would you rate it if a certified AI software diagnoses cancer?; Q11, How would you rate it if a certified AI software would be available to doctors as a second opinion?; Q12/Q13, Suppose an AI makes a diagnosis. What would you prefer?; Q14, Suppose an AI has about the same accuracy as doctors. Which situation for a diagnosis would you prefer?; Q15, What do you think of healthcare facilities (clinics, practices) using AI software to aid in diagnosis?; Q16, Would you prefer to visit healthcare facilities that use AI software? Q17, How concerned are you about AI compromising the protection of your personal data?; Q18, How concerned are you that the use of AI will reduce the contact between physicians and patients?; Q19, How concerned are you that AI could replace human doctors in the future?; Q20, How concerned are you that the use of AI will lead to higher healthcare costs?.

© 2025 Busch F et al. *JAMA Network Open*.

eTable 14. Absolute survey results for each item stratified by median age and median number of technical devices used weekly

	Age ≤ median of 48 years (N=6287)	Age > median of 48 years (N=6165)	Technical devices used ≤ median of two (N=9611)	Technical devices > median of two (N=4141)
Q1, no. (%)	6171 (98.15%)	6049 (98.12%)	9390 (97.7%)	4105 (99.13%)
Extremely negative	158 (2.56%)	117 (1.93%)	220 (2.34%)	76 (1.85%)
Rather negative	441 (7.15%)	479 (7.92%)	793 (8.45%)	238 (5.8%)
Neutral	1747 (28.31%)	2245 (37.11%)	3369 (35.88%)	1025 (24.97%)
Rather positive	2622 (42.49%)	2431 (40.19%)	3592 (38.25%)	1962 (47.8%)
Extremely positive	1203 (19.49%)	777 (12.85%)	1416 (15.08%)	804 (19.59%)
Q2, no. (%)	6147 (97.77%)	5936 (96.29%)	9245 (96.19%)	4062 (98.09%)
Completely disagree	232 (3.77%)	158 (2.66%)	318 (3.44%)	110 (2.71%)
Tend to disagree	508 (8.26%)	468 (7.88%)	797 (8.62%)	261 (6.43%)
Neutral	1308 (21.28%)	1825 (30.74%)	2606 (28.19%)	835 (10.56%)
Tend to agree	2420 (39.37%)	2266 (38.17%)	3393 (36.7%)	1169 (28.78%)
Agree completely	1679 (27.31%)	1219 (20.54%)	2131 (23.05%)	1087 (26.76%)
Q3, no. (%)	6232 (99.13%)	6038 (97.94%)	9439 (98.21%)	4097 (98.94%)
Very little	244 (3.92%)	230 (3.81%)	415 (4.4%)	113 (2.76%)
Little	692 (11.1%)	731 (12.11%)	1201 (12.72%)	343 (8.37%)
Medium	2035 (32.65%)	2404 (39.81%)	3593 (38.07%)	1300 (31.73%)
Much	2233 (35.83%)	1937 (32.08%)	2941 (31.16%)	1625 (39.66%)
Very much	1028 (16.5%)	736 (12.19%)	1289 (13.66%)	716 (17.48%)
Q4, no. (%)	6225 (99.01%)	6013 (97.53%)	9401 (97.82%)	4100 (99.01%)
Very little	269 (4.32%)	243 (4.04%)	454 (4.83%)	110 (2.68%)
Little	785 (12.61%)	930 (15.47%)	1392 (14.81%)	466 (11.37%)
Medium	2314 (37.17%)	2331 (38.77%)	3673 (39.07%)	1473 (35.93%)
Much	2182 (35.05%)	2025 (33.68%)	2967 (31.56%)	1620 (39.51%)
Very much	675 (10.84%)	484 (8.05%)	915 (9.73%)	431 (10.51%)
Q5, no. (%)	6225 (99.01%)	6009 (97.47%)	9398 (97.78%)	4091 (98.79%)
Very little	267 (4.29%)	273 (4.45%)	459 (4.88%)	137 (3.35%)
Little	837 (13.45%)	928 (15.44%)	1448 (15.41%)	470 (11.49%)
Medium	2330 (37.43%)	2285 (38.03%)	3606 (38.37%)	1484 (36.27%)
Much	2127 (34.17%)	2008 (33.42%)	2927 (31.14%)	1589 (38.84%)
Very much	664 (10.67%)	515 (8.57%)	958 (10.19%)	411 (10.05%)
Q6, no. (%)	6221 (98.95%)	5997 (97.27%)	9385 (97.65%)	4088 (98.72%)
Very little	271 (4.36%)	292 (4.87%)	470 (5.01%)	143 (3.5%)
Little	838 (13.47%)	952 (15.87%)	1441 (15.35%)	520 (12.72%)
Medium	2416 (38.84%)	2366 (39.45%)	3758 (40.04%)	1507 (36.86%)
Much	2060 (33.11%)	1907 (31.8%)	2797 (29.8%)	1547 (37.84%)
Very much	636 (10.22%)	480 (8%)	919 (9.79%)	371 (9.08%)
Q7, no. (%)	6114 (97.25%)	5817 (94.36%)	9149 (95.19%)	3985 (96.23%)
No use of AI independent of the disease.	665 (10.88%)	669 (11.5%)	1239 (13.54%)	259 (6.5%)
Use only for minor illnesses (eg, cold).	1797 (29.39%)	1571 (27.01%)	2675 (26.24%)	684 (17.16%)
Use also for moderately severe diseases (eg, appendicitis).	1652 (27.02%)	1510 (25.96%)	2415 (26.4%)	1095 (27.48%)
Use also for severe diseases (eg, cancer, traffic accidents).	2000 (32.71%)	2067 (35.53%)	2820 (30.82%)	1647 (41.33%)
Q8, no. (%)	6206 (98.71%)	5979 (96.98%)	9348 (97.26%)	4083 (98.6%)
Completely disagree	640 (10.31%)	657 (10.99%)	958 (10.25%)	452 (11.07%)

© 2025 Busch F et al. *JAMA Network Open*.

eTable 14. Absolute survey results for each item stratified by median age and median number of technical devices used weekly

	Age ≤ median of 48 years (N=6287)	Age > median of 48 years (N=6165)	Technical devices used ≤ median of two (N=9611)	Technical devices > median of two (N=4141)
Tend to disagree	1252 (20.17%)	1111 (18.58%)	1718 (18.38%)	891 (21.82%)
Neutral	1520 (25.49%)	1816 (30.37%)	2717 (29.07%)	939 (23%)
Tend to agree	2097 (33.79%)	1869 (31.26%)	2913 (31.16%)	1460 (35.76%)
Agree completely	697 (11.23%)	526 (8.8%)	1042 (11.15%)	341 (8.35%)
Q9, no. (%)	6133 (97.55%)	5682 (92.17%)	9020 (93.85%)	3923 (94.74%)
Extremely negative	204 (3.33%)	151 (2.66%)	294 (3.26%)	84 (2.14%)
Rather negative	607 (9.9%)	521 (9.17%)	899 (9.97%)	322 (8.21%)
Neutral	1536 (25.04%)	1802 (31.71%)	2760 (30.6%)	909 (23.17%)
Rather positive	2522 (41.12%)	2320 (40.83%)	3467 (38.44%)	1821 (46.42%)
Extremely positive	1264 (20.61%)	888 (15.63%)	1600 (17.74%)	787 (20.06%)
Q10, no. (%)	6125 (97.42%)	5664 (91.87%)	8985 (93.49%)	3925 (94.78%)
Extremely negative	286 (4.67%)	232 (4.1%)	441 (4.91%)	121 (3.08%)
Rather negative	741 (12.1%)	708 (12.5%)	1158 (12.89%)	419 (10.68%)
Neutral	1710 (27.92%)	1673 (29.54%)	2715 (30.22%)	1009 (25.71%)
Rather positive	2295 (37.47%)	2178 (38.45%)	3183 (35.43%)	1709 (43.54%)
Extremely positive	1093 (17.84%)	873 (15.41%)	1488 (16.56%)	667 (16.99%)
Q11, no. (%)	6119 (97.33%)	5676 (92.07%)	8989 (93.53%)	3929 (94.88%)
Extremely negative	192 (3.14%)	124 (2.18%)	265 (2.95%)	77 (1.96%)
Rather negative	462 (7.55%)	303 (4.34%)	629 (7%)	204 (5.19%)
Neutral	1303 (21.29%)	1387 (24.44%)	2346 (26.1%)	617 (15.7%)
Rather positive	2450 (40.04%)	2453 (43.22%)	3623 (40.3%)	1741 (44.31%)
Extremely positive	1712 (27.98%)	1409 (24.82%)	2126 (23.65%)	1290 (32.83%)
Q12, no. (%)	6014 (95.66%)	5463 (88.61%)	8691 (90.43%)	3834 (92.59%)
A high degree of accuracy, the decision path is clearly comprehensible (explainable AI).	4327 (71.95%)	3702 (67.76%)	5892 (67.79%)	2897 (75.56%)
A higher accuracy, the decision path however, is not comprehensible.	1687 (28.05%)	1761 (32.24%)	2799 (32.21%)	937 (24.44%)
Q13, no. (%)	5948 (94.61%)	5286 (85.74%)	8483 (88.26%)	3753 (90.63%)
The AI misses almost no diagnosis, but often gives a false alarm.	2885 (48.5%)	2358 (44.61%)	3719 (43.84%)	1952 (52.01%)
The AI almost never gives a false alarm, but sometimes misses a diagnosis.	2079 (34.95%)	1965 (37.17%)	3224 (38.01%)	1199 (31.95%)
The AI gives a false alarm about as often as it misses a diagnosis.	984 (16.54%)	963 (18.22%)	1540 (18.15%)	568 (15.13%)
Q14, no. (%)	5939 (94.46%)	5579 (90.49%)	8752 (91.06%)	3861 (93.24%)
Physicians make the diagnosis alone.	337 (5.67%)	420 (7.53%)	678 (7.75%)	149 (3.86%)
AI and physicians make the diagnosis together. Doctors make the final decision.	4290 (72.23%)	4159 (74.55%)	6107 (69.78%)	3082 (79.82%)

© 2025 Busch F et al. *JAMA Network Open*.

eTable 14. Absolute survey results for each item stratified by median age and median number of technical devices used weekly

	Age ≤ median of 48 years (N=6287)	Age > median of 48 years (N=6165)	Technical devices used ≤ median of two (N=9611)	Technical devices > median of two (N=4141)
AI and physicians make the diagnosis together. Both have equal authority.	886 (14.92%)	680 (12.19%)	1254 (14.33%)	464 (12.02%)
AI and physicians make the diagnosis together. The AI makes the final decision.	139 (2.34%)	439 (7.87%)	265 (3.03%)	52 (1.35%)
AI makes the diagnosis alone.	287 (4.83%)	181 (3.24%)	448 (5.12%)	114 (2.95%)
Q15, no. (%)	5940 (94.48%)	5572 (90.38%)	8761 (91.16%)	3854 (93.07%)
Extremely negative	181 (3.05%)	122 (2.19%)	259 (2.96%)	61 (1.58%)
Rather negative	384 (6.46%)	445 (7.99%)	701 (8%)	188 (4.88%)
Neutral	1518 (25.56%)	1720 (30.87%)	2733 (31.2%)	853 (22.13%)
Rather positive	2472 (41.62%)	2300 (41.28%)	3390 (38.69%)	1820 (47.22%)
Extremely positive	1385 (23.32%)	985 (17.68%)	1677 (19.14%)	932 (24.18%)
Q16, no. (%)	5890 (93.69%)	5504 (89.28%)	8653 (90.03%)	3807 (91.93%)
Always prefer facilities without AI.	364 (6.18%)	354 (6.43%)	620 (7.17%)	152 (3.99%)
Tend to prefer facilities without AI.	1187 (20.15%)	1368 (24.85%)	2130 (24.62%)	660 (17.34%)
Rather prefer facilities with AI.	3361 (57.06%)	3088 (56.1%)	4559 (52.69%)	2445 (64.22%)
Always prefer facilities with AI.	978 (16.6%)	694 (12.61%)	1344 (15.53%)	550 (14.45%)
Q17, no. (%)	5938 (94.45%)	5593 (90.72%)	8772 (91.27%)	3859 (93.19%)
Very worried	1094 (18.42%)	1022 (18.27%)	1672 (19.06%)	644 (16.69%)
Somewhat concerned	2073 (34.91%)	1962 (35.08%)	3042 (34.68%)	1360 (35.24%)
Neutral	1630 (27.45%)	1623 (29.02%)	2605 (29.7%)	983 (25.47%)
Rather unconcerned	779 (13.12%)	698 (12.48%)	994 (11.33%)	623 (16.14%)
Unconcerned	362 (6.1%)	288 (5.15%)	459 (5.23%)	249 (6.45%)
Q18, no. (%)	5940 (94.48%)	5592 (90.71%)	8771 (91.26%)	3860 (93.21%)
Very worried	1334 (22.46%)	1530 (27.36%)	2229 (25.41%)	880 (22.8%)
Somewhat concerned	2078 (34.98%)	2201 (39.36%)	3227 (36.79%)	1462 (37.88%)
Neutral	1503 (25.3%)	1167 (20.87%)	2115 (24.11%)	838 (21.71%)
Rather unconcerned	730 (12.29%)	543 (9.71%)	880 (10.03%)	512 (13.26%)
Unconcerned	295 (4.97%)	151 (2.7%)	320 (3.65%)	168 (4.35%)
Q19, no. (%)	6037 (96.02%)	5600 (90.84%)	8868 (92.27%)	3866 (93.36%)
Very worried	1597 (26.45%)	1921 (34.3%)	2764 (31.17%)	1088 (28.14%)
Somewhat concerned	1850 (30.64%)	1838 (32.82%)	2763 (31.16%)	1244 (32.18%)
Neutral	1327 (21.98%)	1033 (18.45%)	1900 (21.43%)	688 (17.80%)
Rather unconcerned	833 (13.8%)	585 (10.45%)	982 (11.07%)	585 (15.13%)
Unconcerned	430 (7.12%)	223 (3.98%)	459 (5.18%)	261 (6.75%)
Q20, no. (%)	6030 (95.91%)	5589 (90.66%)	8851 (92.09%)	3862 (93.26%)
Very worried	1518 (25.17%)	1545 (27.64%)	2436 (27.52%)	886 (22.94%)
Somewhat concerned	1883 (31.23%)	1794 (32.1%)	5690 (64.29%)	1133 (29.34%)
Neutral	1486 (24.64%)	1517 (27.14%)	2340 (26.44%)	1005 (26.02%)
Rather unconcerned	725 (12.02%)	503 (9%)	800 (9.04%)	557 (14.42%)

© 2025 Busch F et al. *JAMA Network Open*.

eTable 14. Absolute survey results for each item stratified by median age and median number of technical devices used weekly

	Age ≤ median of 48 years (N=6287)	Age > median of 48 years (N=6165)	Technical devices used ≤ median of two (N=9611)	Technical devices > median of two (N=4141)
Unconcerned	418 (6.93%)	230 (4.12%)	429 (4.85%)	281 (7.28%)

Abbreviations: Q1, What are your general views on the use of artificial intelligence (AI) in medicine?; Q2, Artificial intelligence (AI) should be increasingly used in the healthcare sector.; Q3, How much confidence do you have that AI can improve healthcare?; Q4, How much do you trust an AI to provide reliable information about your health?; Q5, How much do you trust an AI to provide accurate information about your diagnosis?; Q6, How much do you trust an AI to provide accurate information about your response to therapy?; Q7, Which of the following statements about a potential application of AI in medicine do you most likely agree with?; Q8, I would trust a highly accurate AI to make a vital decision for me.; Q9, How would you rate it if a certified AI software analyzes X-ray images?; Q10, How would you rate it if a certified AI software diagnoses cancer?; Q11, How would you rate it if a certified AI software would be available to doctors as a second opinion?; Q12/Q13, Suppose an AI makes a diagnosis. What would you prefer?; Q14, Suppose an AI has about the same accuracy as doctors. Which situation for a diagnosis would you prefer?; Q15, What do you think of healthcare facilities (clinics, practices) using AI software to aid in diagnosis?; Q16, Would you prefer to visit healthcare facilities that use AI software? Q17, How concerned are you about AI compromising the protection of your personal data?; Q18, How concerned are you that the use of AI will reduce the contact between physicians and patients?; Q19, How concerned are you that AI could replace human doctors in the future?; Q20, How concerned are you that the use of AI will lead to higher healthcare costs?.

© 2025 Busch F et al. *JAMA Network Open*.

eTable 15. Absolute survey results for each item stratified by highest educational level

	**Elementary School (N=1192)**	**Middle School (N=2844)**	**High School (N=4142)**	**University (N=5403)**
Q1, no. (%)	1151 (96.56%)	2767 (97.29%)	4058 (97.97%)	5368 (99.35%)
Extremely negative	36 (3.13%)	67 (2.42%)	617 (15.2%)	122 (2.27%)
Rather negative	91 (7.91%)	253 (9.14%)	1729 (42.61%)	333 (6.2%)
Neutral	496 (43.49%)	1074 (38.81%)	1304 (32.13%)	1454 (27.09%)
Rather positive	357 (31.02%)	1005 (36.32%)	1729 (42.61%)	2416 (45.01%)
Extremely positive	171 (14.86%)	368 (13.3%)	617 (15.2%)	1043 (19.43%)
Q2, no. (%)	1095 (91.86%)	2697 (94.83%)	4055 (97.9%)	5317 (98.41%)
Completely disagree	53 (4.84%)	95 (3.52%)	111 (2.74%)	159 (2.99%)
Tend to disagree	82 (7.49%)	257 (9.53%)	352 (8.68%)	358 (6.73%)
Neutral	377 (34.43%)	838 (31.07%)	1029 (25.38%)	1151 (21.65%)
Tend to agree	333 (30.41%)	965 (35.78%)	1588 (39.16%)	2225 (41.85%)
Agree completely	250 (22.83%)	542 (20.1%)	975 (24.04%)	1424 (26.78%)
Q3, no. (%)	1084 (90.94%)	2793 (98.21%)	4091 (98.77%)	5354 (99.09%)
Very little	70 (6.46%)	114 (4.08%)	164 (4.01%)	168 (3.14%)
Little	156 (14.39%)	395 (14.14%)	503 (12.3%)	468 (8.74%)
Medium	454 (41.88%)	1109 (39.71%)	1467 (35.86%)	1805 (33.71%)
Much	293 (27.03%)	853 (30.54%)	1463 (35.76%)	1316 (24.58%)
Very much	172 (15.07%)	322 (11.53%)	464 (11.34%)	997 (18.62%)
Q4, no. (%)	1140 (95.64%)	2775 (97.57%)	4085 (98.62%)	5353 (99.07%)
Very little	72 (6.32%)	145 (5.23%)	170 (4.16%)	165 (3.08%)
Little	173 (15.18%)	396 (14.27%)	566 (13.86%)	698 (13.04%)
Medium	474 (41.58%)	1190 (42.88%)	1517 (37.14%)	1900 (35.49%)
Much	286 (25.09%)	825 (29.73%)	1501 (36.74%)	1946 (36.35%)
Very much	135 (11.84%)	219 (7.89%)	331 (8.1%)	644 (12.03%)
Q5, no. (%)	1139 (95.55%)	2771 (97.43%)	4086 (98.65%)	5347 (98.96%)
Very little	76 (6,67%)	136 (4.91%)	179 (4.38%)	187 (3.5%)
Little	172 (15.1%)	414 (14.94%)	607 (14.86%)	704 (13.17%)
Medium	455 (39.95%)	1174 (42.37%)	1496 (36.61%)	1910 (35.72%)
Much	287 (25.2%)	808 (29.16%)	1484 (36.32%)	1903 (35.59%)
Very much	149 (13.08%)	239 (8.63%)	320 (7.83%)	643 (12.03%)
Q6, no. (%)	1132 (94.97%)	2774 (97.54%)	4080 (98.5%)	5342 (98.87%)
Very little	67 (5.92%)	152 (5.48%)	172 (4.22%)	205 (3.84%)
Little	174 (15.37%)	409 (14.74%)	630 (15.44%)	727 (13.81%)
Medium	460 (40.64%)	1193 (43.01%)	1586 (38.87%)	1966 (36.8%)
Much	301 (26.59%)	777 (28.01%)	1382 (33.87%)	1850 (34.63%)
Very much	130 (11.48%)	241 (8.69%)	310 (7.6%)	594 (11.12%)
Q7, no. (%)	1090 (91.44%)	2697 (94.83%)	3996 (96.48%)	5224 (96.69%)
No use of AI independent of the disease.	242 (22.2%)	378 (4.02%)	437 (10.94%)	421 (8.06%)
Use only for minor illnesses (eg, cold).	314 (28.81%)	809 (30%)	1123 (28.1%)	1379 (26.4%)
Use also for moderately severe diseases (eg, appendicitis).	149 (13.67%)	723 (26.81%)	1125 (28.15%)	1366 (26.15%)
Use also for severe diseases (eg, cancer, traffic accidents).	285 (26.15%)	787 (29.18%)	1301 (32.56%)	2058 (39.4%)
Q8, no. (%)	1128 (94.63%)	2758 (96.98%)	4079 (98.48%)	5322 (%)
Completely disagree	138 (12.23%)	323 (11.71%)	427 (10.47%)	501 (9.41%)
Tend to disagree	160 (14.18%)	577 (20.92%)	825 (20.23%)	1018 (19.13%)
Neutral	373 (33.07%)	785 (28.46%)	1097 (26.89%)	1367 (25.69%)
Tend to agree	318 (28.19%)	776 (28.14%)	1359 (33.32%)	1881 (35.34%)

© 2025 Busch F et al. *JAMA Network Open*.

eTable 15. Absolute survey results for each item stratified by highest educational level

	Elementary School (N=1192)	Middle School (N=2844)	High School (N=4142)	University (N=5403)
Agree completely	139 (12.32%)	297 (10.77%)	371 (9.1%)	555 (10.43%)
Q9, no. (%)	1061 (89.01%)	2632 (92.55%)	4001 (96.6%)	5102 (94.43%)
Extremely negative	45 (4.24%)	86 (3.27%)	114 (2.85%)	121 (2.37%)
Rather negative	104 (9.8%)	350 (13.3%)	368 (9.2%)	391 (7.66%)
Neutral	368 (34.68%)	830 (31.53%)	1152 (28.79%)	1266 (24.81%)
Rather positive	342 (32.23%)	347 (13.18%)	1701 (42.51%)	2249 (44.08%)
Extremely positive	202 (19.04%)	419 (15.92%)	666 (16.65%)	1075 (21.07%)
Q10, no. (%)	1058 (88.76%)	2620 (92.12%)	3994 (96.43%)	5092 (94.24%)
Extremely negative	56 (5.29%)	680 (25.95%)	175 (4.38%)	183 (3.59%)
Rather negative	116 (10.96%)	382 (14.58%)	444 (11.12%)	620 (12.18%)
Neutral	362 (34.22%)	806 (30.76%)	1165 (29.17%)	1346 (26.43%)
Rather positive	337 (31.85%)	911 (34.77%)	1560 (39.06%)	2037 (40%)
Extremely positive	187 (17.67%)	385 (14.69%)	650 (16.27%)	906 (17.79%)
Q11, no. (%)	1052 (88.26%)	2623 (92.23%)	3991 (96.35%)	5106 (94.5%)
Extremely negative	38 (3.61%)	66 (2.52%)	97 (2.43%)	133 (2.6%)
Rather negative	59 (5.61%)	186 (7.09%)	283 (7.09%)	298 (5.84%)
Neutral	320 (30.42%)	727 (27.72%)	875 (21.92%)	998 (19.55%)
Rather positive	405 (38.5%)	1082 (41.25%)	1741 (43.62%)	2083 (40.8%)
Extremely positive	230 (21.86%)	562 (21.43%)	995 (24.93%)	1594 (31.22%)
Q12, no. (%)	1004 (84.23%)	2493 (87.66%)	3885 (93.89%)	5010 (92.73%)
A high degree of accuracy, the decision path is clearly comprehensible (explainable AI).	666 (66.33%)	1743 (69.92%)	2773 (71.38%)	3524 (70.34%)
A higher accuracy, the decision path however, is not comprehensible.	338 (33.67%)	750 (30.08%)	1112 (28.62%)	1486 (29.66%)
Q13, no. (%)	958 (80.37%)	2441 (85.83%)	3816 (92.13%)	4900 (90.69%)
The AI misses almost no diagnosis, but often gives a false alarm.	393 (41.02%)	1110 (45.47%)	1749 (45.83%)	2381 (48.59%)
The AI almost never gives a false alarm, but sometimes misses a diagnosis.	375 (39.14%)	897 (36.75%)	1376 (36.06%)	1751 (35.73%)
The AI gives a false alarm about as often as it misses a diagnosis.	190 (19.83%)	434 (17.78%)	691 (18.11%)	768 (15.67%)
Q14, no. (%)	1028 (86.24%)	2537 (89.21%)	3888 (93.87%)	5017 (92.86%)
Physicians make the diagnosis alone.	82 (7.98%)	134 (5.28%)	252 (6.48%)	212 (4.23%)
AI and physicians make the diagnosis together. Doctors make the final decision.	632 (61.48%)	1731 (68.23%)	2818 (72.48%)	3917 (78.07%)
AI and physicians make the diagnosis together. Both have equal authority.	161 (15.66%)	351 (13.84%)	598 (15.38%)	589 (11.74%)
AI and physicians make the diagnosis together.	39 (3.79%)	84 (3.31%)	92 (2.37%)	94 (1.87%)

© 2025 Busch F et al. *JAMA Network Open*.

eTable 15. Absolute survey results for each item stratified by highest educational level

	Elementary School (N=1192)	Middle School (N=2844)	High School (N=4142)	University (N=5403)
The AI makes the final decision.				
AI makes the diagnosis alone.	114 (11.09%)	237 (9.34%)	128 (3.29%)	205 (4.09%)
Q15, no. (%)	1033 (86.66%)	2563 (90.12%)	3895 (94.04%)	4982 (92.21%)
Extremely negative	33 (3.19%)	63 (2.46%)	101 (2.59%)	118 (2.37%)
Rather negative	51 (4.94%)	222 (8.66%)	232 (5.96%)	372 (7.47%)
Neutral	406 (39.3%)	918 (35.82%)	1130 (29.01%)	1077 (21.62%)
Rather positive	337 (32.62%)	923 (36.01%)	1693 (43.47%)	2208 (44.32%)
Extremely positive	206 (19.94%)	437 (17.05%)	739 (18.97%)	1207 (24.23%)
Q16, no. (%)	1009 (84.65%)	2538 (89.24%)	3843 (92.78%)	4941 (91.45%)
Always prefer facilities without AI.	102 (10.11%)	203 (8%)	226 (5.88%)	228 (4.61%)
Tend to prefer facilities without AI.	267 (26.46%)	729 (28.72%)	905 (23.55%)	861 (17.43%)
Rather prefer facilities with AI.	455 (45.09%)	1248 (49.17%)	2204 (57.35%)	3027 (61.26%)
Always prefer facilities with AI.	185 (18.33%)	358 (14.11%)	508 (13.22%)	825 (16.7%)
Q17, no. (%)	1032 (86.58%)	2559 (89.98%)	3907 (94.33%)	4991 (92.37%)
Very worried	190 (18.41%)	434 (16.96%)	802 (20.53%)	855 (17.13%)
Somewhat concerned	318 (30.81%)	875 (34.19%)	1424 (36.45%)	1739 (34.84%)
Neutral	350 (33.91%)	751 (29.35%)	1038 (26.57%)	1411 (28.27%)
Rather unconcerned	108 (10.47%)	335 (13.09%)	445 (11.39%)	714 (14.31%)
Unconcerned	66 (6.4%)	164 (6.41%)	198 (5.07%)	272 (5.45%)
Q18, no. (%)	1030 (86.41%)	2565 (90.19%)	3902 (94.21%)	4994 (92.43%)
Very worried	249 (24.17%)	635 (24.76%)	1099 (28.17%)	1076 (21.55%)
Somewhat concerned	352 (34.17%)	806 (31.42%)	1420 (36.39%)	1960 (39.25%)
Neutral	279 (27.09%)	640 (24.95%)	872 (22.35%)	1135 (22.73%)
Rather unconcerned	102 (9.9%)	266 (10.37%)	389 (9.97%)	627 (12.56%)
Unconcerned	49 (4.76%)	116 (4.52%)	122 (3.13%)	196 (3.92%)
Q19, no. (%)	1039 (87.16%)	2580 (90.72%)	3950 (95.36%)	5024 (92.99%)
Very worried	341 (32.82%)	815 (31.59%)	1367 (34.61%)	1277 (25.42%)
Somewhat concerned	300 (28.87%)	748 (28.99%)	1251 (31.67%)	1659 (33.02%)
Neutral	235 (22.62%)	549 (21.28%)	793 (20.08%)	971 (19.33%)
Rather unconcerned	111 (10.68%)	295 (11.43%)	372 (9.42%)	775 (14.43%)
Unconcerned	52 (5%)	153 (5.93%)	167 (4.23%)	342 (6.81%)
Q20, no. (%)	1038 (87.08%)	2582 (90.79%)	3951 (95.39%)	5002 (92.58%)
Very worried	302 (29.09%)	670 (25.95%)	1225 (31%)	1076 (21.51%)
Somewhat concerned	284 (27.36%)	758 (29.36%)	1253 (31.71%)	1640 (32.79%)
Neutral	301 (29%)	747 (28.93%)	977 (24.73%)	1289 (25.77%)
Rather unconcerned	99 (9.54%)	271 (10.5%)	338 (8.55%)	641 (12.81%)
Unconcerned	52 (5.01%)	136 (5.27%)	158 (4%)	356 (7.12%)

Abbreviations: Q1, What are your general views on the use of artificial intelligence (AI) in medicine?; Q2, Artificial intelligence (AI) should be increasingly used in the healthcare sector.; Q3, How much confidence do you have that AI can improve healthcare?; Q4, How much do you trust an AI to provide reliable information about your health?; Q5, How much do you trust an AI to provide accurate information about your diagnosis?; Q6, How much do you trust an AI to provide accurate information about your response to therapy?; Q7, Which of the following statements about a potential application of AI in medicine do you most likely agree with?; Q8, I would trust a highly accurate AI to make a vital decision for me.; Q9, How would you rate it if a certified AI software analyzes X-ray images?; Q10, How would you rate it if a certified AI software diagnoses cancer?; Q11, How would you rate it if a certified AI software would be available to doctors as a second opinion?; Q12/Q13, Suppose an AI makes a diagnosis. What would you prefer?; Q14, Suppose an AI has about the same accuracy as doctors. Which situation for a diagnosis would you prefer?; Q15, What do you think of healthcare facilities (clinics, practices) using AI software to aid in diagnosis?; Q16, Would you prefer to visit healthcare facilities that use AI software? Q17, How concerned are you about AI compromising the protection of your personal data?; Q18, How concerned are you that the use of AI will reduce the contact

© 2025 Busch F et al. *JAMA Network Open*.

between physicians and patients?; Q19, How concerned are you that AI could replace human doctors in the future?; Q20, How concerned are you that the use of AI will lead to higher healthcare costs?.

© 2025 Busch F et al. *JAMA Network Open*.

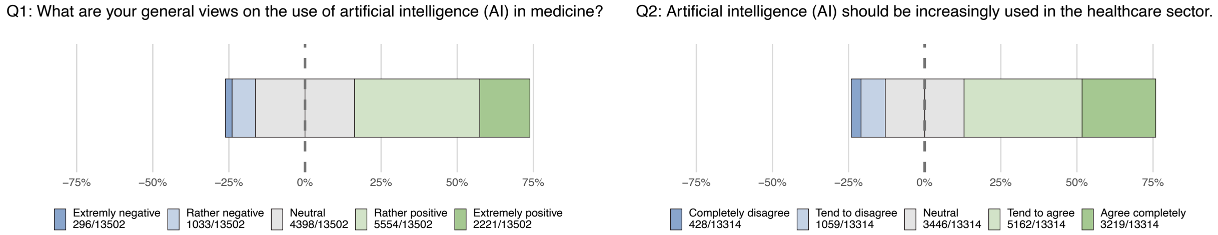

eFigure 1. Gantt diagrams depicting the results for each item in the "General attitudes towards AI" section

Colors represent the response options indicated below each Gantt chart, including the corresponding numerator and denominator.

© 2025 Busch F et al. *JAMA Network Open*.

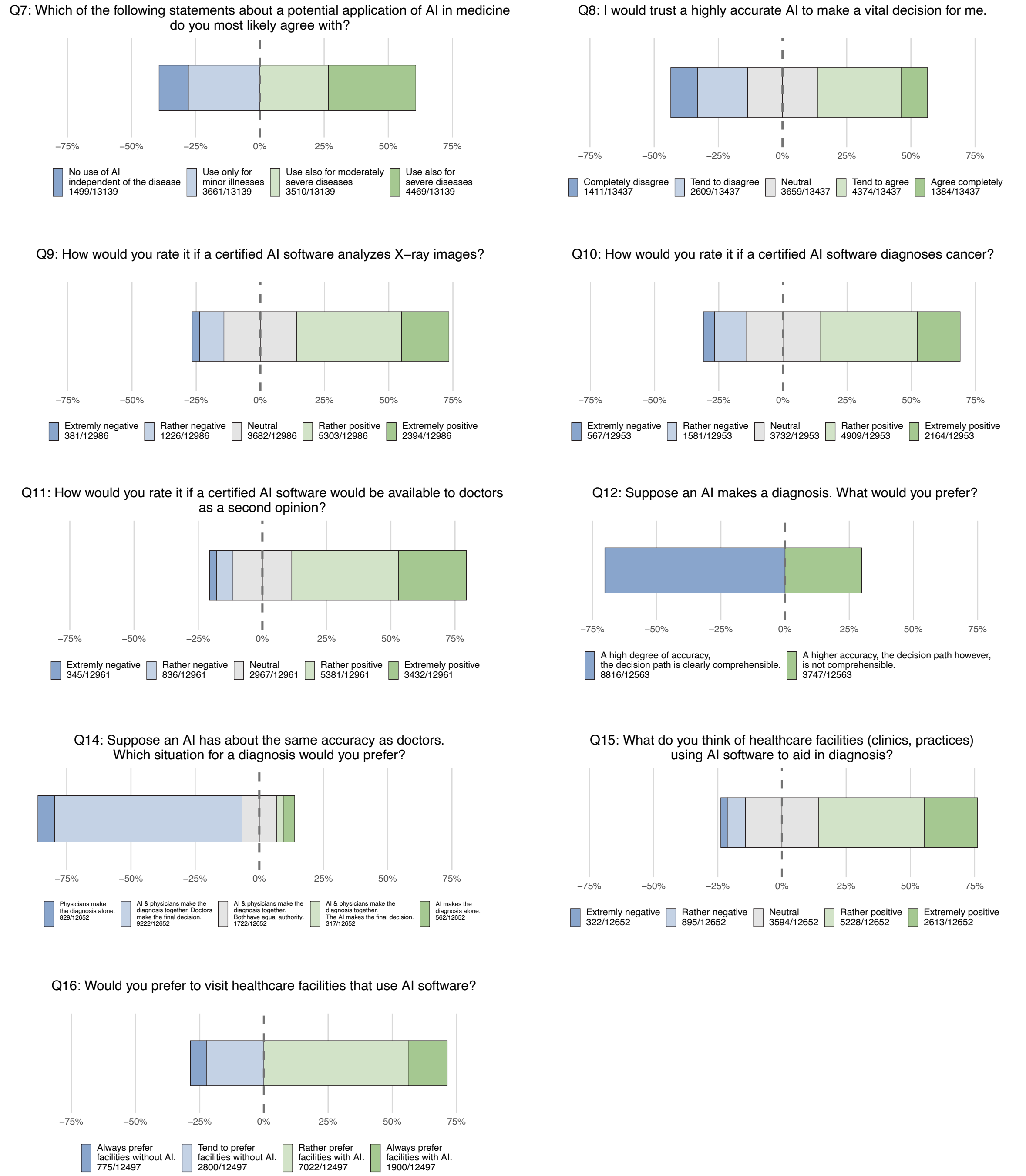

eFigure 2. Gantt diagrams depicting the results for each item in the "Preferences towards AI applications in diagnostics and healthcare facilities" section

Colors represent the response options indicated below each Gantt chart, including the corresponding numerator and denominator.

© 2025 Busch F et al. *JAMA Network Open*.
