# Supplementary material for: Multinational Attitudes Toward AI in Health Care and Diagnostics Among Hospital Patients
Source: JAMA Netw Open. 2025 Jun 10;8(6):e2514452. doi: 10.1001/jamanetworkopen.2025.14452 (PMC12152705; doi:10.1001/jamanetworkopen.2025.14452)
Supplement: Supplement 2. — Data Sharing Statement [file jamanetwopen-e2514452-s002.pdf]

Data Sharing Statement

Busch. Multinational Attitudes Toward AI in Health Care and Diagnostics Among Hospital Patients. *JAMA Netw Open*. Published June 10, 2025.
doi:10.1001/jamanetworkopen.2025.14452

Data
Data available: Yes
Data types: Deidentified participant data, Data dictionary
How to access data: The full dataset and a data dictionary are publicly available under CC-BY 4.0 international license at figshare: https://doi.org/10.6084/m9.figshare.24964488.
When available: beginning date: 09-01-2024

Supporting Documents
Document types: Statistical/analytic code
How to access documents: The code for all statistical analyses is publicly available from our GitHub repository: https://gist.github.com/kbressem/7028613a6a16ad9594a18f0c1b85a0ee
When available: With publication

Additional Information
Who can access the data: To anyone under CC-BY 4.0 international license.
Types of analyses: For any purpose.
Mechanisms of data availability: Without investigator support.
